# One-Pot Cannizzaro Cascade Synthesis of *ortho*-Fused Cycloocta-2,5-dien-1-ones from 2-Bromo(hetero)aryl Aldehydes

**DOI:** 10.1002/anie.201505347

**Published:** 2015-07-29

**Authors:** Laurence Burroughs, Lee Eccleshare, John Ritchie, Omkar Kulkarni, Barry Lygo, Simon Woodward, William Lewis

**Affiliations:** School of Chemistry, University of Nottingham University Park, Nottingham NG7 2RD (UK)

**Keywords:** aldehydes, annulation, carbocycles, medium-ring compounds, synthetic methods

## Abstract

An intramolecular Cannizzaro-type hydride transfer to an in situ prepared allene enables the synthesis of ortho-fused 4-substituted cycloocta-2,5-dien-1-ones with unprecedented technical ease for an eight-ring carboannulation. Various derivatives could be obtained from commercially available (hetero)aryl aldehydes, trimethylsilylacetylene, and simple propargyl chlorides in good yields.

Readily (often commercially) available 2-bromo(hetero)aryl aldehydes (**1**, Scheme 1) constitute powerful and popular units for the construction of *ortho*-fused carbocyclic rings.^1^ Two strategies commonly employed in the multitudinous syntheses described in the literature involve 1) the derivatization of **1** to intermediates of type **A**, with a π-system attacking an electrophile that is ultimately derived from the aldehyde by functional-group manipulations (e.g., an alcohol, acetal, or even the aldehyde itself); or 2) formyl group homologation to an anion-stabilizing unit that attacks a suitably activated π-bond (**B**). Whereas many elegant catalytic systems that use intermediates **A** or **B** have already been described,^2^, ^3^ the preparation of the suitable precursors is often step-intensive, raising questions on the overall process efficiency in the “age of sustainability”.^4^ We speculated that a new annulation strategy, based on a Cannizzaro-type reaction via intermediate **C**, might be possible. As the hydride transfer simultaneously exposes a powerful Michael acceptor (ynone or equivalent) and a carbanion in close proximity, efficient annulation might be expected to occur (especially if **C** is directly attained in situ). Herein, we describe the use of such a strategy for the formation of eight-membered rings. The paucity of direct single-pot/step procedures to such medium rings attests to the known issues associated with their synthesis;^5^ they therefore provide a stringent test for strategy C. Although redox-based Cannizzaro reactions, including asymmetric and triggered C–C bond-forming versions, have recently been developed,^6^ to the best of our knowledge, they have not been employed for carboannulation reactions to medium rings. Genuine single-pot/step procedures to eight-membered rings are very limited—Reppe’s historical catalytic access to cyclooctatetraene^7^ and Murakami’s [4+2+2] approach^8^ being notable exceptions. Modern alternatives frequently describe catalytic cyclizations that proceed with exquisitely high yields (for the final step). Unfortunately, their overall synthetic efficiency is often compromised by the multistep syntheses required to obtain the cyclization precursors.^9^

**Scheme 1:**
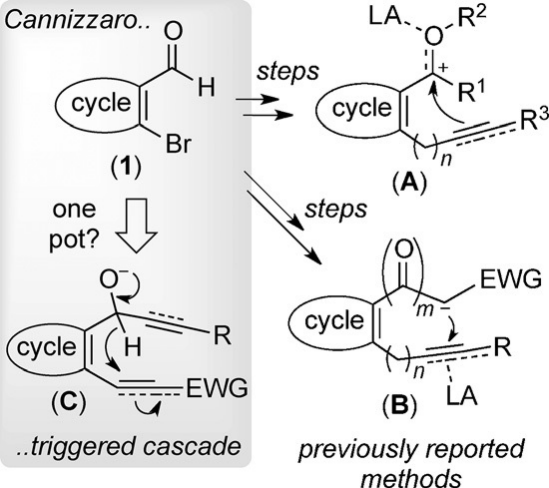
Proposed use of 2-bromo(hetero)aryl aldehydes in Cannizzaro-triggered annulation cascades compared to traditional approaches. EWG=electron-withdrawing group, LA=Lewis acid, R^1^–R^3^ are generic groups, *n* and *m* are typically 0–1.

Initial investigation of our own proposal (C) centered on the combination of readily available 2-bromobenzaldehyde (**1 a**), trimethylsilylacetylene (**2 a**), and propargyl chloride **3 a**. Encouragingly, even an initial run returned significant amounts of benzo[8]annulene derivative **4 aaa** (Table 1, entry 1, ca. 7 %) whose identity and regiochemistry was confirmed by X-ray crystallography (see the Supporting Information) as initial NMR data was not conclusive.^10^ Unfortunately, under the conditions of entry 1, multiple side products were also produced. Upon changing from an organocopper to an organocuprate formulation (entry 2), the reaction became much cleaner, but the yield of **4 aaa** remained low. The recovered side products of entry 2 included significant amounts of the alcohols **5 a** and **6 a** together with unreacted **3 a**. We could confirm that formation of **D** was almost quantitative in <20 min under all conditions tried (Et_2_O or THF, −78 to 0 °C, based on recovered **5 a** in independent reactions). We therefore suspected issues associated with both *n*BuLi–halogen exchange from **D** and the copper-promoted S_N_2′ reaction leading to the progenitor for eight-ring formation. Further experiments were designed to probe these hypotheses (Table 1).

**Table 1:** Optimization of the Cannizzaro-triggered cascade reaction to eight-ring compound 4 aaa.^[a]^ 
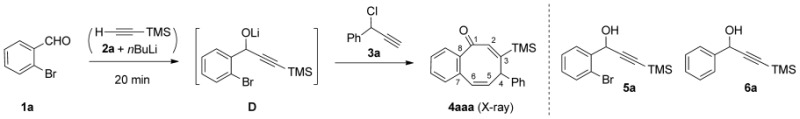

Entry	Solvent	Halogen exchange of intermediateD	Transmetalation with CuBr⋅SMe_2_	Addition of3 aand cyclization	Yield4 aaa [%]
1	Et_2_O	*t*BuLi (2 equiv), −50 °C, 20 min	1 equiv, −50 °C, 20 min	1 equiv, −50 °C to RT over 5 h	<10
2	Et_2_O	*n*BuLi (1 equiv), 0 °C, 10 min	0.5 equiv, −50 °C, 5 min	0.5 equiv, −50 °C to +10 °C over 1.2 h	14^[b]^
3	THF	*n*BuLi (1 equiv), −50 °C, 15 min	0.5 equiv, −50 °C, 10 min	0.5 equiv, −50 °C to 10 °C over 1.2 h	53
4	THF	*n*BuLi (1 equiv), −50 °C, 15 min	0.5 equiv, −50 °C, 1 h	0.5 equiv, −50 °C to −10 °C over 1.5 h; kept at −10 °C for 1 h	70 (69)^[c]^
5	THF	*n*BuLi (1 equiv), −50 °C, 15 min	0.5 equiv, −50 °C, 1 h	0.5 equiv, −50 °C to −10 °C over 1.5 h; kept at −10 °C for 2 h	35

[a] Reaction conditions: **1 a** (0.71 mmol), **2 a** (0.77 mmol)/*n*BuLi (0.72 mmol), CuBr⋅SMe_2_ (0.36–0.71 mmol), **3 a** (0.35–0.70 mmol) in Et_2_O or THF (1.0 mL). The yields of **4 aaa** were determined by GC analysis against genuine samples and an internal standard (1-methylnaphthalene, 50 μL). [b] The mass balance of the reaction included **3 a**, **5 a**, and **6 a** in a 2.4:1.0:1.8 ratio. [c] The yield of isolated product for a reaction performed with 1.39 mmol of **3 a** is shown in parentheses.

Organometallic compounds derived from **D** were poorly soluble in Et_2_O so the reaction solvent was switched to THF (entry 3). This led to an improved reaction that enabled us to identify that the onset of the Cannizzaro-type cascade reaction occurs at −20 to −10 °C for this substrate. Independent treatment of THF solutions containing **D** with *n*BuLi followed by quenching with D_2_O confirmed that complete Br/Li exchange had occurred within 15 min at −40 to −60 °C, and that the resulting organolithium species was rather stable at these temperatures. However, above approximately −15 °C, the same species began to decompose rapidly. Thus, −40 to −50 °C was selected as the temperature range for optimizing the transmetalation to CuBr⋅SMe_2_. When the nominal diorganocuprate was prepared under the conditions of entry 3 (−50 °C, 10 min, from the aryl lithium intermediate with CuBr⋅SMe_2_) and then treated with methyl vinyl ketone, very poor conversions into the 1,4-addition product were attained. This strongly suggested that transmetalation to Cu^I^ is rather slow for this system. Gratifyingly, extending the time for Li/Cu exchange to one hour (entry 4) significantly increased the quantity of **4 aaa** formed, and the GC yields were comparable to those attained through isolation (70 % vs. 69 %). Further extending the transmetalation time to two hours at −10 °C had a detrimental effect on the yield of **4 aaa** (entry 5).

With an optimized procedure for the preparation of parent compound **4 aaa** in hand, we studied the scope of the reaction with respect to the 2-bromo(hetero)aryl aldehyde component **1**. Using the conditions of entry 4 (Table 1) and aldehydes **1 b**–**1 j**, the eight-ring compounds that are shown in Scheme 2 were attained. Substitution at the 3-position was better tolerated than at the 1- and 4-positions (products **4 baa**/**4 caa** vs. **4 daa**/**4 faa**). Monosubstitution at the 2-postion was also tolerated. A comparison of the yields of **4 baa**–**4 gaa** indicates that steric factors are more important than electronic ones; for example, intramolecular OMe steric contacts increase the *ortho* steric profile of **4 eaa** versus **4 jaa**. The yields of compounds **4 haa**–**4 jaa** also support this trend. Whereas the yields of **4 aaa**–**4 jaa** are modest, it should be borne in mind that they represent one-pot processes to analytically pure crystalline products on up to multigram scales, using only inexpensive commercial starting materials. The new process compares very favorably to the consolidated yields and time efficiencies of previously reported eight-ring syntheses.^9^

**Scheme 2:**
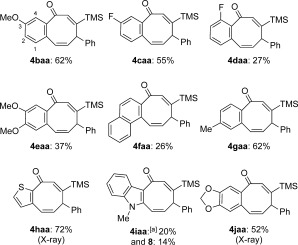
Variation of the 2-bromo(hetero)aryl aldehyde; yields starting from the commercial aldehydes are given.^9^ [a] The precursor aldehyde was obtained from isatin (3 steps).

By varying the acetylene component using the readily attained starting materials **2 b**–**2 f**, five further examples of eight-ring compounds **4** were synthesized (Scheme 3). The terminal alkyne substituent could encompass alkyl, vinyl, or aryl units, and the corresponding products were all obtained in acceptable yields. Finally, variation of the propargyl chloride was investigated using simple **3 b**–**3 i** (Scheme 3). The aryl moiety could accommodate both electron-donating and -withdrawing substituents and some functional groups (**4 aae** and **4 aaf**).

**Scheme 3:**
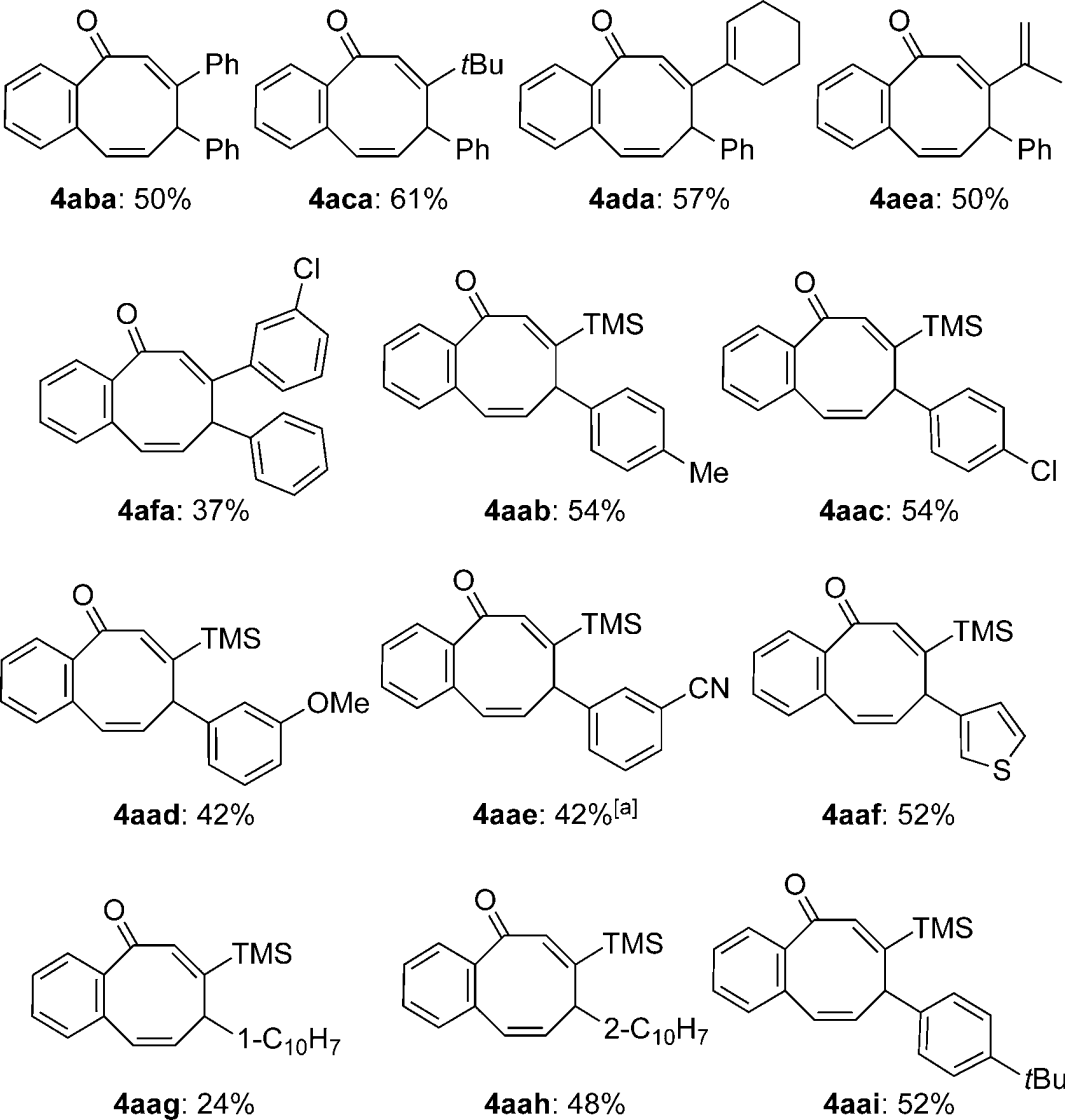
Variation of the terminal acetylene and propargyl chloride. [a] Run at −50 °C.

Whereas the full mechanistic features of our Cannizzaro-triggered cascade are still under investigation, a working hypothesis is shown in Scheme 4. Sequential 1,2-acetylide addition, halogen exchange, and transmetalation to copper affords intermediate **E**. This copper(I) species undergoes a γ-selective S_N_2′ addition to propargyl chloride **3** affording the non-isolable allene **C′**, which undergoes the Cannizzaro-type hydride shift proposed in Scheme 1. The resulting allyl anion is presumably placed in close proximity to the potent ynone Michael acceptor, which leads to efficient ring closure. The use of an aryl moiety as the R^2^ substituent is apparently sufficient to stabilize the allyl anion and allow the necessary *trans* to *cis* isomerization. Protonation of the non-classical enolate **G** results in the formation of **4**. Several pieces of evidence support this chain of events. First, attempts to use 2-thienylcyanocuprates derived from a related aza substrate led to the isolation of **7** (X-ray structure) where the intermediate allene has been carbometalated rather than having accepted a hydride. For the formation of **4 iaa**, we could isolate appreciable amounts of **8**, which is the expected product of a Cannizzaro-type hydride transfer without subsequent annulation. Use of the deuterated aldehyde 2-BrC_6_H_4_CDO ([D]-**1 a**) led to the formation of the expected product [D]-**4 aaa**, confirming the participation of the aldehyde in the Cannizzaro process.

**Scheme 4:**
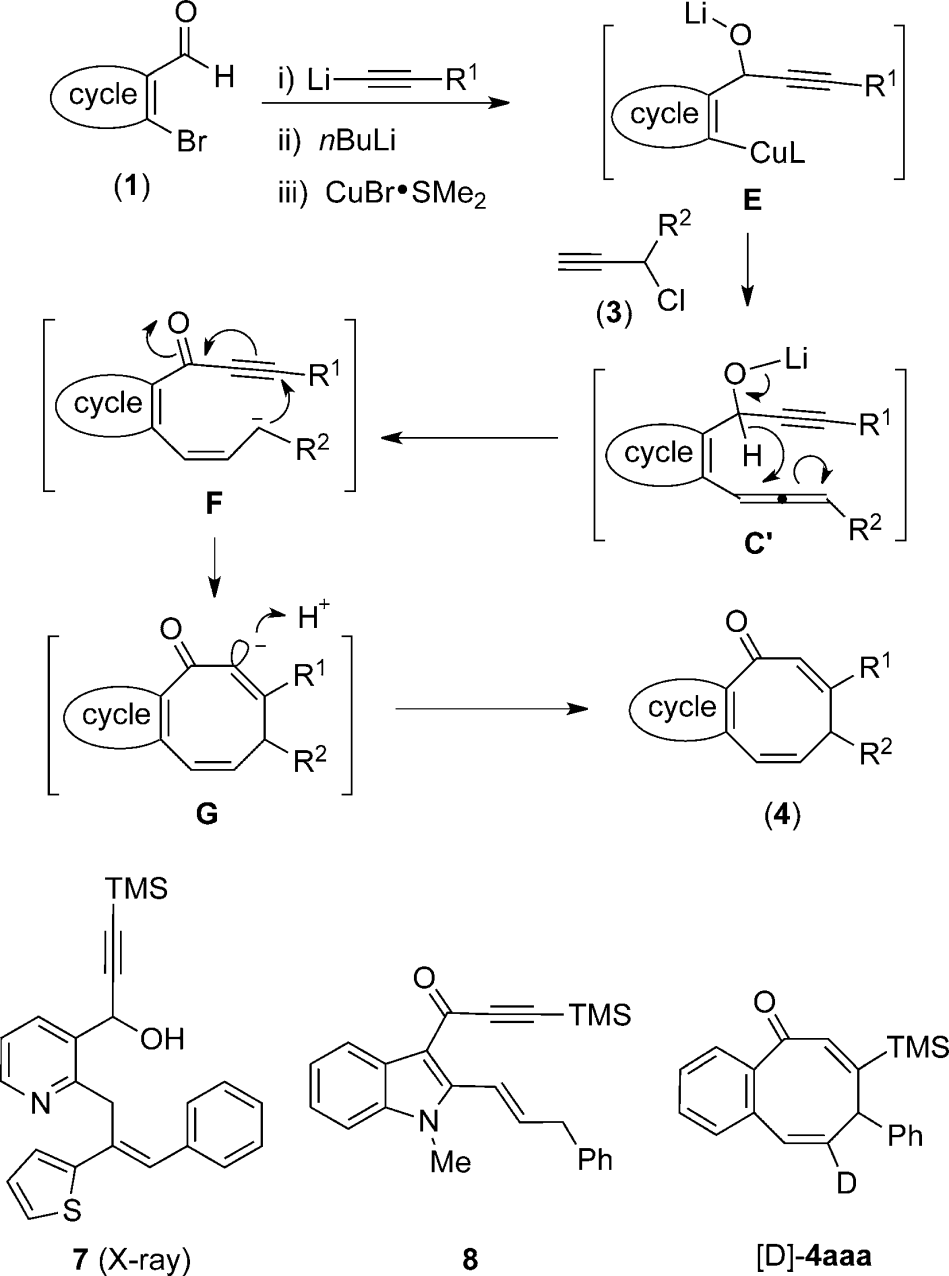
Mechanistic proposal for the formation of eight-ring compounds 4; L is indicative of the presence of a generic organocopper or -cuprate species.

The mechanistic proposal shown in Scheme 4 was also probed by DFT calculations at the M06-2X/6-31G(d) and CBS-QB3 levels of theory^11^ on the “metal-free” anions *anti*-**C′** and *syn*-**C′** (Scheme 5; see also the Supporting Information). Whereas the relationship of *anti*-**C′** and *syn*-**C′** to the reaction coordinate of the true organometallic species involved in the cascade process needs to be treated with caution, it does provide further evidence for the proposed mechanism. The calculated barriers for the Cannizzaro-type hydride transfer and the rearrangement of the allyl anion (the equivalent of the transformation of **C′** into **F** in Scheme 4) were approximately 5 and 7 kcal mol^−1^, respectively. The calculations also indicate that non-classical enolates of type **G** are favored in the reaction over the equivalent allenoate structures. This idea was further reinforced by the observation that trapping of the reaction intermediates with iodine, iodomethane, or carbon dioxide, as opposed to a proton, leads to the formation of derivatives **9**–**11** (Scheme 5).

**Scheme 5:**
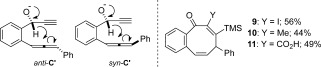
The computational models *anti*-C′ and *syn*-C′ and indirect evidence for the formation of G.

To the best of our knowledge, this Cannizzaro-triggered cascade process is unique. The nearest analogues that we could find are cyclizations reported by the Oonishi and Sato groups;9b,d however, in these cases, the allene intermediate must be separately synthesized, and no Cannizzaro-type process is involved. Similarly, a single eight-ring side product (formed in 10 % yield) was obtained by Alajarin and Vidal through a 1,5-hydride shift/8π electrocyclization from a neutral 1,2-allenylacetal at 110 °C.^12^ Again, no Cannizzaro-type trigger was proposed. Whereas Cannizzaro shifts have been used for oxidation-state manipulations in *ortho*-fused systems, in these processes, no annulation steps were involved.^13^ The most similar ynone eight-ring closure process that could be identified was developed by Schreiber and Kelly,^14^ but this transformation involves an alkoxide nucleophile.

In conclusion, we believe that this very simple formation of *ortho*-fused cycloocta-2,5-dien-1-one units demonstrates the potential of the developed method for rapid and efficient annulations. Investigations into extending the scope of both the present reaction and other variants are ongoing.

## Supporting Information

As a service to our authors and readers, this journal provides supporting information supplied by the authors. Such materials are peer reviewed and may be re-organized for online delivery, but are not copy-edited or typeset. Technical support issues arising from supporting information (other than missing files) should be addressed to the authors.

miscellaneous_information
