# Supplementary material for: One-Pot Cannizzaro Cascade Synthesis of ortho-Fused Cycloocta-2,5-dien-1-ones from 2-Bromo(hetero)aryl Aldehydes
Source: Angew Chem Int Ed Engl. 2015 Jul 29;54(36):10648–51. doi: 10.1002/anie.201505347 (PMC4581465; doi:10.1002/anie.201505347)

Angewandte Chemie
Eine Zeitschrift der Gesellschaft Deutscher Chemiker

Supporting Information

One-Pot Cannizzaro Cascade Synthesis of *ortho*-Fused Cycloocta-2,5-dien-1-ones from 2-Bromo(hetero)aryl Aldehydes

Laurence Burroughs, Lee Eccleshare, John Ritchie, Omkar Kulkarni, Barry Lygo, Simon Woodward, and William Lewis*

anie_201505347_sm_miscellaneous_information.pdf

Contents

**1. General Information**	**1**
**2. Preparation of starting materials**	**1**
**3. General procedure for the one-pot carboannulation to 4**	**8**
**4. Computational studies**	**20**
**5. References**	**50**
**6. Spectroscopic data**	**53**
**7. Selected crystallographic data**	**80**

1. General information

All reactions were carried out under an atmosphere of argon using flame-dried Schlenk tubes. Dichloromethane was used distilled from calcium hydride; diethyl ether and tetrahydrofuran were distilled from sodium/benzophenone ketyl under argon. Aldehydes were purchased from Sigma-Aldrich and were used as received. Alkynes were purchased from Sigma-Aldrich and were distilled before use. Cooling was achieved using a Haake DC50-K75 refrigerated circulator or a Huber TC50E immersion cooler. All temperatures are referred to the temperatures of the cooling baths used. Thin layer chromatography was performed on foil-backed plates coated with Merck Silica gel 60 F254. The plates were developed using ultraviolet light and basic aqueous potassium permanganate. Liquid chromatography was performed using forced flow (flash column) with the solvent systems indicated. The stationary phase used was silica gel 60 (220–240 mesh) supplied by Fluorochem. Infrared spectra were recorded on a Bruker Tensor 27 FT-IR spectrometer using NaCl plates. Nuclear magnetic resonance spectra were recorded on a Bruker DPX-400 (400.2 MHz), Bruker AV400 (400.1 MHz), Bruker AV(III)400 (400.1 MHz) or a Bruker AV(III)500 (500.1 MHz) spectrometer at ambient temperature; chemical shifts are quoted in parts per million (ppm) and were referenced as follows: chloroform-d, 7.26 ppm for ^{1}H NMR data; chloroform-d, 77.16 ppm for ^{13}C NMR data; dimethyl sulfoxide-d$_6$, 2.50 ppm for ^{1}H NMR data; dimethyl sulfoxide-d$_6$, 39.52 ppm for ^{13}C NMR data.[S1] Deuterium NMR were run in $CHCl_3$ with $CDCl_3$ added for reference (7.26 ppm). Coupling constants (*J*) are quoted in Hertz. Mass spectrometry was performed using a VG Micromass AutoSpec spectrometer (EI) or Bruker MicroTOF (ESI) as noted.

2. Preparation of starting materials

Supplies of α-phenylpropargyl chlorides (**3a**) were prepared from the commercial alcohol using Appel reactions by a literature method.[S2] Simple α-arylpropargyl chlorides, (**3b**-**3i**), are chlorination products of

the parent alcohols *via* Appel methods or *via* addition of thionyl chloride. The parent alcohols were attained through addition of HC≡CMgBr to suitable aldehydes. Yields for the alcohols and chlorides are summarised in Table S1. Representative experimental procedures for the preparation of α-arylpropargyl chlorides are given. All other components for the subsequent carboannulations were commercially available except aldehyde **1i** whose synthesis is detailed herein.

Table S1. Preparations of required propargyl chlorides (**3**).

Compound	Aldehyde	Organometallic	Yield alcohol/%	Chlorination method	Yield chloride (**3**)/%[a]
**3a**	PhCHO	HC≡CMgBr	>99	$SOCl_2$	86
**3b**	(4-MePh)CHO	HC≡CMgBr	>99	Appel	81
**3c**	(4-ClPh)CHO	HC≡CMgBr	80	Appel	97
**3d**	(3-MeOPh)CHO	HC≡CMgBr	>99	$SOCl_2$	>99
**3e**	(3-NCPh)CHO	HC≡CMgBr	92	$SOCl_2$	83
**3f**	(3-thiophenyl)CHO	HC≡CMgBr	96	$SOCl_2$	92
**3g**	(1-naphthyl)CHO	HC≡CMgBr	89	$SOCl_2$	75
**3h**	(2-naphthyl)CHO	HC≡CMgBr	99	$SOCl_2$	86
**3i**	(4-*t*BuPh)CHO	HC≡CMgBr	95	$SOCl_2$	89

[a] In the case of inseparable S_N2:S_N2' mixtures the yield of propargylic component **3** was determined by NMR techniques.

General procedure for Appel reaction

Parent alcohol was dissolved in tetrahydrofuran under argon to make a 1.1 M solution. Triphenylphosphine (1.20 molar equivalents) was then added and the solution cooled to 0 °C. *N*-Chlorosuccinimide (1.25 molar equivalents) was next dissolved in tetrahydrofuran under argon to give a 0.6 M solution, and this solution added dropwise over 15 minutes to the reaction mixture. After completion of the addition the reaction mixture was heated to reflux for 18 hours, after which time the solution was cooled to room temperature and pentane added. The reaction mixture was then filtered and the filtrate concentrated to provide the crude product.

General procedure for chlorination using thionyl chloride

Parent alcohol was dissolved in dichloromethane under argon to make a 0.35 M solution. This was cooled to 0 °C before thionyl chloride (5 molar equivalents) was added dropwise to the reaction mixture over 10 minutes. After stirring for 2 hours the reaction mixture was allowed to warm to room temperature and stirred for a further 1 hour. The reaction mixture was then concentrated *in vacuo* to afford the crude product.

(1-Chloroprop-2-yn-1-yl)benzene (3a)

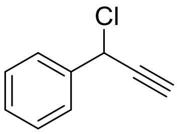

(**3a**)

General thionyl chloride procedure. Afforded the title compound as an inseparable 17:1 mixture of S_N2:S_N2' products (1-chloroprop-2-yn-1-yl)benzene and (3-chloropropa-1,2-dien-1-yl)benzene. Providing (1-chloroprop-2-yn-1-yl)benzene in 86% yield as a pale yellow oil. **Rf** (pentane) 0.72; **IR** (NaCl, film) υ_{max} 3295, 2126, 1647, 1494, 1455, 1270, 1193 cm^{-1}; **^{1}H NMR** (400.2 MHz, $CDCl_3$): δ 7.60 – 7.57 (m, 2H, Ar*H*), 7.42 – 7.34 (m, 3H, Ar*H*), 5.66 (d, *J* = 2.4 Hz, 1H, ArC*H*Cl), 2.86 (d, *J* = 2.4 Hz, 1H, CC*H*); **^{13}C NMR** (100.6 MHz, $CDCl_3$) δ 138.1 (C), 129.3 (CH), 129.0 (CH), 127.7 (CH), 80.7 (C), 77.0 (CH), 49.2 (CH); **MS** (EI+): m/z 115 $[M-Cl^-]^+$; HRMS: found 115.0554 $C_9H_7^+$ requires 115.0542 Compound characterisation data was found to be in agreement with literature values.[S2]

1-(1-Chloroprop-2-yn-1-yl)-4-methylbenzene (3b)

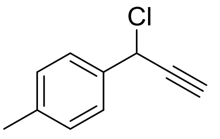

(**3b**)

General Appel reaction procedure afforded the title compound as an inseparable 7:1 mixture of S_N2:S_N2' products 1-(1-chloroprop-2-yn-1-yl)-4-methylbenzene and 1-(1-chloropropa-1,2-dien-1-yl)-4-methylbenzene. Providing 1-(1-chloroprop-2-yn-1-yl)-4-methylbenzene in 81% yield as a pale yellow oil. **IR** (NaCl, film) υ_{max} 3295, 3029, 2865, 1614, 15121198, 1181, 837, 820, 726 cm^{-1}; **^{1}H NMR** (400.2 MHz, $CDCl_3$): δ 7.47 (d, *J* = 8.0 Hz, 2H, Ar*H*), 7.20 (d, *J* = 8.0 Hz, 2H, Ar*H*), 5.64 (d, *J* = 2.3 Hz, 1H, ArC*H*Cl), 2.84 (d, *J* = 2.3 Hz, 1H, CC*H*), 2.37 (s, 3H, ArCH_3); **^{13}C NMR** (100.6 MHz, $CDCl_3$) δ 139.4 (C), 136.3 (C), 129.7 (CH), 127.6 (CH), 80.9 (C), 76.7 (CH), 49.2 (CH), 21.4 (CH_3); **MS** (EI+): m/z 129 $[M-Cl^-]^+$; HRMS: found 129.0745 $C_{10}H_9^+$ requires 129.0699.

1-Chloro-4-(1-chloroprop-2-yn-1-yl)benzene (3c)

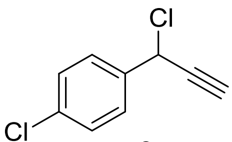

(**3c**)

General Appel reaction procedure afforded the title compound as an inseparable 32:1 mixture of S_N2:S_N2' products 1-chloro-4-(1-chloroprop-2-yn-1-yl)benzene and 1-chloro-4-(1-chloropropa-1,2-dien-1-yl)benzene. Providing 1-chloro-4-(1-chloroprop-2-yn-1-yl)benzene in 97% yield as a pale yellow oil. **IR** (NaCl, film) υ_{max} 3297, 2955, 2127, 1597, 1491, 1408, 1195, 1093, 1016, 989, 835, 822, 729, 687 cm^{-1}; **^{1}H NMR** (400.2 MHz, $CDCl_3$): δ 7.55 – 7.49 (m, 2H, Ar*H*), 7.40 – 7.34 (m, 2H, Ar*H*), 5.62 (d, *J* = 2.4 Hz, 1H, ArC*H*Cl), 2.89 (d, *J* = 2.4 Hz, 1H, CC*H*); **^{13}C NMR** (100.6 MHz, $CDCl_3$) δ 136.6 (C), 135.3 (C), 129.2 (CH), 129.1 (CH), 80.2 (C), 77.3 (CH), 48.3 (CH); **MS** (EI+): m/z 149 $[M-Cl^-]^+$; HRMS: found 149.0178 $C_9H_6Cl^+$ requires 149.0153.

1-(1-Chloroprop-2-yn-1-yl)-3-methoxybenzene (3d)

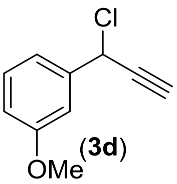

(**3d**)

General thionyl chloride procedure afforded the title compound 1-(1-chloroprop-2-yn-1-yl)-3-methoxybenzene in quantitative yield as a brown oil. **IR** (NaCl, film) υ_{max} 3291, 3004, 2960, 2837, 2124, 1602, 1587, 1491, 1466, 1455, 1435, 1318, 1282, 1265, 1160, 1042, 715 cm^{-1}; **^{1}H NMR** (400.2 MHz, $CDCl_3$): δ 7.34 – 7.28 (m, 1H, Ar*H*), 7.18 – 7.12 (m, 2H, Ar*H*), 6.90 (ddd, *J* = 8.3, 2.6, 1.0 Hz, 1H, Ar*H*), 5.62 (d, *J* = 2.5 Hz, 1H, ArC*H*Cl), 3.84 (s, 3H, OCH_3), 2.85 (d, *J* = 2.5 Hz, 1H, CC*H*); **^{13}C NMR** (100.6 MHz, $CDCl_3$) δ 160.0 (C), 139.5 (C), 130.0 (CH), 120.0 (CH), 115.0 (CH), 113.2 (CH), 80.6 (C), 76.9 (CH), 55.5 (CH_3), 49.1 (CH); **MS** (EI+): m/z 145 $[M-Cl^-]^+$; HRMS: found 145.0631 $C_{10}H_9O^+$ requires 145.0648.

3-(1-Chloroprop-2-yn-1-yl)benzonitrile (3e)

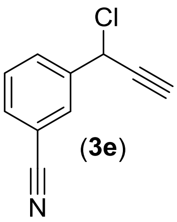

(**3e**)

General thionyl chloride procedure afforded the title compound as an inseparable 6:1 mixture of S_N2:S_N2' products 3-(1-chloroprop-2-yn-1-yl)benzonitrile and 3-(1-chloropropa-1,2-dien-1-yl)benzonitrile. Providing 3-(1-chloroprop-2-yn-1-yl)benzonitrile in 83% yield as a brown oil. **IR** (NaCl, film) υ_{max} 3293, 3065, 2232, 1481, 1434, 1269, 1147, 804, 714, 681 cm^{-1}; **^{1}H NMR** (400.2 MHz, $CDCl_3$): δ 7.92 – 7.89 (m, 1H, Ar*H*), 7.85 – 7.78 (m, 1H, Ar*H*), 7.69 – 7.64 (m, 1H, Ar*H*), 7.53 (app t, *J* = 7.9 Hz, 1H, Ar*H*), 5.65 (d, *J* = 2.3 Hz, 1H, ArC*H*Cl), 2.92 (d, *J* = 2.3 Hz, 1H, CC*H*); **^{13}C NMR** (125.0 MHz, $CDCl_3$) δ 139.6 (C), 132.8 (CH), 132.1 (CH), 131.3 (CH), 129.9 (CH), 118.2 (C), 113.2 (C), 74.9 (C), 78.1 (CH), 47.6 (CH); **MS** (EI+): m/z 373 $[2M+Na]^+$; HRMS: found 373.0266 $C_{20}H_{12}Cl_2N_2Na^+$ requires 373.0270.

3-(1-Chloroprop-2-yn-1-yl)thiophene (3f)

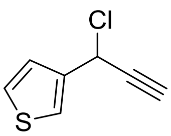

(**3f**)

General thionyl chloride procedure afforded the title compound as an inseparable 11:1 mixture of S_N2:S_N2' products 3-(1-chloroprop-2-yn-1-yl)thiophene and 3-(1-chloropropa-1,2-dien-1-yl)thiophene. Providing 3-(1-chloroprop-2-yn-1-yl)thiophene in 92% yield as a brown oil. **IR** (NaCl, film) υ_{max} 3292, 3105, 1671, 1419, 1290, 824, 782, 744, 704 cm^{-1}; **^{1}H NMR** (400.2 MHz, $CDCl_3$): δ 7.51 – 7.48 (m, 1H, Ar*H*), 7.34 (dd, *J* = 5.1, 3.0 Hz, 1H, Ar*H*), 7.23 (dd, *J* = 5.1, 1.1 Hz, 1H, Ar*H*), 5.73 (d, *J* = 2.4 Hz, 1H, ArC*H*Cl), 2.82 (d, *J* = 2.4 Hz, 1H, CC*H*); **^{13}C NMR** (100.6

MHz, $CDCl_3$) δ 138.7 (C), 127.2 (CH), 126.8 (CH), 124.5 (CH), 80.6 (C), 75.9 (CH), 44.2 (CH); **MS** (EI): m/z 155 [M]; HRMS: found 155.9803 C_7H_5ClS requires 155.9800.

1-(1-Chloroprop-2-yn-1-yl)naphthalene (3g)

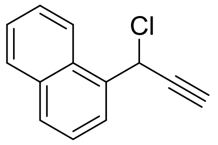

(**3g**)

General thionyl chloride procedure afforded the title compound as an inseparable 4:1 mixture of S_N2:S_N2' products 1-(1-chloroprop-2-yn-1-yl)naphthalene and. 1-(3-chloropropa-1,2-dien-1-yl)naphthalene. Providing 1-(1-chloroprop-2-yn-1-yl)naphthalene in 75% yield as a brown oil. **IR** (NaCl, film) $υ_{max}$ 3293, 3054, 1597, 1511, 966, 800, 779 cm^{-1}; ^{1}**H NMR** (400.2 MHz, $CDCl_3$): δ 8.29 – 8.24 (m, 1H, Ar*H*), 8.01 – 7.83 (m, 3H, Ar*H*), 7.68 – 7.45 (m, 3H, Ar*H*), 6.35 (d, *J* = 2.4 Hz, 1H, ArC*H*Cl), 2.95 (d, *J* = 2.4 Hz, 1H, CC*H*); ^{13}**C NMR** (100.6 MHz, $CDCl_3$) δ 134.2 (C), 132.9 (C), 130.6 (CH), 130.0 (C), 129.2 (CH), 127.0 (CH), 126.5 (CH), 126.4 (CH), 125.3 (CH), 123.4 (CH), 80.6 (C), 71.3 (CH), 47.2 (CH); **MS** (ESI+): m/z 165 $[M-Cl^-]^+$; HRMS: found 165.0700 $C_{13}H_9^-$ requires 165.0699.

2-(1-Chloroprop-2-yn-1-yl)naphthalene (3h)

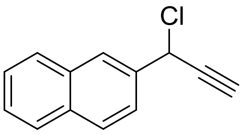

(**3h**)

General thionyl chloride procedure afforded the title compound as an inseparable 13:1 mixture of S_N2:S_N2' products 2-(1-chloroprop-2-yn-1-yl)naphthalene and 2-(3-chloropropa-1,2-dien-1-yl)naphthalene. Providing 2-(1-chloroprop-2-yn-1-yl)naphthalene in 86% yield as a brown solid. **mp** 62-64 °C; **IR** (NaCl, film) $υ_{max}$ 3293, 3058, 1600, 1508, 860, 818, 751, 705 cm^{-1}; ^{1}**H NMR** (400.2 MHz, $CDCl_3$): δ 8.03 (d, *J* = 1.2 Hz, 1H, Ar*H*), 7.92 – 7.78 (m, 3H, Ar*H*), 7.68 (dd, *J* = 8.6, 1.9 Hz, 1H, Ar*H*), 7.57 – 7.47 (m, 2H, Ar*H*), 5.83 (d, *J* = 2.4 Hz, 1H, ArC*H*Cl), 2.93 (d, *J* = 2.4 Hz, 1H, CC*H*); ^{13}**C NMR** (100.6 MHz, $CDCl_3$) δ 135.3 (C), 133.6 (C), 133.1 (C), 129.2 (CH), 128.5 (CH), 127.9 (CH), 127.1 (CH), 126.8 (2 x CH), 125.1 (CH), 80.7 (C), 77.3 (CH), 49.5 (CH); **MS** (ESI+): m/z 165 $[M-Cl^-]^+$; HRMS: found 165.0707 $C_{13}H_9^-$ requires 165.0699.

1-(*tert*-Butyl)-4-(1-chloroprop-2-yn-1-yl)benzene (3i)

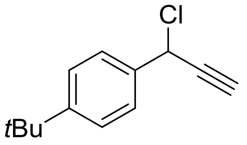

(**3i**)

General thionyl chloride procedure afforded the title compound as an inseparable 11:1 mixture of S_N2:S_N2' products 1-(*tert*-butyl)-4-(1-chloroprop-2-yn-1-yl)benzene and 1-(*tert*-butyl)-4-(3-chloropropa-1,2-dien-1-yl)benzene. Providing 1-(*tert*-butyl)-4-(1-chloroprop-2-yn-1-yl)benzene in 89% yield as a brown oil. **IR** (NaCl, film) 3295, 2964, 2904, 2869, 2126, 1509, 1461, 1270, 1364, 839, 817, 691 cm^{-1}; ^{1}**H NMR** (400.2 MHz, $CDCl_3$): δ 7.55 – 7.48 (m, 2H, Ar*H*), 7.45 – 7.39 (m, 2H, Ar*H*), 5.65 (d, *J* = 2.4 Hz, 1H, ArC*H*Cl), 2.85 (d, *J* = 2.4 Hz, CC*H*), 1.33 (s, 9H, C(C*H*$_3$)$_3$); ^{13}**C NMR** (100.6 MHz, $CDCl_3$) δ 152.6 (C), 153.1 (C), 127.4 (CH), 126.0 (CH), 80.9 (C), 76.7 (CH), 49.1 (CH), 34.9 (C), 31.4 (CH_3); **MS** (ESI+): m/z 171 $[M-Cl^-]^+$; HRMS: found 171.1083 $C_{13}H_{15}^+$ requires 171.1168.

General Procedure for synthesis of alcohol precursors for 3a-3f

Starting aldehyde was dissolved in tetrahydrofuran under argon to make a 2 M solution and cooled to 0 °C. Ethynylmagnesium bromide (0.5 M in tetrahydrofuran, 1.1 molar equivalents) was added, turning the solution orange from colourless. After completion of the addition the reaction mixture was warmed to room temperature. After 3 hours the reaction mixture was quenched with saturated ammonium chloride solution and extracted with ethyl acetate. The combined organic extracts were dried with magnesium sulfate, filtered and concentrated *in vacuo* to provide the crude product.

Precursor alcohol for synthesis of 3a: 1-phenylprop-2-yn-1-ol

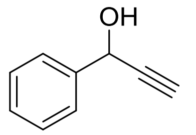

Precursor for (**3a**)

Following the general procedure the title compound 1-phenylprop-2-yn-1-ol was afforded in >99% yield as a brown oil. **Rf** (9:1 pentane:diethyl ether) 0.47; **IR** ($CHCl_3$, solution) υ_{max} 3592, 3306, 2121, 1494, 1454, 1017, 942, 649 cm-1 **^{1}H NMR** (400.2 MHz, $CDCl_3$): δ 7.60 – 7.52 (m, 2H, Ar*H*), 7.44 – 7.31 (m, 3H, Ar*H*), 5.48 (d, *J* = 2.2 Hz, 1H, ArC*H*OH), 2.68 (d, *J* = 2.2 Hz, 1H, CC*H*), 2.06 (br s, 1H, ArCHO*H*); **^{13}C NMR** (100.6 MHz, $CDCl_3$) δ 140.1 (C), 128.7 (CH), 128.5 (CH), 126.7 (CH), 83.6 (C), 74.9 (CH), 64.3 (CH); **MS** (ESI+): m/z 132 [M]; HRMS: found 132.0572 C_9H_8O requires 132.0575. Compound characterisation data was found to be in agreement with literature values[S3] and was found to be identical to the reagent as purchased from Sigma-Aldrich (Cat. No. 226610-1G, CAS Number: 4187-87-5).

Precursor alcohol for synthesis of 3b: 1-(p-tolyl)prop-2-yn-1-ol

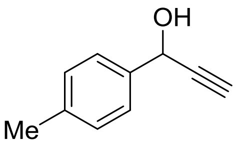

Precursor for (**3b**)

Following the general procedure the title compound 1-(p-tolyl)prop-2-yn-1-ol was afforded in >99% yield as a brown oil. **IR** (NaCl, film) υ_{max} 3530, 3364, 3290, 2117, 1614, 1513, 1179, 1016, 947, 817 cm^{-1}; **^{1}H NMR** (400.2 MHz, $CDCl_3$): δ 7.44 (d, *J* = 7.9 Hz, 2H, Ar*H*), 7.20 (d, *J* = 7.9 Hz, 2H, Ar*H*), 5.44 (dd, *J* = 6.2, 2.1 Hz, 1H, ArC*H*OH), 2.66 (d, *J* = 2.1 Hz, 1H, CC*H*), 2.36 (s, 3H, ArC*H*$_3$), 2.11 (d, *J* = 6.2 Hz, 1H, O*H*); **^{13}C NMR** (100.6 MHz, $CDCl_3$) 128.5 (C), 137.2 (C), 129.4 (CH), 126.6 (CH), 83.7 (C), 74.7 (CH), 64.3 (CH), 21.2 (CH_3); **MS** (ESI+): m/z 169 $[M+Na]^+$; HRMS: found 169.0626 $C_{10}H_{10}NaO^+$ requires 169.0624. Compound characterisation data was found to be in agreement with literature values.[S3]

Precursor alcohol for synthesis of 3c: 1-(4-chlorophenyl)prop-2-yn-1-ol

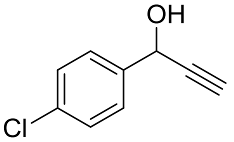

Precursor for (**3c**)

Following the general procedure the title compound 1-(4-chlorophenyl)prop-2-yn-1-ol was afforded in 80% yield as a brown oil. **IR** (NaCl, film) υ_{max} 3550, 3346, 3297, 2120, 1597, 1491, 1406, 1092, 1015, 950, 835, 792 cm^{-1}; **^{1}H NMR** (400.2 MHz, $CDCl_3$): δ 7.53 – 7.47 (m, 2H, Ar*H*), 7.39 – 7.34 (m, 2H, Ar*H*), 5.49 – 5.42 (m, 1H, ArC*H*OH), 2.69 (d, *J* = 2.2 Hz, 1H, CC*H*), 2.21 (br d, *J* = 5.5 Hz, 1H, O*H*); **^{13}C NMR** (100.6 MHz, $CDCl_3$) 138.6 (C), 134.5 (C), 128.9 (CH), 128.1 (CH), 83.2 (C), 75.3 (CH), 63.8 (CH); **MS** (EI): m/z 166 [M]; HRMS: found 166.0185 C_9H_7ClO requires 166.0185 Compound characterisation data was found to be in agreement with literature values.[S4]

Precursor alcohol for synthesis of 3d: 1-(3-methoxyphenyl)prop-2-yn-1-ol

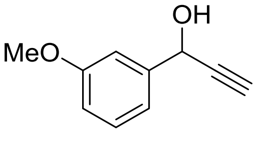

Precursor for (**3d**)

Following the general procedure where addition of Grignard was carried out at -35 °C, warmed to room temperature and stirred for 17 hours. The title compound 1-(3-methoxyphenyl)prop-2-yn-1-ol was afforded in >99% yield as a brown oil. **Rf** (1:1 pentane:diethyl ether) 0.48; **IR** (NaCl, film) υ_{max} 3394, 3287, 3004, 2942, 1601, 1588, 1488, 1261, 1037 cm^{-1}; **^{1}H NMR** (400.2 MHz, $CDCl_3$): δ 7.31 (ddd, *J* = 8.2, 7.4, 0.6 Hz, 1H, Ar*H*), 7.16 – 7.09 (m, 2H, Ar*H*), 6.89 (ddd, *J* = 8.2, 2.5, 1.1 Hz, 1H, Ar*H*), 5.45 (dd, *J* = 6.1, 2.2 Hz, 1H, ArC*H*OH), 3.83 (s, 3H, OC*H*$_3$), 2.67 (d, *J* = 2.2 Hz, 1H CC*H*), 2.20 (d, *J* = 6.1 Hz, 1H, O*H*); **^{13}C NMR** (100.6 MHz, $CDCl_3$) δ 160.0 (C), 141.7 (C), 129.9 (CH), 119.0 (CH), 114.4 (CH), 112.1 (CH), 83.5 (C), 74.9 (CH), 64.5 (CH), 55.4 (CH_3); **MS** (ESI+): m/z 145 $[M\text{-}OH^-]^+$; HRMS: found 145.0633 $C_{10}H_9O^+$ requires 145.0648. Compound characterisation data was found to be in agreement with literature values.[S5]

Precursor alcohol for synthesis of 3e: 3-(1-hydroxyprop-2-yn-1-yl)benzonitrile

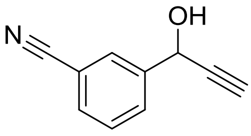

Precursor for (**3e**)

Following the general procedure where addition of Grignard was carried out at -40 °C. The title compound 3-(1-hydroxyprop-2-yn-1-yl)benzonitrile was afforded in 92% yield as a brown oil. **Rf** (1:1 pentane:diethyl ether) 0.39; **IR** (NaCl, film) υ_{max} 3400, 3291, 2233, 2119, 1482, 1434, 1146, 1028, 804, 737, 689 cm^{-1}; **^{1}H**

NMR (500.1 MHz, $CDCl_3$): δ 7.90 – 7.87 (m, 1H, Ar*H*), 7.82 – 7.78 (m, 1H, Ar*H*), 7.64 (dt, *J* = 7.7, 1.5 Hz, 1H, Ar*H*), 7.51 (td, *J* = 7.7, 0.6 Hz, 1H, Ar*H*), 5.51 (dd, *J* = 5.8, 2.2 Hz, 1H, ArC*H*OH), 2.73 (d, *J* = 2.2 Hz, 1H, CC*H*), 2.37 (d, *J* = 5.8 Hz, 1H, O*H*); **^{13}C NMR** (125.8 MHz, $CDCl_3$) δ 141.5 (C), 132.2 (CH), 131.2 (CH), 130.4 (CH), 129.6 (CH), 118.7 (C), 112.9 (C), 82.5 (C), 76.1 (CH), 63.5 (CH); **MS** (ESI+): m/z 158 $[M+H]^+$; HRMS: found 158.0604 $C_{10}H_8NO^+$ requires 158.0600.

Precursor alcohol for synthesis of 3f: 1-(thiophen-3-yl)prop-2-yn-1-ol

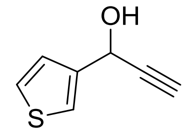

Precursor for (**3f**)

Following the general procedure the title compound 1-(thiophen-3-yl)prop-2-yn-1-ol was afforded in 96% yield as an orange solid. **Rf** (10:1 pentane:diethyl ether) 0.05; **mp** 59-61 °C [lit. 57-59 °C];[S6] **IR** (NaCl, film) υ_{max} 3290, 2119, 1420, 1294, 1150, 1017, 973, 904, 835, 793, 731, 652 cm^{-1}; **^{1}H NMR** (400.2 MHz, $CDCl_3$): δ 7.45 – 7.41 (m, 1H, Ar*H*), 7.33 (dd, *J* = 5.0, 3.0 Hz, 1H, Ar*H*), 7.21 (dd, *J* = 5.0, 1.3 Hz, 1H, Ar*H*), 5.50 (dd, *J* = 6.8, 2.1Hz, 1H, ArC*H*OH), 2.65 (d, *J* = 2.1 Hz, 1H, CC*H*), 2.31 (d, *J* = 6.8 Hz, 1H, O*H*); **^{13}C NMR** (100.6 MHz, $CDCl_3$) δ 141.5 (C), 126.8 (CH), 126.4 (CH), 123.0 (CH), 83.4 (C), 74.2 (CH), 60.5 (CH). Compound characterisation data was found to be in agreement with literature values.[S6]

Precursor alcohol for synthesis of 3g: 1-(naphthalen-1-yl)prop-2-yn-1-ol

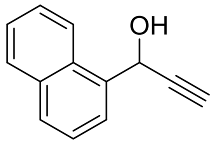

Precursor for (**3g**)

Following the general procedure, then flash column chromatography (17:3 pentane:diethyl ether) of the crude extract afforded the title compound 1-(naphthalen-1-yl)prop-2-yn-1-ol in 89% yield as a pale yellow solid. **Rf** (7:3 pentane:diethyl ether) 0.33; **mp** 62-64 °C [lit. 61-63 °C];[S6a] **IR** (NaCl, film) υ_{max} 3290, 3050, 2118, 1559, 1510, 1005, 936, 802, 780 cm^{-1}; **^{1}H NMR** (400.2 MHz, $CDCl_3$): δ 8.31 – 8.26 (m, 1H, Ar*H*), 7.92 – 7.83 (m, 3H, Ar*H*), 7.612 – 7.45 (m, 3H, Ar*H*), 6.14 (d, *J* = 2.2 Hz, 1H, ArC*H*OH), 2.75 (d, *J* = 2.2 Hz, 1H, CC*H*); **^{13}C NMR** (100.6 MHz, $CDCl_3$) δ 135.1 (C), 134.2 (C), 130.6 (C), 129.7 (CH) 128.9 (CH) 126.7 (CH) 126.1 (CH) 125.4 (CH) 124.8 (CH) 123.9 (CH) 83.3 (C), 75.7 (CH) 62.9 (CH); **MS** (ESI+): m/z 205 $[M+Na^-]^+$; HRMS: found 205.0629 $C_{13}H_{10}NaO^+$ requires 205.0624. Compound characterisation data was found to be in agreement with literature values.[S7]

Precursor alcohol for synthesis of 3h: 1-(naphthalen-2-yl)prop-2-yn-1-ol

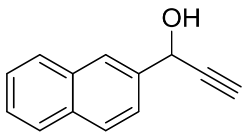

Precursor for (**3h**)

Following the general procedure the title compound 1-(naphthalen-2-yl)prop-2-yn-1-ol was afforded in 99% yield as a brown solid. **Rf** 9:1 pentane:diethyl ether) 0.06; **mp** 53 - 55 °C [lit. 53.8-54.5 °C];[S8] **IR** (NaCl, film) υ_{max} 3289, 3055, 2116, 1600, 1017, 820 cm^{-1}; **^{1}H NMR** (400.2 MHz, $CDCl_3$): δ 8.02 – 7.99 (m, 1H, Ar*H*), 7.90 – 7.82 (m, 3H, Ar*H*), 7.66 (dd, *J* = 8.5, 1.8 Hz, 1H, Ar*H*) 7.54 – 7.48 (m, 2H, Ar*H*), 5.64 (d, *J* = 2.2 Hz, 1H, ArC*H*OH), 2.74 (d, *J* = 2.2 Hz, 1H, CC*H*), 2.38 (br s, 1H, O*H*); **^{13}C NMR** (100.6 MHz, $CDCl_3$) δ 137.4 (C), 133.4 (C), 133.3 (C), 128.8 (CH), 128.4 (CH), 127.8 (CH), 126.6 (CH), 126.5 (CH), 125.6 (CH), 124.6 (CH), 83.6 (C), 75.3 (CH), 64.7(CH); **MS** (ESI+): m/z 165 $[M-OH^-]^+$; HRMS: found 165.0701 $C_{13}H_9^+$ requires 165.0699. ^{1}H NMR data was found to be in agreement with literature values.[S8]

Precursor alcohol for synthesis of 3i: 1-(4-(*tert*-butyl)phenyl)prop-2-yn-1-ol

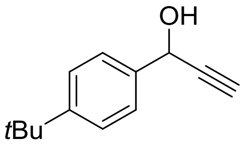

Precursor for (**3i**)

Following the general procedure the title compound 1-(4-(*tert*-butyl)phenyl)prop-2-yn-1-ol was afforded in 95% yield as an orange oil. **Rf** 9:1 pentane:diethyl ether) 0.24; **IR** (NaCl, film) υ_{max} 3292, 2964, 2904, 2869, 2118, 1613, 1511, 1410, 1364, 1269, 1017, 948, 839, cm^{-1}; **^{1}H NMR** (400.2 MHz, $CDCl_3$):7.54 – 7.47 (m, 2H, Ar*H*), 7.45 – 7.39 (m, 2H, Ar*H*), 5.45 (d, *J* = 2.2 Hz, 1H, ArC*H*OH), 2.67 (d, *J* = 2.2 Hz, 1H, CC*H*), 1.99 (s, 9*H*, $C(CH_3)_3$); **^{13}C NMR** (100.6 MHz, $CDCl_3$) δ151.8 (C), 137.3 (C), 126.5 (CH), 125.8 (CH), 83.8 (C), 74.8 (CH), 64.4 (CH), 34.8 (C), 31.4 (CH_3); **MS** (ESI+): m/z 211 $[M+Na]^+$; HRMS: found 211.1099 $C_{13}H_{16}NaO^+$ requires 211.1093. ^{1}H NMR data was found to be in agreement with literature values.[S7]

Synthesis of 2-bromo-1-methyl-1*H*-indole-3-carbaldehyde (1i)

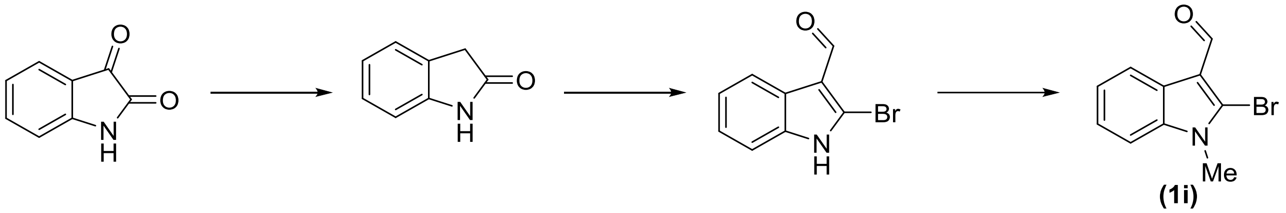

(1i)

Indolin-2-one

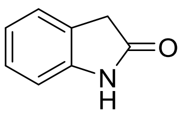

Following the procedure of Lin *et al.*[S9] the title compound indolin-2-one was afforded in 37% yield as a white solid. **Rf** (neat diethyl ether) 0.35; **mp** 116-118 °C [lit. 126-127 °C][S9]; **IR** (NaCl, film) υ_{max} 3140, 3084, 3026, 1736, 1683, 1621, 1476, 1335, 1305, 1234, 1203, 946, 742, 677 cm^{-1}; **^{1}H NMR** (400.2 MHz, $CDCl_3$): δ 8.93 (br s, 1H, N*H*), 7.25 – 7.18 (m, 2H, Ar*H*), 7.05 – 6.98 (m, 1H, Ar*H*), 6.90 (d, *J* = 7.7 Hz, 1H, Ar*H*), 3.55 (s, 2H, ArC*H*$_2$); **^{13}C NMR** (100.6 MHz, $CDCl_3$) δ 178.0 (C), 142.7 (C), 128.1 (CH), 125.4 (C), 124.7 (CH), 122.4 (CH) 109.9 (CH), 36.4 (CH_2); **MS** (ESI+): m/z 156 [M+Na]$^+$; HRMS: found 156.0423 $C_8H_7NNaO^+$ requires 156.0420. Compound characterisation data was found to be in agreement with literature values.[S10]

2-Bromoindole-3-carbaldehyde

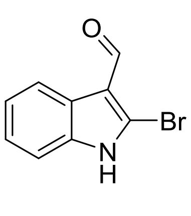

Adapted from a procedure by Gilchrist *et al.*[S11] A solution of *N*,*N*-dimethylformamide (1.19 mL, 15.4 mmol) in dichloromethane (6 mL) was cooled to 0 °C under an atmosphere of argon. A solution of phosphorus oxybromide (3.55 g, 12.4 mmol) in dichloromethane (10 mL) was added dropwise over 10 minutes. This thick cloudy mixture was heated to reflux for 15 minutes before heating was stopped and indolin-2-one (660 mg, 4.96 mmol) was added in portions over 2 minutes. Refluxing was then resumed for 2.5 hours before it was cooled and ice (10 g) was added. The aqueous layer was separated and the organic phase further extracted with water (10 mL). The combined aqueous washings were basified with solid potassium carbonate to form a white precipitate. To complete hydrolysis this was filtered and the precipitate was then added to a mixture of ethanol (20 mL) and 2M aqueous sodium hydroxide solution (8 mL). After stirring for 1 hour the mixture was acidified with 2M hydrochloric acid and filtered to afford the title compound 2-bromoindole-3-carbaldehyde (702 mg, 3.13 mmol) in 63% yield as a pale brown solid. **mp** 212-214 °C decom. [lit. 196-198 °C][S11]; **IR** (ATR) υ_{max} 3103, 2912, 2835, 1639, 1619, 1583, 1492, 1417, 1369, 1342, 1091, 833, 735 cm^{-1}; **^{1}H NMR** (400.2 MHz, $(CD_3)_2SO$): δ 13.08 (br s, 1H, N*H*), 9.90 (s, 1H, C*H*O), 8.07 (dd, *J* = 6.7, 1.4 Hz, 1H, Ar*H*), 7.47 – 7.40 (m, 1H, Ar*H*), 7.32 – 7.18 (m, 2H, Ar*H*); **^{13}C NMR** (100.6 MHz, $(CD_3)_2SO$) δ 184.5 (CH), 136.4 (C), 124.8 (C), 123.8 (CH), 123.3 (C), 122.7 (CH), 119.7 (CH), 114.3 (C), 111.7 (CH); **MS** (ESI+): m/z 226 [M+H]$^+$; HRMS: found 225.9691 $C_9H_7BrNO^+$ requires 225.9685. Compound characterisation data was found to be in agreement with literature values.[S11]

2-Bromo-1-methyl-1*H*-indole-3-carbaldehyde (1i)

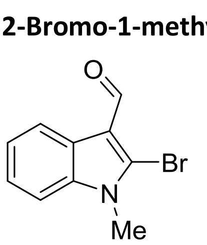

(1i)

2-bromoindole-3-carbaldehyde (680 mg, 3.03 mmol) was dissolved in THF (27 mL) and cooled to 0 °C under an atmosphere of argon. Sodium hydride (60% in mineral oil, 363 mg, 9.09 mmol) was washed with pentane and cooled to 0 °C before the aldehyde solution was added. This was stirred for 30 minutes before iodomethane (0.6 mL, 9.1 mmol) was added. After a further 30 minutes water (10 mL) was added and the reaction mixture extracted with dichloromethane (3 x 30 mL). The combined organic extracts were dried with magnesium sulfate and concentrated *in vacuo* to afford 2-bromo-1-methyl-1*H*-indole-3-carbaldehyde (658 mg, 2.76 mmol) in 91% yield as a pale brown solid. **Rf** (1:1 pentane:diethyl ether) 0.32; **mp** 114-116 °C [lit. 111-112 °C][S11]; **IR** (NaCl, film) υ_{max} 3059, 2923, 1653, 1499, 1469, 1386, 1375, 1036, 797, 744 cm^{-1}; **^{1}H NMR** (400.2 MHz, $CDCl_3$): δ 10.04 (s, 1H, C*H*O), 8.37 – 8.28 (m, 1H, Ar*H*), 7.38 – 7.28 (m, 3H,

Ar*H*), 3.85 (s, 3H, NCH_3); **^{13}C NMR** (100.6 MHz, $CDCl_3$) δ 185.5 (CH), 137.5 (C), 126.5 (C), 125.4 (C), 124.2 (CH), 123.5 (CH), 121.4 (CH), 115.5 (C), 109.8 (CH), 31.9 (CH_3); **MS** (ESI+): m/z 238 $[M+H]^+$; HRMS: found 237.9865 $C_{10}H_9BrNO^+$ requires 237.9862. Compound characterisation data was found to be in agreement with literature values.[S12]

3. General procedure for the one-pot carboannulation to 4

Acetylene **2** (1.62 – 1.70 mmol) was dissolved in tetrahydrofuran (2.3 mL) under argon and cooled to -50 °C. *n*Butyllithium (1.6 M in hexanes, 1.62 – 1.79 mmol) was then added to the solution and the mixture stirred for 20 minutes. Neat bromoaldehyde **1** (1.62 mmol) was added to the reaction mixture. Thin-layer chromatography (9:1 pentane:diethyl ether) was used to confirm consumption of the aldehyde. After 60 minutes additional *n*-butyllithium (1.6 M in hexanes, 1.62 – 1.70 mmol) was added forming yellow, orange or dark coloured solutions. After 15 minutes solid copper(I) bromide dimethyl sulfide (167 mg, 0.81 mmol) was then added under a cushion of argon, normally forming tan suspensions/dark solutions at -50 °C. After a further 1 hour neat propargylic chloride **3** (0.79 mmol) was added to the reaction mixture, which was then allowed to warm slowly to -10 °C over a period of 30 minutes. Once the reaction mixture reached -10 °C it was kept at this temperature for a further 1 hour. The reaction was then quenched with pH = 7 ammonia buffered saturated aqueous ammonium chloride solution (5 mL) and extracted with ethyl acetate (3 × 10 mL). The combined organic extracts were dried with magnesium sulfate and concentrated *in vacuo* to afford the crude product. Flash column chromatography (20:1 pentane:diethyl ether) of the crude extract afforded the title compound **4**. In many cases the 8-ring products could be crystallised from pentane, diethyl ether or methanol.

(6*E*,9*Z*)-8-Phenyl-7-(trimethylsilyl)benzo[8]annulen-5(8*H*)-one (4aaa)

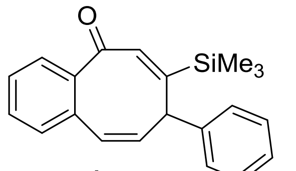

(**4aaa**)

Synthesised from: trimethylsilylacetylene (**2a**) (229 µL, 1.62 mmol), *n*BuLi for acetylene deprotonation (1.0 mL, 1.62 M in hexanes, 1.62 mmol), 2-bromobenzaldehyde (**1a**) (189 µL, 1.62 mmol), *n*BuLi for bromine exchange (1.0 mL, 1.62 M in hexanes, 1.62 mmol), CuBr·SMe_2 (167 mg, 0.81 mmol) and (1-chloroprop-2-yn-1-yl)benzene (**3a**) (119 mg, 0.79 mmol) using the general procedure. Flash column chromatography (20:1 pentane:diethyl ether) afforded the title compound (6*E*,9*Z*)-8-phenyl-7-(trimethylsilyl)benzo[8]annulen-5(8*H*)-one (**4aaa**) (177 mg, 0.55 mmol) in 70% yield as a pale yellow crystalline solid. **Rf** (10:1 pentane:diethyl ether) 0.56; **mp** 104-107 °C; **IR** (NaCl, film) $υ_{max}$ 3753, 3452, 3063, 3030, 2955, 2897, 2099, 1996, 1634, 1496, 1477, 1450, 1277, 1249 cm^{-1}; **^{1}H NMR** (400.2 MHz, $CDCl_3$): δ 7.86 – 7.84 (m, 1H, Ar*H*), 7.43 – 7.35 (m, 2H, Ar*H*), 7.26 – 7.16 (m, 6H, Ar*H*), 6.78 (d, *J* = 10.7 Hz, 1H, CC*H*CH), 6.75 (d, *J* = 1.1 Hz, 1H, OCC*H*), 6.66 (dd, *J* = 10.7 Hz, 9.7 Hz, 1H, CCHC*H*), 4.94 (d, *J* = 9.7 Hz, 1H, C*H*Ph), -0.03 – -0.05 (m, 9H, Si(CH_3)$_3$); **^{13}C NMR** (100.6 MHz, $CDCl_3$) δ 195.3 (C), 165.1 (C), 141.8 (CH), 140.3 (C), 139.8 (C), 136.1 (CH), 135.0 (C), 131.0 (CH), 130.8 (CH), 129.3 (CH), 128.8 (CH), 128.4 (CH), 128.3 (CH), 128.0 (CH), 127.0 (CH), 46.0 (CH), -0.1 (CH_3); **MS** (ESI+): m/z 319 $[M+H]^+$; HRMS: found 319.1504 $C_{21}H_{23}OSi$ requires 319.1513; **CHN** Anal. calcd. for $C_{21}H_{22}OSi$ C: 79.20, H: 6.96%; found C: 79.15, H: 6.84%. Preparation of **4aaa** on a 23.7 mmol scale gave 4.6 g (61%) by direct recrystallization of the crude reaction mass from pentane; X-ray data at: CCDC 1405847. Selected data is presented in Figure S3.

(6*E*,9*Z*)-3-Methoxy-8-phenyl-7-(trimethylsilyl)benzo[8]annulen-5(8*H*)-one (4baa)

Synthesised from: trimethylsilylacetylene (**2a**) (229 µL, 1.62 mmol), *n*BuLi for acetylene deprotonation (1.0 mL, 1.62 M in hexanes, 1.62 mmol), 2-bromo-5-methoxybenzaldehyde (**1b**) (348 mg, 1.62 mmol), *n*BuLi for bromine exchange (1.0 mL, 1.62 M in hexanes, 1.62 mmol), CuBr·SMe_2 (167 mg, 0.81 mmol) and (1-chloroprop-2-yn-1-yl)benzene (**3a**) (119 mg, 0.79 mmol) using the general procedure. Flash column chromatography (20:1 pentane:diethyl ether) afforded the title compound (6*E*,9*Z*)-3-methoxy-8-phenyl-7-

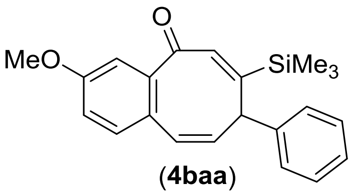

(**4baa**)

(trimethylsilyl)benzo[8]annulen-5(8*H*)-one (**4baa**) (171 mg, 0.49 mmol) in 62% yield as a pale yellow crystalline solid. **Rf** (20:1 pentane:diethyl ether) 0.33; **mp** 91-93 °C; **IR** (NaCl, film) $υ_{max}$ 3451, 3022, 2400, 2095, 1640, 1499, 1336, 1281, 1216 cm^{-1}; ^{1}**H NMR** (400.2 MHz, $CDCl_3$): δ 7.47 – 7.47 (m, 1H, Ar*H*), 7.30 – 7.14 (m, 6H, Ar*H*), 7.04 – 7.01 (m, 1H, Ar*H*), 6.74 (d, *J* = 10.7 Hz, 1H, CC*H*CH), 6.66 (d, *J* = 1.0 Hz, 1H, OCC*H*), 6.61 (dd, *J* = 10.7, 9.6 Hz, 1H, CCHC*H*), 4.84 (d, *J* = 9.6 Hz, 1H, C*H*Ph), 3.87 (s, 3H, CH_3O), -0.05 (s, 9H, $Si(CH_3)_3$); ^{13}**C NMR** (125.8 MHz, $CDCl_3$) δ 195.0 (C), 164.2 (C), 159.2 (C), 140.8 (C), 140.5 (CH), 139.9 (C), 134.8 (CH), 131.8 (CH), 128.8 (CH), 128.7 (C), 128.4 (CH), 128.2 (CH), 127.0 (CH), 119.3 (CH), 113.8 (CH), 55.6 (CH_3), 46.2 (CH), 0.1 (CH_3); **MS** (ESI+): m/z 349 $[M+H]^+$; HRMS: found 349.1623.

(6*E*,9*Z*)-3-Fluoro-8-phenyl-7-(trimethylsilyl)benzo[8]annulen-5(8*H*)-one (4caa)

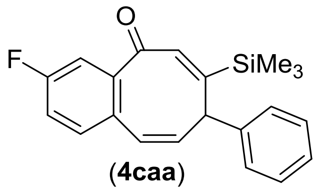

(**4caa**)

Synthesised from: trimethylsilylacetylene (**2a**) (229 µL, 1.62 mmol), *n*BuLi for acetylene deprotonation (1.0 mL, 1.62 M in hexanes, 1.62 mmol), 2-bromo-5-fluorobenzaldehyde (**1c**) (329 mg, 1.62 mmol), *n*BuLi for bromine exchange (1.0 mL, 1.62 M in hexanes, 1.62 mmol), CuBr·SMe_2 (167 mg, 0.81 mmol) and (1-chloroprop-2-yn-1-yl)benzene (**3a**) (119 mg, 0.79 mmol) using the general procedure. Flash column chromatography (25:1 pentane:diethyl ether) afforded the title compound (6*E*,9*Z*)-3-fluoro-8-phenyl-7-(trimethylsilyl)benzo[8]annulen-5(8*H*)-one (**4caa**) (146 mg, 0.435 mmol) in 55% yield as a yellow crystalline solid. **Rf** (20:1 pentane:diethyl ether) 0.56; **mp** 90-92 °C; **IR** (NaCl, film) $υ_{max}$ 3064, 3032, 2957, 2897, 1601, 1568, 1488, 1451, 1408, 1384, 1328, 1307, 1270, 1250, 1234, 1201, 1121, 1087, 1071, 1034 cm^{-1}; ^{1}**H NMR** (500.1 MHz, $CDCl_3$): δ 7.58 – 7.51 (m, 1H, Ar*H*), 7.26 – 7.21 (m, 2H Ar*H*), 7.21 – 7.14 (m, 4H Ar*H*), 7.14 – 7.06 (m, 1H Ar*H*), 6.74 (d, *J* = 10.7 Hz, 1H, CC*H*CH), 6.72 (d, *J* = 1.1 Hz, 1H, OCC*H*), 6.64 (dd, *J* = 10.7, 9.7 Hz, 1H, CCHC*H*), 4.89 (d, *J* = 9.7 Hz, 1H, C*H*Ph), 0.00 – -0.07 (m, 9H, $Si(CH_3)_3$); ^{13}**C NMR** (125.8 MHz, $CDCl_3$) δ 193.6 (d, *J* = 1.4 Hz, C), 165.8 (s, C), 162.3 (d, *J* = 248 Hz, C), 141.8 (d, *J* = 6.4 Hz, C), 140.9 (s, CH), 139.5 (s, C), 136.1 (s, CH), 131.6 (d, *J* = 7.6 Hz, CH), 131.4 (d, *J* = 3.2 Hz, C), 128.5 (s, CH), 128.2 (s, CH), 128.0 (s, CH), 127.1 (s, CH), 118.6 (d, *J* = 22.0 Hz, CH), 117.0 (d, *J* = 22.9 Hz, CH), 46.0 (s, CH), -0.2 (s, CH_3); **MS** (ESI+): m/z 337 $[M+H]^+$; HRMS: found 337.1399 $C_{21}H_{22}FOSi$ requires 337.1418.

(6*E*,9*Z*)-4-Fluoro-8-phenyl-7-(trimethylsilyl)benzo[8]annulen-5(8*H*)-one (4daa)

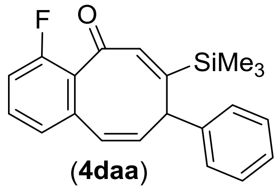

(**4daa**)

Synthesised from: trimethylsilylacetylene (**2a**) (229 µL, 1.62 mmol), *n*BuLi for acetylene deprotonation (1.0 mL, 1.62 M in hexanes, 1.62 mmol), 2-bromo-5-fluorobenzaldehyde (**1c**) (329 mg, 1.62 mmol), *n*BuLi for bromine exchange (1.0 mL, 1.62 M in hexanes, 1.62 mmol), CuBr·SMe_2 (167 mg, 0.81 mmol) and (1-chloroprop-2-yn-1-yl)benzene (**3a**) (119 mg, 0.79 mmol) using the general procedure. Flash column chromatography (20:1 pentane:diethyl ether) afforded the title compound (6*E*,9*Z*)-4-fluoro-8-phenyl-7-(trimethylsilyl)benzo[8]annulen-5(8*H*)-one (**4daa**) (72 mg, 0.214 mmol) in 27% yield as a yellow crystalline solid. **Rf** (20:1 pentane:diethyl ether) 0.23; **mp** 104-106 °C; **IR** (NaCl, film) $υ_{max}$ 3062, 3028, 2956, 2898, 1633, 1607, 1571, 1494, 1448, 1251, 1016, 904, 841 cm^{-1}; ^{1}**H NMR** (400.2 MHz, $CDCl_3$): δ 7.16 – 7.02 (m, 6H, Ar*H*), 6.96 – 6.91 (m, 1H Ar*H*), 6.90 (d, *J* = 1.1 Hz, 1H, OCC*H*), 6.73 (d, *J* = 10.5 Hz, 1H, CC*H*CH), 6.69 (d, *J* = 7.6 Hz, 1H, Ar*H*) 6.43 (dd, *J* = 10.5, 9.7 Hz, 1H, CCHC*H*), 4.90 (d, *J* = 9.7 Hz, 1H, C*H*Ph), 0.01 (s, 9H, $Si(CH_3)_3$); ^{13}**C NMR** (100.05 MHz, $CDCl_3$) δ 192.7 (s, C), 165.1 (s, C), 159.2 (d, *J* = 249.9 Hz, C), 142.5 (s, CH), 139.7 (s, C), 137.1 (s, CH), 135.8 (d, *J* = 3.0 Hz, C), 130.0 (d, *J* = 8.4 Hz, CH), 129.3 (d, *J* = 16.1 Hz, C), 128.3 (s, CH), 128.1 (s, CH), 127.5 (d, *J* = 2.3, CH), 126.6 (s, CH), 122.6 (d, *J* = 3.1, CH), 114.7 (d, *J* = 21.5, CH), 46.2 (s, CH), -0.9 (s, CH_3); **MS** (ESI+): m/z 359 $[M+Na]^+$; HRMS: found 359.1236 $C_{21}H_{21}FNaOSi$ requires 359.1238.

(6*E*,9*Z*)-2,3-Dimethoxy-8-phenyl-7-(trimethylsilyl)benzo[8]annulen-5(8*H*)-one (4eaa)

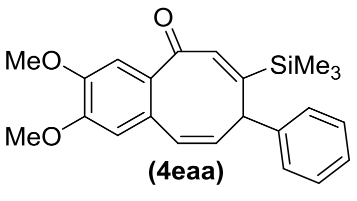

(4eaa)

Synthesised from: trimethylsilylacetylene (**2a**) (229 µL, 1.62 mmol), *n*BuLi for acetylene deprotonation (1.0 mL, 1.62 M in hexanes, 1.62 mmol), 6-bromoveratraldehyde (**1e**) (397 mg, 1.62 mmol), *n*BuLi for bromine exchange (1.0 mL, 1.62 M in hexanes, 1.62 mmol), CuBr·SMe_2 (167 mg, 0.81 mmol) and (1-chloroprop-2-yn-1-yl)benzene (**3a**) (119 mg, 0.79 mmol) using the general procedure. Flash column chromatography (5:2 pentane:diethyl ether) afforded the title compound (6*E*,9*Z*)-2,3-dimethoxy-8-phenyl-7-(trimethylsilyl)benzo[8]annulen-5(8*H*)-one (**4eaa**) (110mg, 0.291 mmol) in 37% yield as a colourless solid. **Rf** (5:2 pentane:diethyl ether) 0.27; **mp** 145-147 °C; **IR** (NaCl, film) υ_{max} 3006, 2957, 1583, 1515, 1463, 1368, 1260, 1225, 1090, 841 cm^{-1}; **^{1}H NMR** (400.2 MHz, $CDCl_3$): 7.68 (s, 1H, Ar*H*), 7.38 – 7.16 (m, 5H, Ar*H*), 6.75 – 6.64 (m, 3H, Ar*H*, CCHC*H*, CC*H*CH), 6.61 (d, *J* = 0.8 Hz, 1H, OCC*H*), 4.82 (d, *J* = 7.2 Hz, 1H, C*H*Ph), 3.97 (s, 3H, OCH_3), 3.93 (s, 3H, OCH_3), -0.06 (s, 9H, Si(CH_3)$_3$); **^{13}C NMR** (100.05 MHz, $CDCl_3$) δ 193.6 (C), 162.6 (C), 151.8 (C), 148.8 (C), 140.3 (CH), 139.8 (C), 135.5 (CH), 132.7 (C), 131.0 (C), 129.0 (CH), 128.5(CH), 128.1(CH), 127.02 (CH), 113.2 (CH), 112.1 (CH), 56.2 (2 × CH_3), 46.2 (CH), 0.29 (CH_3) **MS** (ESI+): m/z 401 $[M+Na]^+$; HRMS: found 401.1528 $C_{23}H_{26}NaO_3Si$ requires 401.1543.

(8*E*,11*Z*)-10-Phenyl-9-(trimethylsilyl)cycloocta[*a*]naphthalen-7(10*H*)-one (4faa)

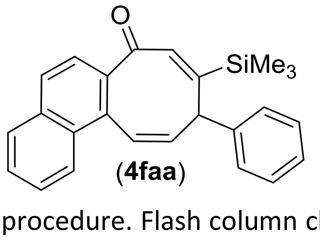

(**4faa**)

Synthesised from: trimethylsilylacetylene (**2a**) (235 µL, 1.70 mmol), *n*BuLi for acetylene deprotonation (1.2 mL, 1.50 M in hexanes, 1.79 mmol), 1-bromo-2-naphthaldehyde (**1f**) (381 mg, 1.62 mmol), *n*BuLi for bromine exchange (1.1 mL, 1.50 M in hexanes, 1.65 mmol), CuBr·SMe_2 (167 mg, 0.81 mmol) and (1-chloroprop-2-yn-1-yl)benzene (**3a**) (119 mg, 0.79 mmol) using the general procedure. Flash column chromatography (15:1 to 5:1 pentane:diethyl ether gradient elution) afforded the title compound (8*E*,11*Z*)-10-phenyl-9-(trimethylsilyl)cycloocta[*a*]naphthalen-7(10*H*)-one (**4faa**) (77 mg, 0.21 mmol) in 26% yield as colourless solid. **Rf** (5:1 pentane:diethyl ether) 0.42; **mp** 142-144 °C; **IR** (NaCl, film) υ_{max} 3025, 2954, 1694, 1614, 1458, 1274, 1258, 1075, 890, 840 cm^{-1}; **^{1}H NMR** (400.1 MHz, $CDCl_3$): δ 8.30 – 8.24 (m, 1H, Ar*H*), 7.96 (dd, *J* = 7.2, 1.1 Hz, 1H, Ar*H*), 7.93 – 7.86 (m, 2H, Ar*H*), 7.71 – 7.56 (m, 2H, Ar*H*), 7.31 – 7.19 (m, 5H, Ar*H*), 7.18 (d, *J* = 1.7 Hz, 1H, OCC*H*), 6.68 (dd, *J* = 15.9, 1.1 Hz, 1H, CC*H*CH), 6.26 (dd, *J* = 15.9, 8.4 Hz, 1H, CCHC*H*), 5.18 (ddd, *J* = 8.4, 1.1, 1.1 Hz, 1H, C*H*Ph), 0.28 (s, 9H, Si(CH_3)$_3$); **^{13}C NMR** (125.8 MHz, $CDCl_3$) δ 192.2 (C), 153.1 (C), 152.6 (C), 137.5 (C), 137.0 (CH), 136.7 (C), 135.0 (C), 132.4 (CH), 130.2 (CH), 130.1 (C), 129.8 (CH), 129.5 (CH), 129.2 (CH), 128.8 (CH), 127.8 (CH), 127.3 (CH), 126.4 (CH), 125.5 (CH), 120.4 (CH), 45.9 (CH), -0.3 (CH_3); **MS** (ESI+): m/z 369 $[M+H]^+$; HRMS: found 369.1667 $C_{25}H_{25}OSi^+$ requires 369.1669.

(6*E*,9*Z*)-2-Methyl-8-phenyl-7-(trimethylsilyl)benzo[8]annulen-5(8*H*)-one (4gaa)

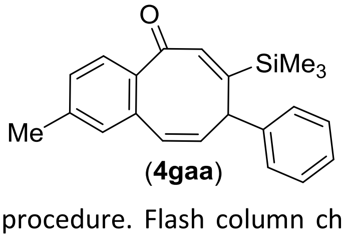

(**4gaa**)

Synthesised from: trimethylsilylacetylene (**2a**) (235 µL, 1.70 mmol), *n*BuLi for acetylene deprotonation (1.1 mL, 1.60 M in hexanes, 1.76 mmol), 2-bromo-4-methylbenzaldehyde (**1g**) (322 mg, 1.62 mmol), *n*BuLi for bromine exchange (1.1 mL, 1.60 M in hexanes, 1.76 mmol), CuBr·SMe_2 (167 mg, 0.81 mmol) and (1-chloroprop-2-yn-1-yl)benzene (**3a**) (119 mg, 0.79 mmol) using the general procedure. Flash column chromatography (neat pentane to 20:1 pentane:diethyl ether gradient elution) afforded the title compound (6*E*,9*Z*)-2-methyl-8-phenyl-7-(trimethylsilyl)benzo[8]annulen-5(8*H*)-one (**4gaa**) (162 mg, 0.49 mmol) in 62% yield as colourless solid. **Rf** (9:1 pentane:diethyl ether) 0.49; **mp** 125-127 °C; **IR** (NaCl, film) υ_{max} 3028, 2954, 2896, 1599, 1496, 1278, 1250, 913, 902, 840, 753, 699 cm^{-1}; **^{1}H NMR** (400.1 MHz, $CDCl_3$): δ 7.87 (d, *J* = 8.2 Hz, 1H, Ar*H*), 7.30 – 7.15 (m, 6H, Ar*H*), 7.05 – 7.00 (m, 1H, Ar*H*), 6.73 (d, *J* = 10.8 Hz, 1H, CC*H*CH), 6.70 (d, *J* = 1.2 Hz, 1H, OCC*H*), 6.67 (dd, *J* = 10.8, 9.2 Hz, 1H, CCHC*H*), 4.94 (d, *J* = 9.2 Hz, 1H, C*H*Ph), 2.36 (s, 3H, ArCH_3), -0.06 (s, 9H, Si(CH_3)$_3$); **^{13}C NMR** (125.8 MHz, $CDCl_3$) δ 194.8 (C), 164.4 (C),

141.7 (C), 141.6 (CH), 139.9 (C), 137.6 (C), 136.0 (CH), 135.4 (C), 131.3 (CH), 130.1 (CH), 129.0 (CH), 129.0 (CH), 128.4 (CH), 128.2 (CH), 127.0 (CH), 46.0 (CH), 21.4 (CH_3), 0.1 (CH_3); **MS** (ESI+): m/z 355 $[M+Na]^+$; HRMS: found 355.1486 $C_{22}H_{24}NaOSi^+$ requires 355.1489; **CHN** Anal. calcd. for $C_{22}H_{24}OSi$ C: 79.47, H: 7.29%; found C: 79.37, H: 7.31%.

(4*Z*,7*E*)-6-Phenyl-7-(trimethylsilyl)cycloocta[*b*]thiophen-9(6*H*)-one (4haa)

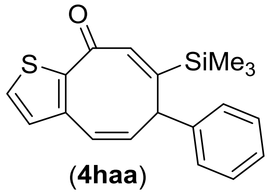

(**4haa**)

Synthesised from: trimethylsilylacetylene (**2a**) (229 µL, 1.62 mmol), *n*BuLi for acetylene deprotonation (1.0 mL, 1.62 M in hexanes, 1.62 mmol), 3-bromothiophene-2-carboxaldehyde (**1h**) (316 mg, 1.62 mmol), *n*BuLi for bromine exchange (1.0 mL, 1.62 M in hexanes, 1.62 mmol), $CuBr{\cdot}SMe_2$ (167 mg, 0.81 mmol) and (1-chloroprop-2-yn-1-yl)benzene (**3a**) (119 mg, 0.79 mmol) using the general procedure. Flash column chromatography (20:1 to 9:1 pentane:diethyl ether gradient) afforded the title compound (4Z,7*E*)-6-phenyl-7-(trimethylsilyl)cycloocta[*b*]thiophen-9(6*H*)-one (**4haa**) (185 mg, 0.570 mmol) in 72% yield as a pale yellow crystalline solid. **Rf** (15:1 pentane:diethyl ether) 0.38; **mp** 112-115 °C; **IR** (NaCl, film) υ_{max} 3089, 3063, 3013, 2955, 2897, 1597, 1567, 1513, 1497, 1451, 1409, 1355, 1280, 1249, 1216, 1088, 1069, 1031, 1009 cm^{-1}; **^{1}H NMR** (400.1 MHz, $CDCl_3$): δ 7.64 (d, *J* = 5.2 Hz, 1H, C*H*SC), 7.37 – 7.19 (m, 5H, Ar*H*), 7.12 (d, *J* = 5.2 Hz, 1H, C*H*CHS), 6.92 (d, *J* = 10.7 Hz, 1H, CC*H*CH), 6.79 (dd, *J* = 10.7, 9.2 Hz, 1H, CCHC*H*), 6.47 (s, 1H, OCC*H*), 4.49 (d, *J* = 9.2 Hz, 1H, C*H*Ph), -0.08 – -0.09 (m, 9H, Si(C*H*$_3$)$_3$); **^{13}C NMR** (125.8 MHz, $CDCl_3$) δ 188.0 (C), 159.7 (C), 145.8 (C), 139.9 (C), 139.4 (C), 136.3 (CH), 136.2 (CH), 133.6 (CH), 132.0 (CH), 128.6 (CH), 127.6 (CH), 127.2 (CH), 125.4 (CH), 47.1 (CH), 0.4 (CH_3); **MS** (ESI+): m/z 325 $[M+H]^+$; HRMS: found 325.1061 $C_{19}H_{21}OSSi$ requires 325.1077; X-ray data at: CCDC 1405851. Selected data in Figure S4.

(8*E*,11*Z*)-10-Phenyl-9-(trimethylsilyl)cycloocta[*a*]naphthalen-7(10*H*)-one (4iaa)

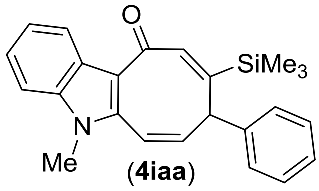

(**4iaa**)

Synthesised from: trimethylsilylacetylene (**2a**) (203 µL, 1.44 mmol), *n*BuLi for acetylene deprotonation (0.9 mL, 1.60 M in hexanes, 1.44 mmol), 2-bromo-1-methyl-1H-indole-3-carbaldehyde (**1i**) (294 mg, 1.23 mmol), *n*BuLi for bromine exchange (0.8 mL, 1.60 M in hexanes, 1.28 mmol), $CuBr{\cdot}SMe_2$ (127 mg, 0.62 mmol) and (1-chloroprop-2-yn-1-yl)benzene (**3a**) (90 mg, 0.60 mmol) using the general procedure. Flash column chromatography (17:3 to 7:3 pentane:diethyl ether gradient elution) afforded the title compound (8*E*,11*Z*)-10-phenyl-9-(trimethylsilyl)cycloocta[*a*]naphthalen-7(10*H*)-one (**4iaa**) (45 mg, 0.16 mmol) in 20% yield as colourless solid. **Rf** (1:1 pentane:diethyl ether) 0.41; **mp** 213-215 °C decom. °C; **IR** (NaCl, film) υ_{max} 3055, 2953, 2896, 1583, 1565, 1496, 1465, 1417, 1375, 1334, 1248, 1054, 840, 749, 700 cm^{-1}; **^{1}H NMR** (400.1 MHz, $CDCl_3$): δ 8.93 – 8.82 (m, 1H, Ar*H*), 7.46 – 7.18 (m, 8H, Ar*H*), 7.10 (dd, *J* = 11.0, 9.7 Hz, 1H, CCHC*H*), 6.84 (d, *J* = 11.0 Hz, 1H, CC*H*CH), 6.55 (s, 1H, OCC*H*), 4.76 (d, *J* = 9.7 Hz, 1H, C*H*Ph), 3.81 (s, 3H, NC*H*$_3$), -0.07 (s, 9H, Si(C*H*$_3$)$_3$); **^{13}C NMR** (125.8 MHz, $CDCl_3$) δ 189.7 (C), 155.7 (C), 141.1 (C), 140.2 (CH), 140.1 (CH), 139.6 (C), 137.5 (C), 128.6 (CH), 127.8 (CH), 127.6 (C), 127.1 (CH), 124.9 (CH), 124.7 (CH), 123.2 (CH), 119.9 (C), 117.5 (CH), 109.1 (CH), 46.8 (CH), 30.8 (CH_3), 0.5 (CH_3); **MS** (ESI+): m/z 372 $[M+H]^+$; HRMS: found 372.1781 $C_{24}H_{26}NOSi^+$ requires 372.1778. Compound **8** was also isolated in 22% yield from this reaction (see later).

(6*E*,9*Z*)-8-Phenyl-7-(trimethylsilyl)cycloocta[4,5]benzo[1,2-*d*][1,3]dioxol-5(8*H*)-one (4jaa)

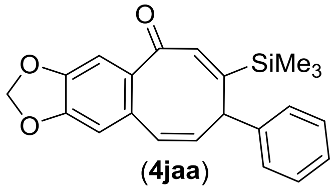

(**4jaa**)

Synthesised from: trimethylsilylacetylene (**2a**) (235 µL, 1.70 mmol), *n*BuLi for acetylene deprotonation (1.1 mL, 1.60 M in hexanes, 1.76 mmol), 6-bromo-1,3-benzodioxole-5-carboxaldehyde (**1j**) (371 mg, 1.62 mmol), *n*BuLi for bromine exchange (1.1 mL, 1.60 M in hexanes, 1.76 mmol), $CuBr{\cdot}SMe_2$ (167 mg, 0.81 mmol) and (1-chloroprop-2-yn-1-yl)benzene (**3a**) (119 mg, 0.79 mmol) using the general procedure. Flash column chromatography (19:1 pentane:diethyl ether) afforded

the title compound (6*E*,9*Z*)-8-phenyl-7-(trimethylsilyl)cycloocta[4,5]benzo[1,2-*d*][1,3]dioxol-5(8*H*)-one (**4haa**) (150 mg, 0.41 mmol) in 52% yield as colourless solid. **Rf** (9:1 pentane:diethyl ether) 0.24; **mp** 155-157 ^{o}C; **IR** (NaCl, film) υ_{max} 3028, 2954, 2898, 1590, 1504, 1483, 1378, 1248, 1037, 839, 754, 700 cm^{-1}; **^{1}H NMR** (400.1 MHz, $CDCl_3$): δ 7.48 (s, 1H, Ar*H*), 7.30 – 7.24 (m, 2H, Ar*H*), 7.23 – 7.17 (m, 3H, Ar*H*), 6.66 (d, *J* = 1.1 Hz, 1H, OCC*H*), 6.64 – 6.58 (m, 3H, CCHC*H*, CC*H*CH, Ar*H*), 6.02 (d, *J* = 1.3 Hz, 1H, OCH_aH_bO), 6.00 (d, *J* = 1.3 Hz, 1H, OCH_bH_aO), 4.93 – 4.84 (m, 1H, C*H*Ph), -0.06 (s, 9H, Si(CH_3)$_3$); **^{13}C NMR** (125.8 MHz, $CDCl_3$) δ 193.0 (C), 163.9 (C), 150.4 (C), 148.0 (C), 141.0 (CH), 139.8 (C), 135.6 (CH), 134.9 (C), 132.3 (C), 128.5 (2 x CH), 128.2 (CH), 127.0 (CH), 110.3 (CH), 108.6 (CH), 102.0 (CH_2), 46.1 (CH), 0.1 (CH_3) ; **MS** (ESI+): m/z 363 [M+H]$^+$; HRMS: found 363.1416 $C_{22}H_{23}O_3Si^+$ requires 363.1411. **CHN** Anal. calcd. for $C_{22}H_{22}O_3Si$ C: 72.89, H: 6.12%; found C: 72.75, H: 6.20%. X-ray data at: CCDC 1405854. Selected data presented in Figure S5.

(6*E*,9*Z*)-7,8-Diphenylbenzo[8]annulen-5(8*H*)-one (4aba)

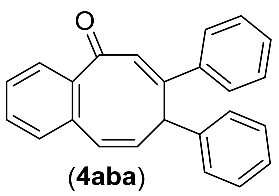

Synthesised from: phenylacetylene (**2b**) (187 µL, 1.70 mmol), *n*BuLi for acetylene deprotonation (1.2 mL, 1.52 M in hexanes, 1.82 mmol), 2-bromobenzaldehyde (**1a**) (189 µL, 1.62 mmol) *n*BuLi for bromine exchange (1.1 mL, 1.52 M in hexanes, 1.82 mmol), CuBr·SMe_2 (167 mg, 0.81 mmol) and (1-chloroprop-2-yn-1-yl)benzene (**3a**) (119 mg, 0.79 mmol) using the general procedure. Flash column chromatography (gradient elution: 19:1 to 9:1 pentane:diethyl ether) of the crude extract afforded the title compound (6*E*,9*Z*)-7,8-diphenylbenzo[8]annulen-5(8*H*)-one (**4aba**) (128 mg, 0.40 mmol) in 50% yield as a colourless crystalline solid. **Rf** (9:1 pentane:diethyl ether) 0.24; **mp** 138-140 ^{o}C; **IR** (NaCl, film) υ_{max} 3059, 3028, 1607, 1587, 1494, 1443, 1291, 1249, 1117, 1031, 787, 697 cm^{-1}; **^{1}H NMR** (400.2 MHz, $CDCl_3$): δ 7.82 – 7.74 (m, 1H, Ar*H*), 7.45 – 7.34 (m, 2H, Ar*H*), 7.25 – 7.14 (m, 6H, Ar*H*), 7.12 – 6.95 (m, 5H, Ar*H*), 6.91 (d, *J* = 10.4 Hz, 1H, CC*H*CH), 6.82 – 6.74 (m, 2H, OCC*H*, CCHC*H*), 5.25 (d, *J* = 9.6 Hz, 1H, C*H*Ph); **^{13}C NMR** (100.05 MHz, $CDCl_3$) δ 194.9 (C), 161.8 (C), 141.2 (C), 139.9 (C), 139.0 (C),137.0 (CH), 134.9 (CH), 134.5 (C), 130.6 (CH), 130.6 (CH), 129.5 (CH), 128.5 (CH), 128.4 (CH), 128.3 (CH), 128.2 (2 × CH), 128.1 (CH), 128.0 (CH), 126.4 (CH), 45.9 (CH); **MS** (ESI+): m/z 323 [M+H]$^+$; HRMS: found 323.1433 $C_{24}H_{19}O$ requires 323.1430; **CHN** Anal. calcd. for $C_{24}H_{18}O$ C: 89.41, H: 5.63%; found C: 89.09, H: 5.63%.

(6*E*,9*Z*)-7-(*tert*-Butyl)-8-phenylbenzo[8]annulen-5(8*H*)-one (4aca)

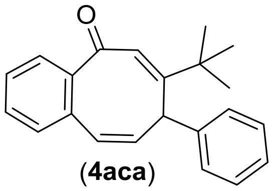

Synthesised from: 3,3-dimethyl-1-butyne (**2c**) (209 µL, 1.70 mmol), *n*BuLi for acetylene deprotonation (1.2 mL, 1.52 M in hexanes, 1.82 mmol), 2-bromobenzaldehyde (**1a**) (189 µL, 1.62 mmol) *n*BuLi for bromine exchange (1.1 mL, 1.52 M in hexanes, 1.67 mmol), CuBr·SMe_2 (167 mg, 0.81 mmol) and (1-chloroprop-2-yn-1-yl)benzene (**3a**) (119 mg, 0.79 mmol) using the general procedure. Flash column chromatography (20:1 pentane:diethyl ether) of the crude extract afforded the title compound (6*E*,9*Z*)-7-(*tert*-butyl)-8-phenylbenzo[8]annulen-5(8*H*)-one (**4aca**) (144 mg, 0.48 mmol) in 61% yield as a colourless crystalline solid. **Rf** (10:1 pentane:diethyl ether) 0.25; **mp** 100 - 102 ^{o}C; **IR** (NaCl, film) υ_{max} 3059, 3023, 2966, 1623, 1608, 1592, 1298, 1237, 778, 736 cm^{-1}; **^{1}H NMR** (500.1 MHz, $CDCl_3$): δ 7.18 – 7.10 (m, 1H, Ar*H*), 7.04 – 6.95 (m, 2H, Ar*H*), 6.92 – 6.78 (m, 5H, Ar*H*), 6.77 – 6.71 (m, 3H, CC*H*CH, OCC*H*, Ar*H*) 6.42 (dd, *J* = 10.9 Hz, 9.1 Hz, 1H, CCHC*H*), 4.76 (d, *J* = 9.1 Hz, 1H, C*H*Ph), 1.25 (s, 9H, C(CH_3)$_3$); **^{13}C NMR** (125.03 MHz, $CDCl_3$) δ 197.5 (C), 168.8 (C), 140.9 (C), 139.2 (C), 137.5 (CH), 134.0 (C), 130.5 (CH), 130.2 (CH), 129.3 (CH), 128.9 (CH), 127.6 (CH), 127.4 (CH), 127.4 (CH), 127.1 (CH), 125.6 (CH), 45.3 (CH), 39.1 (C), 29.0 (CH_3); **MS** (ESI+): m/z 325 [M+Na]$^+$; HRMS: found 325.1556 $C_{22}H_{22}NaO$ requires 323.1563; **CHN** Anal. calcd. for $C_{22}H_{22}O$ C: 87.38, H: 7.33%; found C: 87.27, H: 7.27%.

(6*E*,9*Z*)-7-(Cyclohex-1-en-1-yl)-8-phenylbenzo[8]annulen-5(8*H*)-one (4ada)

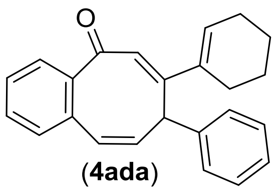

(**4ada**)

Synthesised from: 1-ethynylcyclohexene (**2d**) (199 µL, 1.70 mmol), *n*BuLi for acetylene deprotonation (1.2 mL, 1.52 M in hexanes, 1.82 mmol), 2-bromobenzaldehyde (**1a**) (189 µL, 1.62 mmol) *n*BuLi for bromine exchange (1.1 mL, 1.52 M in hexanes, 1.67 mmol), CuBr·SMe$_2$ (167 mg, 0.81 mmol) and (1-chloroprop-2-yn-1-yl)benzene (**3a**) (119 mg, 0.79 mmol) using the general procedure. Flash column chromatography (9:1 pentane:diethyl ether) of the crude extract afforded the title compound (6*E*,9*Z*)-7-(cyclohex-1-en-1-yl)-8-phenylbenzo[8]annulen-5(8*H*)-one (**4ada**) (147 mg, 0.45 mmol) in 57% yield as a yellow crystalline solid. **Rf** (9:1 pentane:diethyl ether) 0.23; **mp** 90 - 92 °C; **IR** (NaCl, film) υ_{max} 3059, 3027, 2929, 1604, 1587, 1496, 1448, 1285, 1116, 781, 744, 699 cm^{-1}; **^{1}H NMR** (400.2 MHz, CDCl$_3$): δ 7.77 – 7.67 (m, 1H, Ar*H*), 7.41 – 7.30 (m, 2H, Ar*H*), 7.23 – 7.07 (m, 6H, Ar*H*), 6.83 (d, *J* = 10.5 Hz, 1H, CC*H*CH), 6.58 (dd, *J* = 10.5 Hz, 9.7 Hz, 1H, CCHC*H*), 6.58 (s, 1H, OCC*H*), 5.92 (m, 1H, CC*H*CH$_2$), 4.99 (d, *J* = 9.7 Hz, 1H, C*H*Ph), 2.12 – 1.72 (m, 4H, CCHC*H$_2$*, CC*H$_2$*), 1.54 – 1.18 (m, 4H, 2 × CH$_2$C*H$_2$*CH$_2$); **^{13}C NMR** (100.05 MHz, CDCl$_3$) δ 195.3 (C), 164.7 (C), 141.4 (C), 139.5 (C), 138.0 (C), 136.8 (CH), 134.4 (C), 131.6 (CH), 130.6 (CH), 130.3 (CH), 130.0 (CH), 129.3 (CH), 128.3 (CH), 128.0 (CH), 128.0 (CH), 127.9 (CH), 126.4 (CH), 44.4 (CH), 27.8 (CH$_2$), 25.8 (CH$_2$), 22.4 (CH$_2$), 21.7 (CH$_2$); **MS** (ESI+): m/z 327 [M+H]$^+$; HRMS: found 327.1745 $C_{24}H_{23}O$ requires 327.1743; **CHN** Anal. calcd. for $C_{24}H_{22}O$ C: 88.31, H: 6.79%; found C: 88.12, H: 6.84%.

(6*E*,9*Z*)-8-Phenyl-7-(prop-1-en-2-yl)benzo[8]annulen-5(8*H*)-one (4aea)

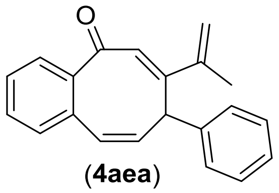

(**4aea**)

Synthesised from: 2-methyl-1-buten-3-yne (**2e**) (162 µL, 1.70 mmol), *n*BuLi for acetylene deprotonation (1.2 mL, 1.48 M in hexanes, 1.78 mmol), 2-bromobenzaldehyde (**1a**) (189 µL, 1.62 mmol) *n*BuLi for bromine exchange (1.1 mL, 1.48 M in hexanes, 1.63 mmol), CuBr·SMe$_2$ (167 mg, 0.81 mmol) and (1-chloroprop-2-yn-1-yl)benzene (**3a**) (119 mg, 0.79 mmol) using the general procedure. Flash column chromatography (gradient elution: 19:1 to 9:1 pentane:diethyl ether) of the crude extract afforded the title compound (6*E*,9*Z*)-8-phenyl-7-(prop-1-en-2-yl)benzo[8]annulen-5(8*H*)-one (**4aea**) (113 mg, 0.39 mmol) in 50% yield as a colourless crystalline solid. **Rf** (9:1 pentane:diethyl ether) 0.32; **mp** 85 - 87 °C; **IR** (NaCl, film) υ_{max} 3028, 2921, 1608, 1588, 1496, 1445, 1288, 1251, 1122, 1023, 899, 786 cm^{-1}; **^{1}H NMR** (400.2 MHz, CDCl$_3$): δ 7.80 – 7.72 (m, 1H, Ar*H*), 7.43 – 7.31 (m, 2H, Ar*H*), 7.24 – 7.08 (m, 6H, Ar*H*), 6.85 (d, *J* = 10.5 Hz, 1H, CC*H*CH), 6.64 (dd, *J* = 10.5 Hz, 9.4 Hz, 1H, CCHC*H*), 6.64 (s, 1H, OCC*H*), 5.11 (s, 1H, CC*H*$_a$H$_b$), 5.03 – 5.01 (m, 1H, CCH$_a$C*H*$_b$), 5.02 (d, *J* = 9.4 Hz, 1H, CCHC*H*), 1.69 (s, 3H, CC*H$_3$*); **^{13}C NMR** (100.05 MHz, CDCl$_3$) δ 195.3 (C), 163.0 (C), 144.3 (C), 141.0 (C), 139.2 (C), 136.6 (CH), 134.5 (C), 132.5 (CH), 130.7 (CH), 130.6 (CH), 129.7 (CH), 128.7 (CH), 128.2 (CH), 128.1 (CH), 127.8 (CH), 126.6 (CH), 118.1 (CH$_2$), 44.3 (CH), 22.1 (CH$_3$); **MS** (ESI+): m/z 309 [M+Na]$^+$; HRMS: found 309.1258 $C_{21}H_{18}NaO^+$ requires 309.1250.

(6*E*,9*Z*)-7-(3-Chlorophenyl)-8-phenylbenzo[8]annulen-5(8*H*)-one (4afa)

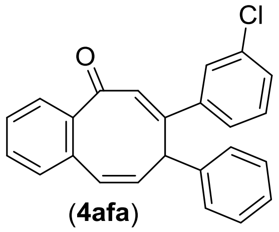

(**4afa**)

Synthesised from: 3-chloro-1-ethynylbenzene (**2f**) (209 µL, 1.70 mmol), *n*BuLi for acetylene deprotonation (1.1 mL, 1.60 M in hexanes, 1.76 mmol), 2-bromobenzaldehyde (**1a**) (189 µL, 1.62 mmol) *n*BuLi for bromine exchange (1.1 mL, 1.60 M in hexanes, 1.76 mmol), CuBr·SMe$_2$ (167 mg, 0.81 mmol) and (1-chloroprop-2-yn-1-yl)benzene (**3a**) (119 mg, 0.79 mmol) using the general procedure. Flash column chromatography (gradient elution: 19:1 to 9:1 pentane:diethyl ether) of the crude extract afforded the title compound (6*E*,9*Z*)-7-(3-chlorophenyl)-8-phenylbenzo[8]annulen-5(8*H*)-one (**4afa**) (102 mg, 0.29 mmol) in 37% yield as a colourless crystalline solid. **Rf** (9:1 pentane:diethyl ether) 0.20; **mp** 116-118 °C; **IR** (NaCl, film) υ_{max} 3060, 3028, 1609, 1589, 1562, 1494, 1474, 1289, 1247, 911, 783, 742, 697 cm^{-1}; **^{1}H NMR** (400.2 MHz, CDCl$_3$): δ 7.82 – 7.76 (m, 1H, Ar*H*), 7.47 – 7.37 (m, 2H, Ar*H*), 7.24 – 7.15 (m, 3H, Ar*H*), 7.14 – 7.03 (m, 4H, Ar*H*), 7.00 – 6.94 (m, 3H, Ar*H*), 6.92 (d, *J* =

10.5 Hz, 1H, CC*H*CH), 6.76 (dd, *J* = 10.5, 9.6 Hz, 1H, CCHC*H*), 6.74 (d, *J* = 0.9 Hz, 1H, OCC*H*), 5.20 (d, *J* = 9.6 Hz, 1H, C*H*Ph); **^{13}C NMR** (100.05 MHz, $CDCl_3$) δ 194.7 (C), 160.3 (C), 141.6 (C), 140.9 (C), 138.4 (C), 136.7 (CH), 135.3 (CH), 134.5 (C), 134.1 (C), 130.8 (CH), 130.7 (CH), 129.7 (CH), 128.6 (CH), 128.4 (CH), 128.3 (3 x CH), 128.25 (CH), 128.0 (CH), 126.7 (CH), 126.2 (CH), 45.7 (CH); **MS** (ESI+): m/z 357 $[M+H]^+$; HRMS: found 357.1036 $C_{24}H_{18}ClO$ requires 357.1041.

(6*E*,9*Z*)-8-(*p*-Tolyl)-7-(trimethylsilyl)benzo[8]annulen-5(8*H*)-one (4aab)

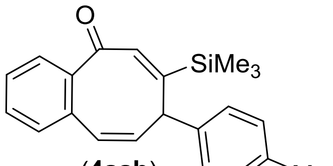

(**4aab**)

Synthesised from: trimethylsilylacetylene (**2a**) (235 µL, 1.70 mmol), *n*BuLi for acetylene deprotonation (1.2 mL, 1.50 M in hexanes, 1.80 mmol), 2-bromobenzaldehyde (**1a**) (189 µL, 1.62 mmol) *n*BuLi for bromine exchange (1.1 mL, 1.50 M in hexanes, 1.65 mmol), $CuBr{\cdot}SMe_2$ (167 mg, 0.81 mmol) 1-(1-chloroprop-2-yn-1-yl)-4-methylbenzene (**3b**) (130 mg, 0.79 mmol) using the general procedure. Flash column chromatography (20:1 pentane:diethyl ether) of the crude extract afforded the title compound (6*E*,9*Z*)-8-(*p*-tolyl)-7-(trimethylsilyl)benzo[8]annulen-5(8*H*)-one (**4aab**) (142 mg, 0.43 mmol) in 54% yield as a colourless crystalline solid. **Rf** (10:1 pentane:diethyl ether) 0.39; **mp** 100- 102 °C; **IR** (NaCl, film) υ_{max} 3024, 2953, 1611, 1590, 1515, 1276, 1249, 1008, 913, 841, 781, 760 cm^{-1}; **^{1}H NMR** (400.2 MHz, $CDCl_3$): δ 7.87 (dd, *J* = 7.6 Hz, 1.7 Hz, 1H, Ar*H*), 7.45 – 7.34 (m, 2H, Ar*H*), 7.19 (dd, *J* = 7.3 Hz, 1.7 Hz, 1H, Ar*H*), 7.11 – 7.03 (m, 4H, Ar*H*), 6.76 (d, *J* = 10.7, 1H, CC*H*CH), 6.73 (d, *J* = 1.2 Hz, 1H, OCC*H*), 6.65 (dd, *J* = 10.7 Hz, 9.7 Hz, 1H, CCHC*H*), 4.89 (d, *J* = 9.7 Hz, 1H, C*H*Ar), 2.29 (s, 3H, ArC*H*$_3$), -0.05 (s, 9H, Si(C*H*$_3$)$_3$); **^{13}C NMR** (100.05 MHz, $CDCl_3$) δ 195.3 (C), 165.6 (C), 141.7 (CH), 140.3 (C), 136.7 (C), 136.6 (C), 136.4 (CH), 135.1 (C), 131.0 (CH), 130.8 (CH), 129.4 (CH), 129.1 (CH), 128.6 (CH), 128.2 (CH), 127.9 (CH), 45.7 (CH), 21.1 (CH_3), 0.07 (CH_3); **MS** (ESI+): m/z 333 $[M+H]^+$; HRMS: found 333.1674 $C_{22}H_{25}OSi^+$ requires 333.1669.

(6*E*,9*Z*)-8-(4-Chlorophenyl)-7-(trimethylsilyl)benzo[8]annulen-5(8*H*)-one (4aac)

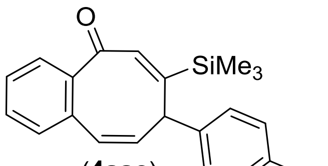

(**4aac**)

Synthesised from: trimethylsilylacetylene (**2a**) (235 µL, 1.70 mmol), *n*BuLi for acetylene deprotonation (1.2 mL, 1.50 M in hexanes, 1.80 mmol), 2-bromobenzaldehyde (**1a**) (189 µL, 1.62 mmol) *n*BuLi for bromine exchange (1.1 mL, 1.50 M in hexanes, 1.65 mmol), $CuBr{\cdot}SMe_2$ (167 mg, 0.81 mmol) and 1-chloro-4-(1-chloroprop-2-yn-1-yl)benzene (**3c**) (130 mg, 0.79 mmol) using the general procedure. Flash column chromatography (50:1 to 20:1 pentane:diethyl ether gradient elution) of the crude extract afforded the title compound (6*E*,9*Z*)-8-(4-chlorophenyl)-7-(trimethylsilyl)benzo[8]annulen-5(8*H*)-one (**4aac**) (152 mg, 0.43 mmol) in 54% yield as a colourless crystalline solid. **Rf** (10:1 pentane:diethyl ether) 0.22; **mp** 121 - 123 °C; **IR** (NaCl, film) υ_{max} 3026, 2954, 1611, 1591, 1492, 1276, 1250, 1093, 1014, 912, 841, 783 cm^{-1}; **^{1}H NMR** (400.2 MHz, $CDCl_3$): δ 7.84 – 7.76 (m, 1H, Ar*H*), 7.44 – 7.32 (m, 2H, Ar*H*), 7.22 – 7.13 (m, 3H, Ar*H*), 7.12 – 7.06 (m, 2H, Ar*H*), 6.78 (d, *J* = 10.6 Hz, 1H, CC*H*CH), 6.75 (d, *J* = 0.9 Hz, 1H, OCC*H*), 6.57 (dd, *J* = 10.6 Hz, 9.7 Hz, 1H, CCHC*H*), 4.86 (d, *J* = 9.7 Hz, 1H, C*H*Ar), 0.01 (s, 9H, Si(C*H*$_3$)$_3$); **^{13}C NMR** (100.05 MHz, $CDCl_3$) δ 195.4 (C), 163.9 (C), 142.0 (CH), 140.1 (C), 138.4 (C), 135.7 (CH), 134.8 (C), 132.7 (C), 131.0 (CH), 130.6 (CH), 129.5 (CH), 129.3 (CH), 129.3 (CH), 128.4 (CH), 128.1 (CH), 45.44 (CH), 0.1 (CH_3); **MS** (ESI+): m/z 353 $[M+H]^+$; HRMS: found 353.1120 $C_{21}H_{21}ClOSi^+$ requires 353.1123.

(6*E*,9*Z*)-8-(3-Methoxyphenyl)-7-(trimethylsilyl)benzo[8]annulen-5(8*H*)-one (4aad)

Synthesised from: trimethylsilylacetylene (**2a**) (235 µL, 1.70 mmol), *n*BuLi for acetylene deprotonation (1.1 mL, 1.60 M in hexanes, 1.76 mmol), 2-bromobenzaldehyde (**1a**) (189 µL, 1.62 mmol) *n*BuLi for bromine exchange (1.1 mL, 1.60 M in hexanes, 1.70 mmol), $CuBr{\cdot}SMe_2$ (167 mg, 0.81 mmol) 1-(1-chloroprop-2-yn-1-yl)-3-methoxybenzene (**3d**) (143 mg, 0.79 mmol) using the general procedure. Flash column chromatography (40:1 to 9:1 pentane:ethyl acetate gradient elution) of the crude extract afforded the title

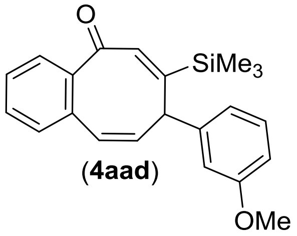
(**4aad**)

compound (6*E*,9*Z*)-8-(3-methoxyphenyl)-7-(trimethylsilyl)benzo[8]annulen-5(8*H*)-one (**4aad**) (116 mg, 0.33 mmol) in 42% yield as a colourless crystalline solid. **Rf** (19:1 pentane:ethyl acetate) 0.22; **mp** 84 - 86 °C; **IR** (NaCl, film) $υ_{max}$ 3026, 2954, 1609, 1588, 1490, 1276, 1249, 1158, 1052, 841, 783 cm^{-1}; **^{1}H NMR** (400.2 MHz, $CDCl_3$): δ 7.86 (dd, *J* = 7.6, 1.8 Hz, 1H, Ar*H*), 7.47 – 7.33 (m, 2H, Ar*H*), 7.23 – 7.12 (m, 2H, Ar*H*), 6.84 – 6.68 (m, 5H, 3Ar*H*, CC*H*CH, OCC*H*), 6.63 (dd, *J* = 10.7, 9.7 Hz, 1H, CCHC*H*), 4.90 (d, *J* = 9.7 Hz, 1H, C*H*Ar), 3.76 (s, 3H, OCH_3), -0.02 (s, 9H, Si(CH_3)$_3$); **^{13}C NMR** (100.05 MHz, $CDCl_3$) δ 195.2 (C), 165.0 (C), 159.7 (C), 141.9 (CH), 141.4 (C), 140.2 (C), 136.0 (CH), 135.0 (C), 131.0 (CH), 130.8 (CH), 129.4 (CH), 129.4 (CH), 128.8 (CH), 128.0 (CH), 120.6 (CH), 114.3 (CH), 112.4 (CH), 55.4 (CH_3), 45.9 (CH), 0.0 (CH_3); **MS** (ESI+): m/z 349 $[M+H]^+$; HRMS: found 349.1612 $C_{22}H_{25}O_2Si^+$ requires 349.1618; **CHN** Anal. calcd. for $C_{22}H_{24}O_2Si$ C: 75.82, H: 6.94%; found C: 75.76, H: 7.00%.

3-((5*Z*,8*E*)-10-Oxo-8-(trimethylsilyl)-7,10-dihydrobenzo[8]annulen-7-yl)benzonitrile (4aae)

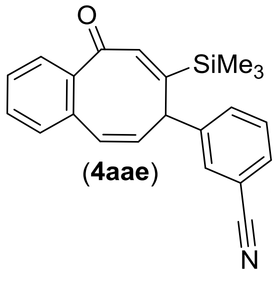
(**4aae**)

Synthesised from: trimethylsilylacetylene (**2a**) (235 µL, 1.70 mmol), *n*BuLi for acetylene deprotonation (1.1 mL, 1.60 M in hexanes, 1.76 mmol), 2-bromobenzaldehyde (**1a**) (189 µL, 1.62 mmol) *n*BuLi for bromine exchange (1.1 mL, 1.60 M in hexanes, 1.76 mmol), CuBr·SMe_2 (167 mg, 0.81 mmol) 3-(1-chloroprop-2-yn-1-yl)benzonitrile (**3e**) (138 mg, 0.79 mmol) using the general procedure with one exception. After the addition of 3-(1-chloroprop-2-yn-1-yl)benzonitrile the reaction temperature was left at -50 °C for 16 hours before quenching with pH = 7 ammonia buffered saturated aqueous ammonium chloride solution. Flash column chromatography (gradient elution: 9:1 to 4:1 pentane:diethyl ether) of the crude extract afforded the title compound 3-((5*Z*,8*E*)-10-oxo-8-(trimethylsilyl)-7,10-dihydrobenzo[8]annulen-7-yl)benzonitrile (**4aae**) (113 mg, 0.33 mmol) in 42% yield as a colourless crystalline solid. **Rf** (4:1 pentane:diethyl ether) 0.18; **mp** 155 - 157 °C; **IR** (NaCl, film) $υ_{max}$ 3061, 2955, 2897, 2229, 1611, 1589, 1480, 1277, 1250, 1010, 901, 841, 747 cm^{-1}; **^{1}H NMR** (400.2 MHz, $CDCl_3$): δ 7.69 – 7.60 (m, 1H, Ar*H*), 7.41 – 7.22 (m, 6H, Ar*H*), 7.09 – 7.01 (m, 1H, Ar*H*), 6.82 (d, *J* = 0.6 Hz, 1H, OCC*H*), 6.81 (d, *J* = 10.8 Hz, 1H, CC*H*CH), 6.51 (dd, *J* = 10.8, 9.4 Hz, 1H, CCHC*H*), 4.87 (d, *J* = 9.4 Hz, 1H, C*H*Ar), 0.04 (s, 9H, Si(CH_3)$_3$) ; **^{13}C NMR** (100.05 MHz, $CDCl_3$) δ 195.6 (C), 162.1 (C), 142.6 (CH), 141.4 (C), 140.1 (C), 134.9 (CH), 134.3 (C), 132.6 (CH), 131.4 (CH), 130.8 (CH), 130.4 (CH), 130.2 (CH), 130.0 (CH), 128.9 (CH), 128.7 (CH), 128.3 (CH), 118.8 (C), 112.2 (C), 45.5 (CH), 0.7 (CH_3); **MS** (ESI+): m/z 344 $[M+H]^+$; HRMS: found 344.1462 $C_{22}H_{22}NOSi^+$ requires 344.1465.

(6*E*,9*Z*)-8-(Thiophen-3-yl)-7-(trimethylsilyl)benzo[8]annulen-5(8*H*)-one (4aaf)

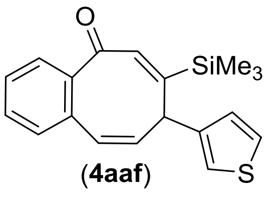
(**4aaf**)

Synthesised from: trimethylsilylacetylene (**2a**) (235 µL, 1.70 mmol), *n*BuLi for acetylene deprotonation (1.1 mL, 1.60 M in hexanes, 1.76 mmol), 2-bromobenzaldehyde (**1a**) (189 µL, 1.62 mmol) *n*BuLi for bromine exchange (1.1 mL, 1.60 M in hexanes, 1.76 mmol), CuBr·SMe_2 (167 mg, 0.81 mmol) 3-(1-chloroprop-2-yn-1-yl)thiophene (**3f**) (124 mg, 0.79 mmol) using the general procedure. Flash column chromatography (19:1 pentane:diethyl ether) of the crude extract afforded the title compound (6E,9Z)-8-(thiophen-3-yl)-7-(trimethylsilyl)benzo[8]annulen-5(8*H*)-one (**4aaf**) (134 mg, 0.41 mmol) in 52% yield as a colourless crystalline solid. **Rf** (9:1 pentane:diethyl ether) 0.31; **mp** 111 - 113 °C; **IR** (NaCl, film) $υ_{max}$ 3104, 3025, 2953, 2896, 1610, 1589, 1277, 1249, 1008, 845, 785, 743, 691 cm^{-1}; **^{1}H NMR** (400.2 MHz, $CDCl_3$): δ 7.83 (dd, *J* = 7.6, 1.7 Hz, 1H, Ar*H*), 7.46 – 7.34 (m, 2H, Ar*H*), 7.23 – 7.16 (m, 2H, Ar*H* Ar*H*-thiophene), 7.00 – 6.95 (m, 1H, Ar*H*-thiophene), 6.87 (dd, *J* = 4.9, 1.2 Hz, 1H, Ar*H*-thiophene), 6.74 (d, *J* = 10.7 Hz, 1H, CC*H*CH), 6.71 (d, *J* = 1.2 Hz, 1H, OCC*H*), 6.56 (dd, *J* = 10.7, 9.6 Hz, 1H, CCHC*H*), 4.86 (d, *J* = 9.6 Hz, 1H, C*H*Ar), 0.00 (s, 9H, Si(CH_3)$_3$); **^{13}C NMR** (100.05 MHz, $CDCl_3$) δ 195.1

(C), 164.5 (C), 141.3 (CH), 140.5 (C), 140.0 (C), 136.5 (CH), 134.7 (C), 130.9 (CH), 130.7 (CH), 129.2 (CH), 128.5 (2 x CH), 127.9 (CH), 125.6 (CH), 121.4 (CH), 42.4 (CH), 0.4 (CH_3); **MS** (ESI+): m/z 325 $[M+H]^+$; HRMS: found 325.1079 $C_{19}H_{21}OSSi^+$ requires 325.1077; **CHN** Anal. calcd. for $C_{19}H_{20}OSSi$ C: 70.32, H: 6.21%; found C: 70.31, H: 6.20%.

(6*E*,9*Z*)-8-(Naphthalen-1-yl)-7-(trimethylsilyl)benzo[8]annulen-5(8*H*)-one (4aag)

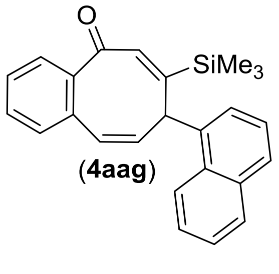

(**4aag**)

Synthesised from: trimethylsilylacetylene (**2a**) (235 µL, 1.70 mmol), *n*BuLi for acetylene deprotonation (1.1 mL, 1.60 M in hexanes, 1.76 mmol), 2-bromobenzaldehyde (**1a**) (189 µL, 1.62 mmol) *n*BuLi for bromine exchange (1.1 mL, 1.60 M in hexanes, 1.76 mmol), CuBr·SMe_2 (167 mg, 0.81 mmol) 1-(1-chloroprop-2-yn-1-yl)naphthalene (**3g**) (159 mg, 0.79 mmol) using the general procedure. Flash column chromatography (neat pentane to 19:1 pentane:diethyl ether) of the crude extract afforded the title compound (6*E*,9*Z*)-8-(naphthalen-1-yl)-7-(trimethylsilyl)benzo[8]annulen-5(8*H*)-one (**4aag**) (69 mg, 0.19 mmol) in 24% yield as a colourless crystalline solid. **Rf** (9:1 pentane:diethyl ether) 0.38; **mp** 154 - 156 °C; **IR** (NaCl, film) υ_{max} 3052, 2954, 2895, 1609, 1590, 1278, 1248, 1011, 910, 840, 783 cm^{-1}; **^{1}H NMR** (400.2 MHz, $CDCl_3$): δ 8.04 – 7.97 (m, 1H, Ar*H*), 7.85 – 7.68 (m, 3H, Ar*H*), 7.59 (d, *J* = 7.2 Hz, 1H, Ar*H*), 7.47 – 7.38 (m, 5H, Ar*H*), 7.22 – 7.14 (m, 1H, Ar*H*), 6.95 (dd, *J* = 10.7, 9.7 Hz, 1H, CCHC*H*), 6.85 (d, *J* = 1.1 Hz, 1H, OCC*H*), 6.83 (d, *J* = 10.7 Hz, 1H, CC*H*CH), 5.60 (d, *J* = 9.7 Hz, 1H, ArC*H*), -0.33 (s, 9H, Si(C*H*$_3$)$_3$); **^{13}C NMR** (100.05 MHz, $CDCl_3$) δ 195.0 (C), 166.9 (C), 141.7 (CH), 140.5 (C), 135.6 (CH), 135.1 (2 x C), 134.0 (C), 132.9 (C), 131.2 (CH), 130.9 (CH), 129.3 (CH), 128.9 (CH), 128.3 (CH), 128.2 (CH), 128.0 (CH), 126.4 (CH), 125.9 (CH), 125.0 (CH), 124.2 (CH), 123.9 (CH), 43.0 (CH), -0.4 (CH_3); **MS** (ESI+): m/z 369 $[M+H]^+$; HRMS: found 369.1664 $C_{25}H_{25}OSi^+$ requires 369.1669.

(6*E*,9*Z*)-8-(Naphthalen-2-yl)-7-(trimethylsilyl)benzo[8]annulen-5(8*H*)-one (4aah)

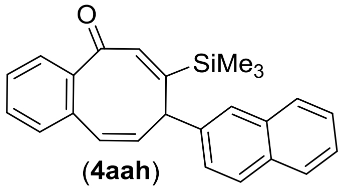

(**4aah**)

Synthesised from: trimethylsilylacetylene (**2a**) (235 µL, 1.70 mmol), *n*BuLi for acetylene deprotonation (1.1 mL, 1.60 M in hexanes, 1.76 mmol), 2-bromobenzaldehyde (**1a**) (189 µL, 1.62 mmol) *n*BuLi for bromine exchange (1.1 mL, 1.60 M in hexanes, 1.76 mmol), CuBr·SMe_2 (167 mg, 0.81 mmol) 2-(1-chloroprop-2-yn-1-yl)naphthalene (**3h**) (159 mg, 0.79 mmol) using the general procedure. Flash column chromatography (gradient elution, neat pentane to 19:1 pentane:diethyl ether) of the crude extract afforded the title compound (6*E*,9*Z*)-8-(naphthalen-2-yl)-7-(trimethylsilyl)benzo[8]annulen-5(8*H*)-one (**4aah**) (140 mg, 0.38 mmol) in 48% yield as a yellow crystalline solid. **Rf** (9:1 pentane:diethyl ether) 0.33; **mp** 111 - 113 °C; **IR** (NaCl, film) υ_{max} 3056, 2954, 2896, 1609, 1591, 1507, 1277, 1249, 1010, 841, 750 cm^{-1}; **^{1}H NMR** (400.2 MHz, $CDCl_3$): δ 7.90 – 7.84 (m, 1H, Ar*H*), 7.81 – 7.69 (m, 3H, Ar*H*), 7.64 (s, 1H, Ar*H*), 7.47 – 7.40 (m, 2H, Ar*H*), 7.39 – 7.31 (m, 2H, Ar*H*), 7.28 (dd, *J* = 8.5, 1.7 Hz, 1H, Ar*H*), 7.22 – 7.16 (m, 1H, Ar*H*), 6.84 (d, *J* = 10.7 Hz, 1H, CC*H*CH), 6.81 (d, *J* = 1.1 Hz, 1H, OCC*H*), 6.78 (dd, *J* = 10.7, 9.0 Hz, 1H, CCHC*H*), 5.08 (d, *J* = 9.0 Hz, 1H, C*H*Ar), -0.05 (s, 9H, Si(C*H*$_3$)$_3$); **^{13}C NMR** (100.05 MHz, $CDCl_3$) δ 195.4 (C), 164.7 (C), 141.9 (CH), 140.2 (C), 137.3 (C), 136.1 (CH), 135.0 (C), 133.2 (C), 132.4 (C), 131.0 (CH), 130.7 (CH), 129.4 (CH), 129.1 (CH), 128.0 (CH), 128.0 (CH), 127.8 (CH), 127.7 (CH), 127.2 (CH), 126.3 (CH), 126.3 (CH), 125.9 (CH), 46.2 (CH), 0.0 (CH_3); **MS** (ESI+): m/z 391 $[M+Na]^+$; HRMS: found 391.1484 $C_{25}H_{24}NaOSi^+$ requires 391.1489.

(6*E*,9*Z*)-8-(4-(*tert*-Butyl)phenyl)-7-(trimethylsilyl)benzo[8]annulen-5(8*H*)-one (4aai)

Synthesised from: trimethylsilylacetylene (**2a**) (235 µL, 1.70 mmol), *n*BuLi for acetylene deprotonation (1.1 mL, 1.60 M in hexanes, 1.76 mmol), 2-bromobenzaldehyde (**1a**) (189 µL, 1.62 mmol) *n*BuLi for bromine exchange (1.1 mL, 1.60 M in hexanes, 1.76 mmol), CuBr·SMe_2 (167 mg, 0.81 mmol) 1-(*tert*-butyl)-4-(1-

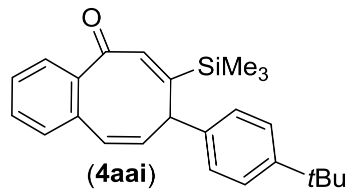

(**4aai**)

chloroprop-2-yn-1-yl)benzene (**3i**) (163 mg, 0.79 mmol) using the general procedure. Flash column chromatography (gradient elution, neat pentane to 33:1 pentane:diethyl ether) of the crude extract afforded the title compound (6*E*,9*Z*)-8-(4-(*tert*-butyl)phenyl)-7-(trimethylsilyl)benzo[8]annulen-5(8*H*)-one (**4aai**) (154 mg, 0.41 mmol) in 52% yield as a yellow crystalline solid. **Rf** (9:1 pentane:diethyl ether) 0.59; **mp** 95 - 97 ^{o}C; **IR** (NaCl, film) υ_{max} 3057, 3027, 2961, 2901, 2868, 1611, 1592, 1510, 1275, 1249, 915, 841, 783, 763 cm^{-1}; **^{1}H NMR** (400.2 MHz, $CDCl_3$): 7.86 – 7.79 (m, 1H, Ar*H*), 7.43 – 7.33 (m, 2H, Ar*H*), 7.30 – 7.24 (m, 2H, Ar*H*), 7.17 (dd, *J* = 7.1, 1.9 Hz, 1H, Ar*H*), 7.15 – 7.10 (m, 2H, Ar*H*), 6.77 (d, *J* = 10.7 Hz, 1H, CC*H*CH), 6.77 (d, *J* = 1.2 Hz, 1H, OCC*H*), 6.66, (dd, *J* = 10.6, 9.7 Hz, 1H, CCHC*H*), 4.93 (d, *J* = 9.7 Hz, 1H, C*H*Ar), 1.28 (s, 9H, C(CH_3)$_3$), -0.04 (s, 9H,Si(CH_3)$_3$); **^{13}C NMR** (100.05 MHz, $CDCl_3$) δ 195.2 (C), 166.0 (C), 150.1 (C), 141.8 (CH), 140.4 (C), 136.7 (C), 136.3 (CH), 135.0 (C), 130.8 (CH), 130.7 (CH), 129.1 (CH), 128.4 (CH), 128.0 (CH), 127.9 (CH), 125.2 (CH), 45.6 (CH), 34.5 (C), 31.4 (CH_3), -0.1 (CH_3); **MS** (ESI+): m/z 375 $[M+H]^+$; HRMS: found 375.2996 $C_{25}H_{31}OSi^+$ requires 375.2139; **CHN** Anal. calcd. for $C_{25}H_{30}OSi$ C: 80.16, H: 8.07%; found C: 80.21, H: 8.19%.

(*E*)-1-(2-(3-Phenyl-2-(thiophen-2-yl)allyl)pyridin-3-yl)-3-(trimethylsilyl)prop-2-yn-1-ol (7)

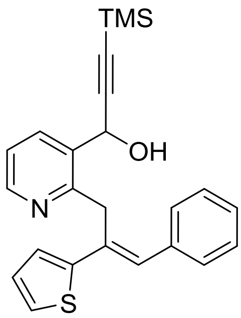

(**7**)

mp 148-149 °C; **IR** (NaCl, $CHCl_3$) υ_{max} 3691, 3597, 3062, 3010, 2965, 2173, 1600, 1587, 1574, 1492, 1432, 1367, 1252, 1040, 974, 921, 848 cm^{-1}; **^{1}H NMR** (400.2 MHz, $CDCl_3$) δ 8.54 (dd, *J* = 4.8, 1.7 Hz, 1H, Ar*H*), 7.97 (dd, *J* = 7.8, 1.7 Hz, 1H, Ar*H*), 7.40 – 7.35 (m, 3H, Ar*H*), 7.31 – 7.24 (m, 2H, Ar*H*), 7.25 – 7.17 (m, 2H, Ar*H*), 7.09 (dd, *J* = 5.1, 1.1 Hz, 1H, Ar*H*), 6.93 (dd, *J* = 3.6, 1.1 Hz, 1H, Ar*H*), 6.85 (m, 1H, PhC*H*C), 5.66 (d, *J* = 4.9 Hz, 1H, ArC*H*OH), 4.37 (s, 2H, ArCH_2C), 2.24 (d, *J* = 4.9 Hz, 1H, O*H*), 0.14 (s, 9H, Si(CH_3)$_3$); **^{13}C NMR** (100.6 MHz, $CDCl_3$) δ 157.4 (C), 149.3 (CH), 146.7 (C), 137.4 (C), 134.5 (CH), 133.6 (C), 132.2 (C), 128.8 (CH), 128.7 (CH), 128.4 (CH), 127.4 (CH), 127.1 (CH), 124.3 (CH), 123.8 (CH), 121.7 (CH), 103.4 (C), 92.8 (C), 62.1 (CH), 36.6 (CH_2), -0.2 (CH_3); **MS** (ESI+): m/z 404 $[M+H]^+$; HRMS: found 404.1522 $C_{24}H_{26}NOSSi^+$ requires 404.1499. X-ray data at: CCDC 1405855. Selected data in Figure S6.

(*E*)-1-(1-Methyl-2-(3-phenylprop-1-en-1-yl)-1*H*-indol-3-yl)-3-(trimethylsilyl)prop-2-yn-1-one (8)

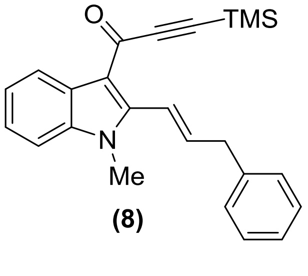

(8)

IR (NaCl, $CHCl_3$) υ_{max} 3028, 2958, 1590, 1575, 1507, 1471, 1404, 1251, 1086, 846, 746 cm^{-1}; **^{1}H NMR** (400.2 MHz, $CDCl_3$) δ 8.58 – 8.51 (m, 1H, Ar*H*), 7.36 – 7.27 (m, 7H, Ar*H*), 7.24 – 7.18 (m, 1H, Ar*H*), 6.42 (dd, *J* = 16.1, 1.2 Hz, 1H, ArC*H*CH), 6.34 (dt, *J* = 16.1, 5.8 Hz, 1H, CHC*H*CH$_2$), 4.35 (dd, *J* = 5.8, 1.2 Hz, 2H, CHCHCH_2), 3.76 (s, 3H, NCH_3), 0.30 (s, 9H, Si(CH_3)$_3$; **^{13}C NMR** (100.6 MHz, $CDCl_3$) δ 171.4 (C), 147.6 (C), 137.0 (C), 136.9 (C), 132.2 (CH), 128.7 (CH), 127.7 (CH), 126.5 (C), 126.3 (CH), 124.8 (CH), 123.3 (CH), 122.9 (CH), 121.9 (CH), 114.5 (C), 109.6 (CH), 104.5 (C), 96.9 (C), 30.1 (CH_3), 29.3 (CH_2), -0.5 (CH_3); **MS** (ESI+): m/z 372 $[M+H]^+$; HRMS: found 372.1775 $C_{24}H_{26}NOSi^+$ requires 372.1778.

General procedure for the one-pot carboannulation and electrophile addition to 9-11

Acetylene **2** (1.62 – 1.70 mmol) was dissolved in tetrahydrofuran (2.3 mL) under argon and cooled to -50 ^{o}C. *n*-Butyllithium (1.6 M in hexanes, 1.62 – 1.79 mmol) was then added to the solution and the mixture stirred for 20 minutes. Neat bromoaldehyde **1** (1.62 mmol) was added to the reaction mixture. Thin-layer chromatography (9:1 pentane:diethyl ether) was used to confirm consumption of the aldehyde. After 60 minutes additional *n*-butyllithium (1.6 M in hexanes, 1.62 – 1.70 mmol) was added forming yellow, orange

or dark coloured solutions. After 15 minutes solid copper(I) bromide dimethyl sulfide (167 mg, 0.81 mmol) was then added under a cushion of argon, normally forming tan suspensions/dark solutions at -50 °C. After a further 1 hour neat propargylic chloride **3** (0.79 mmol) was added to the reaction mixture, which was then allowed to warm slowly to -10 °C over a period of 30 minutes. Once the reaction mixture reached -10 °C it was kept at this temperature for a further 1 hour. Electrophile was then added and the reaction mixture allowed to warm to room temperature over 1 hour where pH = 7 ammonia buffered saturated aqueous ammonium chloride solution (5 mL) was added and extracted with ethyl acetate (3 × 10 mL). The combined organic extracts were dried with magnesium sulfate and concentrated *in vacuo* to afford the crude product. Flash column chromatography (20:1 pentane:diethyl ether) of the crude extract afforded the title compound **4**. In many cases the 8-ring products could be crystallised from pentane, diethyl ether or methanol.

(6*Z*,9*Z*)-6-Iodo-8-phenyl-7-(trimethylsilyl)benzo[8]annulen-5(8*H*)-one (9)

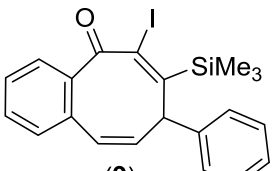

(**9**)

Synthesised from: trimethylsilylacetylene (**2a**) (235 µL, 1.70 mmol), *n*BuLi for acetylene deprotonation (1.1 mL, 1.60 M in hexanes, 1.76 mmol), 2-bromobenzaldehyde (**1a**) (189 µL, 1.62 mmol) *n*BuLi for bromine exchange (1.1 mL, 1.60 M in hexanes, 1.76 mmol), CuBr·SMe$_2$ (167 mg, 0.81 mmol) (1-chloroprop-2-yn-1-yl)benzene (**3a**) (119 mg, 0.79 mmol), trapped with a solution of iodine (1.00 g, 3.95 mmol) in diethyl ether (5 mL), stirred at room temperature for 45 minutes using the general procedure. Flash column chromatography (20:1 pentane:diethyl ether) of the crude extract afforded the title compound (6*Z*,9*Z*)-6-iodo-8-phenyl-7-(trimethylsilyl)benzo[8]annulen-5(8*H*)-one (**9**) (195 mg, 0.44 mmol) in 56% yield as a colourless solid. **Rf** (10:1 pentane:diethyl ether) 0.37; **mp** 146 - 148 °C; **IR** (NaCl, film) υ_{max} 3027, 2953, 2896, 1658, 1591, 1566, 1495, 1249, 847, 790, 745, 699 cm^{-1}; ^{1}**H NMR** (400.2 MHz, CDCl$_3$): δ 8.00 – 7.93 (m, 1H, Ar*H*), 7.64 – 7.56 (m, 1H, Ar*H*), 7.50 – 7.41 (m, 2H, Ar*H*), 7.36 – 7.21 (m, 5H, Ar*H*), 6.79 (dd, *J* = 11.4, 1.1 Hz, 1H, CC*H*CH), 6.59 (dd, *J* = 11.4, 8.7 Hz, 1H, CCHC*H*), 4.70 (ddd, *J* = 8.7, 1.1, 1.1 Hz, 1H, C*H*Ph), 0.08 (s, 9H, Si(C*H*$_3$)$_3$); ^{13}**C NMR** (100.05 MHz, CDCl$_3$) δ 199.0 (C), 152.0 (C), 140.4 (C), 135.6 (C), 134.8 (CH), 134.7 (C), 134.5 (CH), 133.0 (CH), 132.7 (CH), 132.3 (CH), 128.7 (CH), 128.2 (CH), 127.1 (CH), 126.8 (CH), 100.0 (C), 50.6 (CH), 0.87 (CH$_3$); **MS** (ESI+): m/z 445 [M+H]$^+$; HRMS: found 445.0480 $C_{21}H_{22}IOSi^+$ requires 445.0479; **CHN** Anal. calcd. for $C_{21}H_{21}IOSi$ C: 56.76, H: 4.76%; found C: 56.76, H: 4.69%.

(6*E*,9*Z*)-6-Methyl-8-phenyl-7-(trimethylsilyl)benzo[8]annulen-5(8*H*)-one (10)

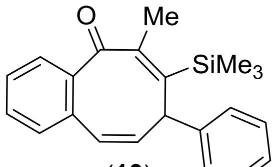

(**10**)

Synthesised from: trimethylsilylacetylene (**2a**) (235 µL, 1.70 mmol), *n*BuLi for acetylene deprotonation (1.1 mL, 1.60 M in hexanes, 1.76 mmol), 2-bromobenzaldehyde (**1a**) (189 µL, 1.62 mmol) *n*BuLi for bromine exchange (1.1 mL, 1.60 M in hexanes, 1.76 mmol), CuBr·SMe$_2$ (167 mg, 0.81 mmol) (1-chloroprop-2-yn-1-yl)benzene (**3a**) (119 mg, 0.79 mmol), trapped with iodomethane (0.49 mL, 7.9 mmol) using the general procedure. Flash column chromatography (neat pentane to 50:1 pentane:diethyl ether gradient elution) of the crude extract afforded the title compound (6*E*,9*Z*)-6-methyl-8-phenyl-7-(trimethylsilyl)benzo[8]annulen-5(8*H*)-one (**10**) (123 mg, 0.37 mmol) in 47% yield as a colourless solid. **Rf** (9:1 pentane:diethyl ether) 0.42; **mp** 90 - 92 °C; **IR** (NaCl, film) υ_{max} 3027, 2953, 2897, 1653, 1593, 1495, 1277, 1262, 1250, 1156, 951, 840, 790, 723 cm^{-1}; ^{1}**H NMR** (500.1 MHz, CDCl$_3$): δ 8.00 – 7.95 (m, 1H, Ar*H*), 7.58 – 7.53 (m, 1H, Ar*H*), 7.45 – 7.40 (m, 2H, Ar*H*), 7.33 – 7.26 (m, 4H, Ar*H*), 7.24 – 7.18 (m, 1H, Ar*H*), 6.74 (d, *J* = 11.4 Hz, 1H, CC*H*CH), 6.67 (dd, *J* = 11.4, 8.5 Hz, 1H, CCHC*H*), 4.54 (d, *J* = 8.5 Hz, 1H, C*H*Ph), 2.00 (s, 3H, CC*H*$_3$), -0.03 (s, 9H, Si(C*H*$_3$)$_3$); ^{13}**C NMR** (125.8 MHz, CDCl$_3$) δ 207.3 (C), 144.5 (C), 142.4 (C), 141.8 (C), 138.2 (C), 137.3 (CH), 135.7 (C), 134.1 (CH), 132.2 (CH), 131.7 (CH), 131.3 (CH), 128.4 (CH), 127.7 (CH), 127.0 (CH), 126.6 (CH), 48.1 (CH), 18.6 (CH$_3$), 1.5 (CH$_3$); **MS** (ESI+): m/z 355 [M+Na]$^+$; HRMS: found 355.1495 $C_{22}H_{24}NaOSi^+$ requires 355.1489.

(6*Z*,9*Z*)-5-Oxo-8-phenyl-7-(trimethylsilyl)-5,8-dihydrobenzo[8]annulene-6-carboxylic acid (11)

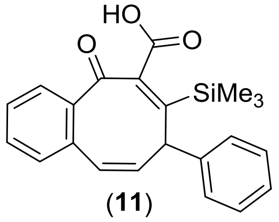

(**11**)

Synthesised from: trimethylsilylacetylene (**2a**) (235 µL, 1.70 mmol), *n*BuLi for acetylene deprotonation (1.1 mL, 1.60 M in hexanes, 1.76 mmol), 2-bromobenzaldehyde (**1a**) (189 µL, 1.62 mmol) *n*BuLi for bromine exchange (1.1 mL, 1.60 M in hexanes, 1.76 mmol), CuBr·SMe_2 (167 mg, 0.81 mmol) (1-chloroprop-2-yn-1-yl)benzene (**3a**) (119 mg, 0.79 mmol), trapped with solid carbon dioxide (approx. 1 g, 22.7 mmol) added in small portions as the reaction mixture warmed to room temperature using the general procedure. Elution through a small plug of silica (neat diethyl ether) following by trituration in diethyl ether afforded the title compound. A further crop of title compound was obtained by flash column chromatography (75:25:1 pentane:diethyl ether:acetic acid) of the trituration liquors to afford (6*Z*,9*Z*)-5-oxo-8-phenyl-7-(trimethylsilyl)-5,8-dihydrobenzo[8]annulene-6-carboxylic acid (**11**) (141 mg, 0.39 mmol) in 49% yield as a colourless solid. **Rf** (50:50:1 pentane:diethyl ether:acetic acid) 0.41; **mp** 200 – 202 °C; **IR** (ATR) $υ_{max}$ 2952, 1686, 1660, 1566, 1412, 1287, 1253, 850 cm^{-1}; **^{1}H NMR** (400.2 MHz, $(CD_3)_2SO$): δ 13.13 (br s, 1H, COO*H*), 7.82 (dd, *J* = 7.8, 1.3 Hz, 1H, Ar*H*), 7.69 (ddd, *J* = 7.8, 7.2, 1.5 Hz, 1H, Ar*H*), 7.60 (dd, *J* = 8.2, 1.0 Hz, 1H, Ar*H*), 7.58 – 7.51 (m, 1H, Ar*H*), 7.42 – 7.36 (m, 2H, Ar*H*), 7.32 – 7.26 (m, 1H, Ar*H*), 7.24 – 7.18 (m, 2H, Ar*H*), 6.94 (d, *J* = 11.3 Hz, 1H, CC*H*CH), 6.75 (dd, *J* = 11.3, 8.6 Hz, 1H, CCHC*H*), 4.49 (d, *J* = 8.6 Hz, 1H, C*H*Ph), -0.05 (s, 9H, Si(CH_3)$_3$); **^{13}C NMR** (100.05 MHz, $(CD_3)_2SO$) δ 201.6 (C), 166.2 (C), 158.9 (C), 141.4 (C), 140.5 (C), 139.3 (C), 135.2 (CH), 134.7 (CH), 134.6 (C), 133.0 (CH), 133.0 (CH), 131.6 (CH), 129.1 (CH), 128.8 (CH), 127.5 (CH), 127.0 (CH), 48.1 (CH), 2.0 (CH_3); **MS** (ESI+): m/z 385 $[M+Na]^+$; HRMS: found 385.1062 $C_{22}H_{22}NaO_3Si^+$ requires 385.1230.

Deuterium Labelling Experiments

(2-Bromophenyl)methanol-d$_1$

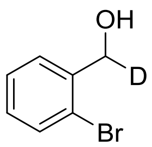

Lithium aluminium deuteride (272 mg, 98 atom% D, 6.48 mmol) was suspended in THF (10 mL) under argon and cooled to 0 °C. 2-Bromobenzaldehyde (800 mg, 4.32 mmol) was then added dropwise over a period of 10 minutes, after which the reaction mixture was stirred for a further 30 minutes. The reaction mixture was then warmed to room temperature, quenched with water (5 mL) and extracted with diethyl ether (3 × 10 mL). The combined organic extracts were dried with magnesium sulfate, filtered and concentrated *in vacuo* to provide the title compound (2-bromophenyl)methanol-d$_1$ (803 mg, 4.27 mmol, >95 atom% D) in 99% yield as a colourless crystalline solid which was used without further purification. **Rf** (9:1 pentane:diethyl ether) 0.47; **mp** 73-76 °C; **IR** (NaCl, film) $υ_{max}$ 3424, 2867, 2386, 2144, 2117, 2077, 1680, 1670, 1587, 1566, 1460, 1438, 1392, 1289, 1266, 1211, 1160, 1111, 1063, 1044, 1020 cm^{-1}; **^{1}H NMR** (400.1 MHz, $CDCl_3$): δ 7.55 (dd, *J* = 8.0, 1.2 Hz, 1H), 7.48 (dd, *J* = 7.6, 1.7 Hz, 1H), 7.33 (td, *J* = 7.6, 1.2 Hz, 1H), 7.17 (td, *J* = 7.6, 1.7 Hz, 1H), 4.86 – 4.66 (m, 1H), 2.10 (d, *J* = 6.2 Hz, 1H); **^{2}H NMR** (61.4 MHz, $CHCl_3$): δ 4.75 (s); **^{13}C NMR** (100.6 MHz, $CDCl_3$): δ 139.8 (C), 132.7 (CH), 129.3 (CH), 129.1 (CH), 127.8 (CH), 122.7 (C), 64.9 (t, *J* = 22.1 Hz, CDH); **MS** (EI+): m/z 189 $[M]^+$; HRMS: found 188.9723 C_7H_6BrDO requires 188.9732.

2-Bromobenzaldehyde-d$_1$ (d$_1$-1a)

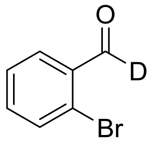

(2-Bromophenyl)methanol-d$_1$ (724 mg, >95 atom% D, 3.85 mmol) was dissolved in dichloromethane (14 mL) under argon at room temperature. Pyridinium chlorochromate (1.24 g, 5.75 mmol) was then added in one portion, after which the reaction mixture was stirred for a further 2.0 hours. The reaction mixture was then poured on to a wet column of silica gel (4:1 hexane:ethyl acetate), eluted and concentrated *in vacuo* to provide the title compound 2-

bromobenzaldehyde-d_1 (700 mg, 3.76 mmol, 83 atom% D) in 98% yield as a colourless oil which was used without further purification. **Rf** (9:1 pentane:diethyl ether) 0.92; **IR** (NaCl, film) $υ_{max}$ 3301, 3205, 2895, 2349, 2150, 1956, 1691, 1591, 1569, 1471, 1462, 1439, 1334, 1307, 1264, 1200, 1129, 1107, 1066, 1041, 1024 cm^{-1}; **^{1}H NMR** (400.1 MHz, $CDCl_3$): δ 10.37 (s, 0.17H), 7.97 – 7.88 (m, 1H), 7.70 – 7.61 (m, 1H), 7.50 – 7.38 (m, 2H); **^{2}H NMR** (61.4 MHz, $CHCl_3$): δ 10.40 (s); **^{13}C NMR** (100.6 MHz, $CDCl_3$): δ 191.9 (CHO), 191.5 (t, *J* = 28.0 Hz, CDO), 135.4 (CH), 133.9 (CH), 133.6 (*C*C(O)H) 133.5 (t, *J* = 3.5 Hz, *C*C(O)D) , 129.9 (CH), 128.0 (CH), 127.1 (C); **MS** (EI+): m/z 187 $[M]^+$; HRMS: found 186.9566 C_7H_4BrDO requires 186.9571.

(6*E*,9*Z*)-8-Phenyl-7-(trimethylsilyl)benzo[8]annulen-5(8*H*)-one-9-d_1 (d_1-4aaa)

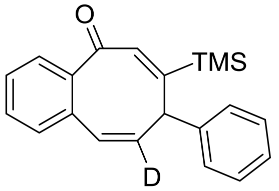

(**d_1-4aaa**)

Synthesised from: trimethylsilylacetylene (229 µL, 1.62 mmol), *n*BuLi for acetylene deprotonation (1.0 mL, 1.62 M in hexanes, 1.62 mmol), 2-bromobenzaldehyde-d_1 (301 mg, 1.62 mmol), *n*BuLi for bromine exchange (1.0 mL, 1.62 M, 1.62 mmol), $CuBr.SMe_2$ (167 mg, 0.810 mmol) and (1-chloroprop-2-yn-1-yl)benzene (119 mg, 0.790 mmol) using the general procedure. Flash column chromatography (20:1 pentane:diethyl ether) afforded the title compound (6*E*,9*Z*)-8-phenyl-7-(trimethylsilyl)benzo[8]annulen-5(8*H*)-one-d_1 (148 mg, 0.463 mmol, 69 atom% D) in 59% yield as a pale yellow crystalline solid. **Rf** (10:1 pentane:diethyl ether) 0.55; **mp** 97-100 °C; **IR** (NaCl, film) υmax 3441, 3061, 3028, 2956, 2897, 2361, 1945, 1883, 1633, 1611, 1496, 1476, 1449, 1410, 1277, 1250, 1210, 1120, 1093, 1079, 1050, 1008 cm^{-1}; **^{1}H NMR** (400.2 MHz, CDCl3): δ 7.92 – 7.80 (m, 1H, Ar*H*), 7.46 – 7.33 (m, 2H, Ar*H*), 7.31 – 7.14 (m, 6H, Ar*H*), 6.78 (d, *J* = 10.6 Hz, 0.31H, CC*H*CH), 6.78 (s, 0.69H, CC*H*CD), 6.76 (d, *J* = 1.1 Hz, 1H, OCC*H*), 6.66 (dd, *J* = 10.6, 9.7 Hz, 0.31H, CCHC*H*), 4.94 (d, *J* = 7.0 Hz, 1H, C*H*Ph), -0.02 – -0.09 (m, 9H, $Si(CH_3)_3$); **^{2}H NMR** (61.4 MHz, $CHCl_3$): δ 6.72 (s); **^{13}C NMR** (100.6 MHz, $CDCl_3$) δ 195.2 (C), 165.2 (C), 141.8 (CH), 140.3 (C), 139.8 (C), 136.1 (CH, residual non-D), 135.8 (t, *J* = 24.5 Hz, CD), 135.0 (C), 131.0 (CH), 130.8 (CH), 129.3 (CH), 128.8 (CH, residual non-D), 128.7 (CH), 128.4 (CH), 128.3 (CH), 128.0 (CH), 127.0 (CH), 45.9 (CH), -0.1 (CH_3); **MS** (ESI+): m/z 320 $[M+H]^+$; HRMS: found 320.1570 $C_{21}H_{22}DOSi$ requires 320.1575.

Crystallographic Data

Data were collected by the School of Chemistry, University of Nottingham Crystallography Service using the standard methods described in the compound CIF files. The structures were solved and refined by standard methods. CIF files of the compounds analysed (**4aaa**, **4haa**, **4jaa** and **7**) have been uploaded to the Cambridge Crystallographic Database with codes CCDC 1405847, CCDC 1405851, CCDC 1405854 and CCDC 1405855 respectively (available from: www.ccdc.cam.ac.uk).

4. Computational studies

All calculations were carried out using Gaussian 09 revD.01.[S13] Default settings were used except that 'tight' convergence and 'UltraFine' integration grid were specified for calculations involving M06-2X. Initial structure searches were performed using B3LYP/6-31G*,[S14] and resulting structures were further optimised using M06-2X/6-31G(d)[S15] and CBS-QB3[S16] as specified below. M06-2X/6-311+G** single-point energies were computed for all M06-2X/6-31G* geometries, and combined with M06-2X/6-31G(d) free energy corrections to obtain M06-2X/6-311+G**//M06-2X/6-31G* free energies. Transition-state structures were all characterised by a single imaginary vibrational frequency. CYLView[S17] was used to generate all the 3D-molecular structure figures.

To minimise computational effort, and because the exact nature of the organometallics involved in these reactions is not known, the DFT calculations were initially run on the unsupported anions **C’**, **F’**, **G’** where the TMS group has been replaced by a hydrogen atom – these correlate to the intermediates **C**, **F** and **G** in the main paper (Scheme 3). These calculations were carried out on the ‘naked ion’ model system using both M06-2X/6-311+G**//M06-2X/6-31G* and the composite method CBS-QB3. As coupling of organocopper species of type **D** with propargylic chlorides can result in either: (a) the product where the migrating C-H and terminal substituent on the allene are *syn*, or (b) the equivalent *anti* product both reaction coordinates were investigated (Figure S1).

Figure S1. Calculated reaction coordinates for the annulation reaction: (a) from *syn*-**C’**; (b) from *anti*-**C’** using M06-2X/6-311+G**//M06-2X/6-31G* and CBS-QB3. Energy values obtained using CBS-QB3 are given in parentheses.

(a) Annulation from *syn*-**C’**.

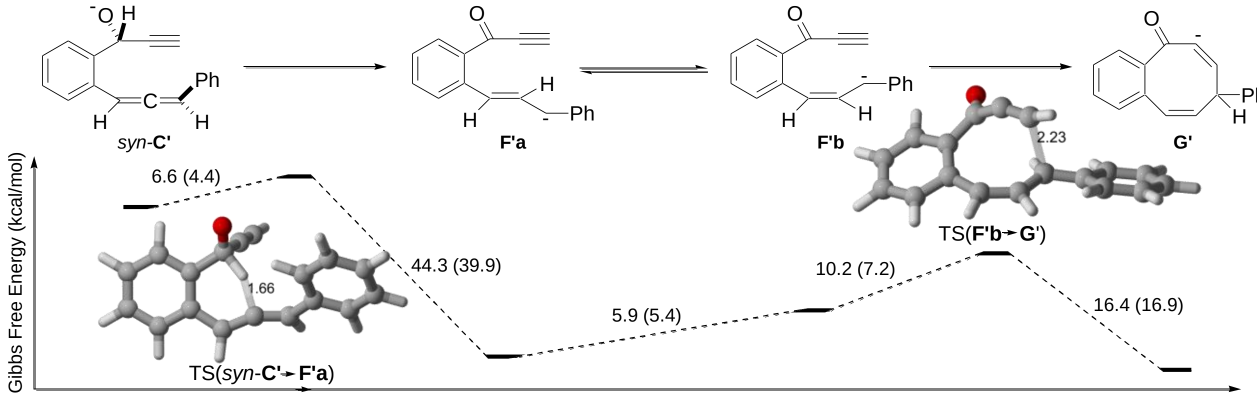

(b) Annulation from *anti*-**C’**.

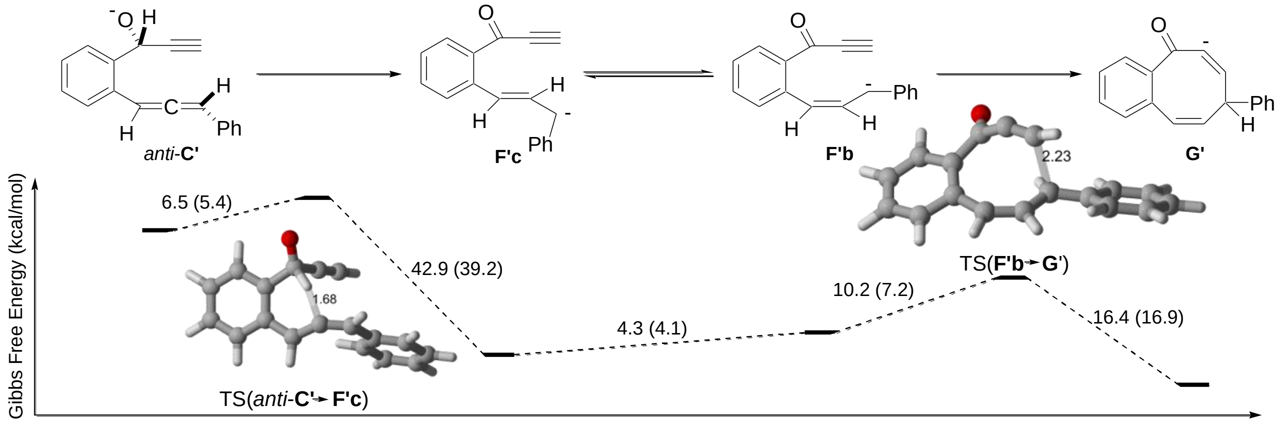

The calculations suggests that Cannizzaro hydride shift is facile and that equilibration between the allylic forms of **F’** is viable. Annulation of **F’b** to the non-classical enolate **G’** is predicted to be the rate limiting step by both sets of calculation. There was reasonable agreement between the two computational methods used, although M06-2X/6-311+G**//M06-2X/6-31G* predicted higher barrier to reaction for both the hydride shift and cyclisation for both diastereoisomers.

It was not practical to use CBS-QB3 to investigate structures incorporating the TMS-group (intermediates **C**, **F** and **G** in Scheme 3 of the main paper), but the less expensive M06-2X/6-311+G**//M06-2X/6-31G* method was used. As would be expected, inclusion of the bulky TMS-substituent was predicted to result in a small increase (*ca*. 2 kcal/mol) in the barrier to both the hydride shift and the subsequent cyclisation (Figure S2).

Figure S2. Calculated reaction coordinates for the annulation reaction: (a) from *syn*-**C**; (b) from *anti*-**C** using M06-2X/6-311+G**//M06-2X/6-31G*.

(a) Annulation from *syn*-**C**.

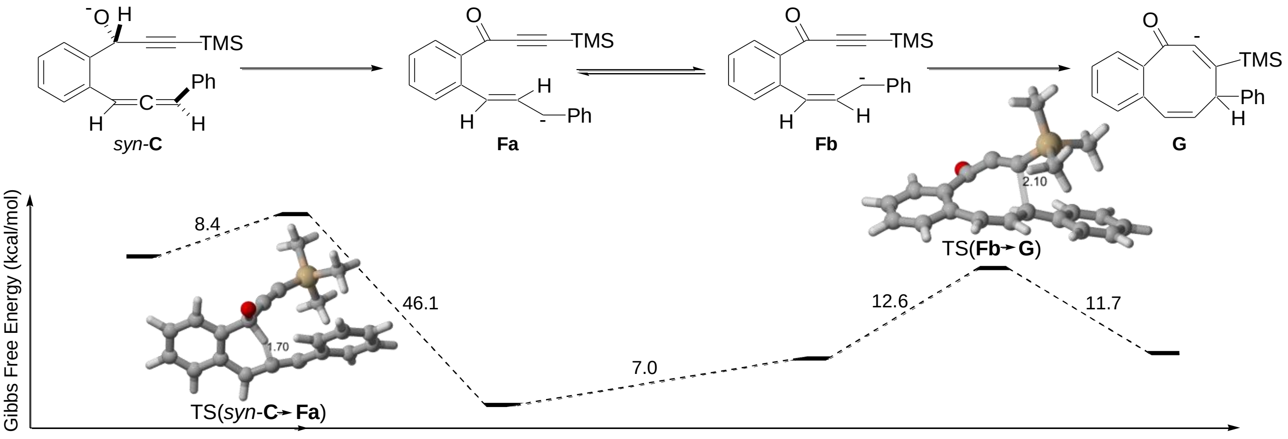

(b) Annulation from *anti*-**C**.

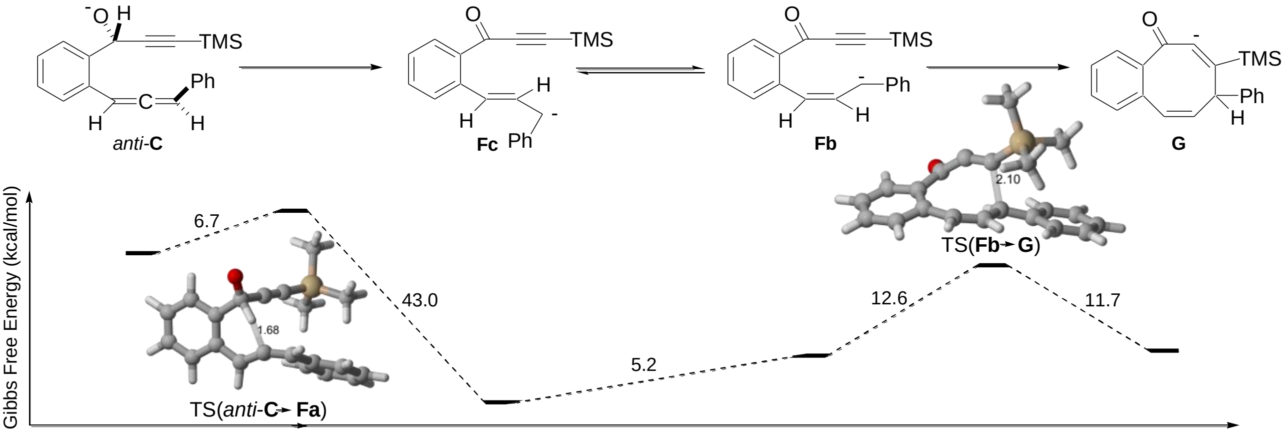

M06-2X/6-31G* Coordinates for Optimised Structures:

(i) ***syn*-C'**

Sum of electronic and zero-point Energies= -768.257102

Sum of electronic and thermal Energies= -768.240860

Sum of electronic and thermal Enthalpies= -768.239916

Sum of electronic and thermal Free Energies= -768.302446

H	3.54564600	-0.91894100	-2.13223900
C	3.11493100	0.07938100	-2.08766400
C	1.86303700	2.52613200	-1.82550400
C	1.82115200	0.11356200	-1.57167600
C	3.77656600	1.23590200	-2.48375300
C	3.14636800	2.47361100	-2.35276000
C	1.18451400	1.36101400	-1.43007800
H	4.78424900	1.18102500	-2.89105000
H	3.65019500	3.38962400	-2.65177400
H	1.36580800	3.48843400	-1.70949100
C	-0.16669000	1.56014600	-0.86990200
H	-0.49785700	2.60226300	-0.86272800
C	-1.02510400	0.69960200	-0.37579000
C	-1.95304300	-0.07070400	0.14293600
C	1.24803200	-1.28813100	-1.16018700
O	2.13131400	-2.25577900	-1.18937100
H	0.76287100	-1.08235900	-0.15678300
C	0.05690800	-1.51605600	-2.09514900
H	-1.62563000	-2.03826900	-3.53898600
C	-0.84756500	-1.78804600	-2.85468400
C	-2.03420400	-0.43127500	1.56853200
C	-2.24064600	-1.11855300	4.28613900
C	-3.17266600	-1.07899500	2.06148600
C	-0.99108300	-0.14485000	2.46057000
C	-1.09621500	-0.48321100	3.80310800
C	-3.27756800	-1.41795700	3.40707300

H	-3.98109700	-1.31801300	1.37473400
H	-0.09293200	0.33098100	2.07659300
H	-0.27381100	-0.26066000	4.47724800
H	-4.17045600	-1.92129400	3.76819000
H	-2.31718500	-1.38641700	5.33602800
H	-2.68783900	-0.52216500	-0.52306500

(ii) TS(*syn*-C'→F'a)

Sum of electronic and zero-point Energies= -768.249188

Sum of electronic and thermal Energies= -768.233528

Sum of electronic and thermal Enthalpies= -768.232584

Sum of electronic and thermal Free Energies= -768.293495

H	3.67700900	-1.08752400	-1.33943200
C	3.30746800	-0.09264100	-1.57421200
C	2.29256400	2.45378100	-1.99332900
C	1.98220600	0.17562400	-1.22885300
C	4.09796600	0.86907400	-2.18640100
C	3.58206100	2.15352800	-2.40004700
C	1.46994600	1.47489100	-1.40416800
H	5.11428000	0.63329100	-2.49304700
H	4.19740400	2.92088000	-2.86366000
H	1.90383500	3.46379700	-2.11377500
C	0.17081900	1.85662700	-0.85545600
H	-0.01905000	2.92786300	-0.76770900
C	-0.71014800	1.02506200	-0.29819200
C	-1.92163700	0.61711100	0.08378000
C	1.18356400	-0.93083300	-0.53536000

O	1.83121900	-1.68713700	0.29158700
H	0.32941000	-0.24570600	-0.03896500
C	0.33289900	-1.62515400	-1.57417200
H	-0.98690700	-2.73919200	-3.05574200
C	-0.37234700	-2.21524600	-2.36033900
C	-2.21696700	-0.12798500	1.30963300
C	-2.89137800	-1.55889100	3.64600600
C	-3.54672000	-0.21505400	1.75508700
C	-1.22532400	-0.79481800	2.05548500
C	-1.57048200	-1.49965900	3.20353800
C	-3.88173500	-0.91400300	2.90807900
H	-4.32370600	0.28438800	1.17991100
H	-0.19120900	-0.81353800	1.71761600
H	-0.79085700	-2.02004300	3.75295400
H	-4.91942000	-0.95724500	3.23027100
H	-3.14719700	-2.11123000	4.54602500
H	-2.75757000	0.77997900	-0.59711600

(iii) F'a

Sum of electronic and zero-point Energies= -768.324196

Sum of electronic and thermal Energies= -768.308016

Sum of electronic and thermal Enthalpies= -768.307072

Sum of electronic and thermal Free Energies= -768.369533

H	4.60270800	-0.06252700	-1.06885900
C	3.88710000	0.71227600	-1.32862800
C	1.98907900	2.58254900	-2.07515900
C	2.51850900	0.38731700	-1.17382000

C	4.30473400	1.91090600	-1.85634500
C	3.31843900	2.83950200	-2.27207000
C	1.51482800	1.39318800	-1.41998200
H	5.36027500	2.12268700	-1.99709900
H	3.61962600	3.77427100	-2.74132500
H	1.24614300	3.32528300	-2.35964500
C	0.16275900	1.34192300	-1.03909700
H	-0.48970600	2.09863900	-1.47572300
C	-0.40446300	0.50161400	-0.06516500
C	-1.73542900	0.40696600	0.25117600
C	2.26903900	-1.03114000	-0.91098000
O	3.10193400	-1.79214000	-0.41471200
H	0.29742000	-0.10252600	0.51141000
C	1.02106300	-1.63649900	-1.40608300
H	-0.84368300	-2.70499200	-2.14409000
C	0.04774300	-2.22844000	-1.80496000
C	-2.31157600	-0.41642300	1.28856300
C	-3.54286600	-2.00169300	3.31239300
C	-1.58197900	-1.37627500	2.02974300
C	-3.68584100	-0.30220300	1.59883400
C	-4.28604800	-1.07291000	2.58487800
C	-2.18481700	-2.14168600	3.01613000
H	-0.53081800	-1.53374400	1.80671000
H	-4.28134400	0.42014400	1.04402400
H	-5.34712900	-0.94599100	2.78962500
H	-1.58601300	-2.86930400	3.55939900
H	-4.00887400	-2.60618300	4.08500000
H	-2.44081100	1.00741200	-0.32414300

(iv) F'b

Sum of electronic and zero-point Energies= -768.313803

Sum of electronic and thermal Energies= -768.297543

Sum of electronic and thermal Enthalpies= -768.296599

Sum of electronic and thermal Free Energies= -768.359326

H	3.65817700	-2.11190500	-1.07363300
C	3.61562300	-1.06033200	-0.80473500
C	3.47154800	1.56682700	0.03593400
C	2.48377700	-0.64121600	-0.06558400
C	4.65355300	-0.21083100	-1.10383500
C	4.58380700	1.12024300	-0.62584100
C	2.31109600	0.74843800	0.26191100
H	5.52552600	-0.56132500	-1.64768600
H	5.41453400	1.80198500	-0.79813500
H	3.41618800	2.60658900	0.35300400
C	1.68261400	-1.74183200	0.46856500
O	1.65558600	-2.87382200	-0.01487900
C	-0.92144600	0.09657500	-0.03648800
C	1.14511300	1.40966700	0.71072900
H	1.34178400	2.41430300	1.08338600
C	-0.23020000	1.13771600	0.53262300
H	-0.85115300	1.96756200	0.87739700
H	-0.39366300	-0.79168600	-0.37075800
C	-2.34886000	0.06948000	-0.26428600
C	-5.15540500	-0.12727300	-0.75583100
C	-2.93460400	-1.08245200	-0.84109900

C	-3.24052000	1.12311800	0.05070200
C	-4.60358600	1.02157300	-0.18646300
C	-4.29716900	-1.17825400	-1.08135200
H	-2.28304400	-1.91544500	-1.09480400
H	-2.85367300	2.04012400	0.48494600
H	-5.24895500	1.85837000	0.07353900
H	-4.69643000	-2.08716100	-1.52643400
H	-6.22302400	-0.19956600	-0.94169300
C	0.99003300	-1.53773600	1.74855300
H	-0.09145100	-1.38264100	3.74013100
C	0.42422600	-1.47909300	2.81211500

(v) TS(F'b→G)

Sum of electronic and zero-point Energies= -768.300993

Sum of electronic and thermal Energies= -768.286059

Sum of electronic and thermal Enthalpies= -768.285115

Sum of electronic and thermal Free Energies= -768.343939

H	4.11841400	-2.20036300	-0.34879400
C	3.96851500	-1.12616400	-0.31623500
C	3.49303100	1.56581600	-0.17574400
C	2.68385300	-0.69542300	0.08814000
C	4.99489200	-0.25841700	-0.61159200
C	4.74769900	1.12183900	-0.51415900
C	2.38064800	0.70414200	0.08942700
H	5.97756200	-0.63137900	-0.88693200
H	5.53824800	1.84076000	-0.71782400
H	3.30898900	2.63739300	-0.14150400

C	1.80847700	-1.83276500	0.50532200
O	2.02156600	-2.98172000	0.09107400
C	0.78299400	-1.54427800	1.45059400
C	-0.32031800	-1.05791700	1.71917200
C	-0.93298200	-0.00161400	-0.14719000
C	1.14289700	1.42861600	0.28870700
H	1.33997800	2.48286000	0.48053600
C	-0.20793900	1.16054900	0.18001000
H	-0.82629300	2.03260300	0.40519200
H	-0.43309300	-0.79476500	-0.69319300
C	-2.38131300	-0.01256900	-0.22283800
C	-5.22350400	-0.14358100	-0.34439300
C	-3.05122000	-0.96593400	-1.02257400
C	-3.19973900	0.85175800	0.53985300
C	-4.58589400	0.79169900	0.46966500
C	-4.43494600	-1.02730800	-1.08463300
H	-2.45191400	-1.66193500	-1.60462800
H	-2.73289100	1.56885200	1.20989800
H	-5.17837400	1.47793300	1.07118200
H	-4.90762800	-1.77308200	-1.71994300
H	-6.30742000	-0.19168800	-0.39228700
H	-1.13229800	-0.76391400	2.35569400

(vi) G

Sum of electronic and zero-point Energies= -768.326614

Sum of electronic and thermal Energies= -768.312030

Sum of electronic and thermal Enthalpies= -768.311085

Sum of electronic and thermal Free Energies= -768.369318

H	3.99731100	-1.96643600	0.06385500
C	3.83950600	-0.89501700	-0.01432700
C	3.32123600	1.77949600	-0.29416900
C	2.50825900	-0.46253300	0.08908000
C	4.89709500	-0.02390400	-0.20898900
C	4.63155300	1.34014200	-0.34489500
C	2.21939500	0.91419100	-0.08905200
H	5.91778600	-0.39588100	-0.25735000
H	5.43824800	2.05193100	-0.50181000
H	3.12051000	2.84029000	-0.43098400
C	1.50729100	-1.59463100	0.31215000
O	1.73157000	-2.66577000	-0.29335900
C	0.57795100	-1.33676900	1.32127200
C	-0.57340200	-0.70038300	1.27812500
C	-1.05268200	-0.06501800	-0.05870300
C	0.91869400	1.61480600	-0.14683900
H	1.07561700	2.69107600	-0.23204500
C	-0.38585900	1.27914900	-0.17292300
H	-1.06675500	2.12864200	-0.25356400
H	-0.69800500	-0.72893200	-0.85474700
C	-2.55415000	0.03948700	-0.14320800
C	-5.36954200	0.16851900	-0.21067400
C	-3.29280700	-0.76750500	-1.01327700
C	-3.25931900	0.90576900	0.70255000
C	-4.64898900	0.97200500	0.67056800
C	-4.68305500	-0.70413200	-1.05190800
H	-2.76145100	-1.45639900	-1.66525500

H	-2.70094500	1.53051500	1.39647000
H	-5.17263800	1.65165500	1.33821200
H	-5.23278400	-1.33934600	-1.74178000
H	-6.45453600	0.21935200	-0.23850000
H	-1.20238900	-0.45748000	2.14176800

(vii) *anti*-C'

Sum of electronic and zero-point Energies= -768.257723

Sum of electronic and thermal Energies= -768.241625

Sum of electronic and thermal Enthalpies= -768.240681

Sum of electronic and thermal Free Energies= -768.302135

H	4.31361300	-1.01840300	-0.62531900
C	3.89122700	-0.01629900	-0.58741900
C	2.68579600	2.45061100	-0.29739100
C	2.62793100	0.04332600	-0.00129600
C	4.53887700	1.12311700	-1.04829700
C	3.93268000	2.37180300	-0.90157200
C	2.01365800	1.29990700	0.14994300
H	5.51999500	1.04826200	-1.51314900
H	4.43029600	3.27594500	-1.24382800
H	2.21166100	3.42199200	-0.16178200
C	0.70695500	1.52792900	0.79634300
H	0.47459000	2.58385000	0.96344000
C	-0.21436800	0.69436600	1.22079800
C	-1.23912600	-0.00180600	1.65899100
H	-1.22795100	-0.37358100	2.68372100
C	-2.42126900	-0.34470700	0.85188200

C	-4.72315700	-0.97072700	-0.63428900
C	-3.51710700	-0.97219800	1.45425400
C	-2.49350200	-0.04193800	-0.51560200
C	-3.63274100	-0.35070700	-1.24666900
C	-4.65845000	-1.28280800	0.72034600
H	-3.46674500	-1.21999200	2.51200400
H	-1.63000100	0.41159200	-0.99289400
H	-3.66886100	-0.11397900	-2.30667200
H	-5.49702900	-1.77221100	1.20846600
H	-5.61107200	-1.21355600	-1.21114000
C	2.07047200	-1.33716000	0.48677800
O	2.96331000	-2.29707700	0.50544500
H	1.58487300	-1.08288500	1.47747700
C	0.87879000	-1.62233600	-0.42963000
H	-0.82101600	-2.26430500	-1.80417700
C	-0.02232900	-1.95602400	-1.16876000

(viii) TS(*anti*-C'→F'c)

Sum of electronic and zero-point Energies= -768.250546

Sum of electronic and thermal Energies= -768.235020

Sum of electronic and thermal Enthalpies= -768.234076

Sum of electronic and thermal Free Energies= -768.294109

H	3.63622700	-1.13978000	-1.44276200
C	3.32073200	-0.11100900	-1.59384300
C	2.41365400	2.50279600	-1.82973300
C	1.99743400	0.18390900	-1.26240200
C	4.16531100	0.86864400	-2.09505000
C	3.70500400	2.18630500	-2.21770000

C	1.54089800	1.51145100	-1.34381100
H	5.18151500	0.61965700	-2.39184800
H	4.36372200	2.96342500	-2.59772600
H	2.06340000	3.53252200	-1.88196200
C	0.22986700	1.88036900	-0.83137500
H	-0.00965700	2.94571900	-0.81736700
C	-0.64141800	1.05304100	-0.24554300
C	-1.90176100	0.72442400	0.06506600
C	1.12874800	-0.94493700	-0.71045200
O	1.73043100	-1.92136400	-0.13211200
H	0.39338200	-0.23912800	0.01424700
C	0.08869900	-1.30301800	-1.74669600
H	-1.55718700	-1.89697500	-3.20200400
C	-0.78275700	-1.62495900	-2.52176300
C	-3.02172400	0.80813800	-0.86969000
C	-4.32985300	0.56150900	-0.41907200
C	-2.85251200	1.13145200	-2.22940500
C	-5.42101600	0.65011700	-1.27652500
H	-4.48198600	0.29814100	0.62535100
C	-3.94307000	1.21692100	-3.08258800
H	-1.84430400	1.28730100	-2.60244100
C	-5.23880600	0.98232600	-2.61654500
H	-6.42048800	0.45580900	-0.89461100
H	-3.78150500	1.46239700	-4.12944900
H	-6.08883000	1.05093500	-3.28940300
H	-2.12075000	0.30027800	1.04348400

(ix) F'c

Sum of electronic and zero-point Energies= -768.322001

Sum of electronic and thermal Energies= -768.306047

Sum of electronic and thermal Enthalpies= -768.305103

Sum of electronic and thermal Free Energies= -768.366665

H	4.87499100	-1.30691200	0.40108800
C	4.26718300	-0.43243600	0.18700800
C	2.64035300	1.71335300	-0.46557700
C	2.86412600	-0.61649200	0.19695500
C	4.84697500	0.76627400	-0.15450500
C	4.00162100	1.84165000	-0.52530100
C	1.99666000	0.51550900	-0.00191600
H	5.92690800	0.87504200	-0.18763400
H	4.43789300	2.78498300	-0.84831100
H	2.00327200	2.56085100	-0.71111400
C	0.61650000	0.54839200	0.26505800
H	0.06604900	1.40096000	-0.12856000
C	-0.05046900	-0.31705000	1.15543700
C	-1.39215000	-0.48064300	1.40055300
H	-1.66796200	-1.05489600	2.28322600
C	-2.50895300	-0.00181900	0.61427700
C	-4.78410000	0.85400100	-0.87049200
C	-3.80948900	-0.00177500	1.16892600
C	-2.40159500	0.42032400	-0.73149500
C	-3.51464100	0.83806400	-1.44946100
C	-4.91793900	0.41910500	0.44878400
H	-3.93140000	-0.33882900	2.19646800
H	-1.43164400	0.37570300	-1.21539800

H	-3.39010900	1.14832000	-2.48477000
H	-5.89831000	0.40936700	0.92056700
H	-5.64910900	1.18917900	-1.43562000
C	2.43941700	-2.01780200	0.25356000
O	3.14202900	-2.92744500	0.69899800
H	0.61387900	-0.91725600	1.78638200
C	1.17816100	-2.39440700	-0.40400300
H	-0.74271700	-3.06846500	-1.41370800
C	0.17457300	-2.78484900	-0.94942500

(x) *syn*-C

Sum of electronic and zero-point Energies= -1176.745335

Sum of electronic and thermal Energies= -1176.721077

Sum of electronic and thermal Enthalpies= -1176.720133

Sum of electronic and thermal Free Energies= -1176.803406

H	3.27690600	-0.25061200	-1.59530200
C	2.73159100	0.68221300	-1.72249300
C	1.18170500	2.95919300	-1.91484100
C	1.38075800	0.59972400	-1.39310500
C	3.30757400	1.87108000	-2.15518700
C	2.52680400	3.02262100	-2.25324200
C	0.58923400	1.76033300	-1.48413100
H	4.36392300	1.90808300	-2.41334200
H	2.96072200	3.96279800	-2.58458600
H	0.56599900	3.85528100	-1.97969200
C	-0.84530800	1.83690000	-1.14287200
H	-1.28426600	2.82805600	-1.28601900

C	-1.67212500	0.92145800	-0.69505000
C	-2.57881500	0.08472500	-0.24645000
C	0.91945500	-0.81611000	-0.90114000
O	1.89694400	-1.66824800	-0.71235200
H	0.27285400	-0.58005300	-0.00028000
C	-0.09385300	-1.28452400	-1.94143000
C	-0.86589700	-1.74964100	-2.76796300
C	-2.84487400	-0.17754200	1.17807200
C	-3.40213200	-0.69023000	3.88375200
C	-3.94389500	-0.96032500	1.54870700
C	-2.01902600	0.33548100	2.18809500
C	-2.29736600	0.08248800	3.52458600
C	-4.22252200	-1.21346100	2.88838800
H	-4.58128300	-1.37506000	0.77134700
H	-1.14881400	0.92069800	1.90377900
H	-1.64154500	0.48177700	4.29315700
H	-5.08042400	-1.82510500	3.15415900
H	-3.61410800	-0.89029000	4.93008000
H	-3.15050600	-0.49968700	-0.96810500
Si	-2.09013100	-2.35353200	-3.94703700
C	-1.28165500	-3.26360600	-5.39060600
H	-0.70733400	-4.12342200	-5.03212200
H	-2.03235100	-3.62415300	-6.10369900
H	-0.59133800	-2.60290800	-5.92424600
C	-3.29879800	-3.54062600	-3.10855100
H	-2.76910200	-4.40741100	-2.70136300
H	-3.81200300	-3.04719200	-2.27628900
H	-4.05808600	-3.90052200	-3.81287700

C	-3.09003600	-0.91122300	-4.64930000
H	-3.58064700	-0.35341100	-3.84457500
H	-2.43595300	-0.21301400	-5.18094400
H	-3.86188500	-1.25861500	-5.34621300

(xi) TS(*syn*-C→Fa)

Sum of electronic and zero-point Energies= -1176.737024

Sum of electronic and thermal Energies= -1176.713663

Sum of electronic and thermal Enthalpies= -1176.712719

Sum of electronic and thermal Free Energies= -1176.790538

H	3.89009200	-1.85273200	-0.68410400
C	3.81611500	-0.85509800	-1.10890700
C	3.53536100	1.75063000	-2.01219000
C	2.54879500	-0.26742500	-1.09596200
C	4.91318500	-0.18944100	-1.63329600
C	4.76870600	1.12758900	-2.08917500
C	2.40363700	1.06853800	-1.52256600
H	5.88303300	-0.67857700	-1.68158500
H	5.62602800	1.66800700	-2.48319900
H	3.42765800	2.78831700	-2.32374900
C	1.14576900	1.79040900	-1.34990200
H	1.19676000	2.87978700	-1.37874900
C	-0.00711100	1.21026000	-1.01162100
C	-1.31587100	0.97992000	-1.08216600
C	1.41687700	-1.05123600	-0.42748100
O	1.73317000	-1.75788800	0.60971300
H	0.66778100	-0.13120800	-0.22468300

C	0.53515200	-1.72762900	-1.44574300
C	-0.33836900	-2.24688700	-2.12299300
C	-2.16602100	0.31860700	-0.08656000
C	-3.90891000	-1.00166400	1.69686300
C	-3.56043600	0.37233600	-0.25199300
C	-1.65767400	-0.42586400	0.99509500
C	-2.52624000	-1.07754900	1.86380000
C	-4.42288900	-0.27199600	0.62755200
H	-3.96514300	0.93392600	-1.09184100
H	-0.58676400	-0.54738500	1.13771300
H	-2.11007800	-1.66452900	2.67759800
H	-5.49705800	-0.20933500	0.47282500
H	-4.57565600	-1.51569200	2.38343700
H	-1.80314900	1.21343400	-2.03054900
Si	-1.89223200	-2.84059600	-2.84051700
C	-3.02827300	-3.43240200	-1.45958600
H	-2.57739200	-4.26571700	-0.91172300
H	-3.19569800	-2.61682500	-0.74658600
H	-4.00032200	-3.75853400	-1.84861800
C	-1.59897400	-4.25569300	-4.05900700
H	-2.54556300	-4.61931700	-4.47561200
H	-0.96412600	-3.93041300	-4.88950000
H	-1.09927000	-5.09538100	-3.56568700
C	-2.76408500	-1.43836800	-3.75545100
H	-2.10641000	-0.97512600	-4.49744600
H	-3.66570400	-1.79682100	-4.26607000
H	-3.06197300	-0.66706800	-3.03726900

(xii) Fa

Sum of electronic and zero-point Energies= -1176.814886

Sum of electronic and thermal Energies= -1176.791066

Sum of electronic and thermal Enthalpies= -1176.790122

Sum of electronic and thermal Free Energies= -1176.869022

H	5.63344100	-3.17215700	-2.37359400
C	5.29926200	-2.15666700	-2.18269300
C	4.32709500	0.39896400	-1.77197500
C	3.97547900	-2.00557300	-1.70632000
C	6.10784900	-1.08207000	-2.46888700
C	5.58118000	0.21955100	-2.29060900
C	3.49164200	-0.69402300	-1.35846700
H	7.10845300	-1.22610000	-2.86500400
H	6.18182400	1.08783700	-2.55450000
H	3.95181800	1.40579400	-1.59959800
C	2.32447800	-0.39064800	-0.62881500
H	1.99585100	0.64857100	-0.65460200
C	1.59973400	-1.27010900	0.18655300
C	0.37651200	-1.03120400	0.76380700
C	3.18366400	-3.24152800	-1.75741200
O	3.68679000	-4.36688900	-1.77179700
H	2.06108000	-2.24182600	0.36651400
C	1.72903600	-3.15651600	-1.94455100
C	0.52071900	-3.18055600	-2.09150000
C	-0.37662500	-1.95625600	1.57452300
C	-1.99062500	-3.75440400	3.09135100
C	0.10081100	-3.22677600	1.98038400

C	-1.69324000	-1.62882700	1.97701900
C	-2.47998200	-2.50488100	2.71238600
C	-0.68896000	-4.09749400	2.71539500
H	1.10294000	-3.53487600	1.69825000
H	-2.09508300	-0.65952500	1.68698800
H	-3.48944200	-2.20967200	2.99047100
H	-0.28397600	-5.06692300	2.99655100
H	-2.60441300	-4.44361100	3.66330900
H	-0.11748500	-0.08547500	0.53818900
Si	-1.30655900	-3.09763800	-2.20324900
C	-1.81395500	-3.66945700	-3.93203100
H	-1.46911100	-4.69012700	-4.12490200
H	-2.90446500	-3.65106200	-4.04214800
H	-1.38390800	-3.01932300	-4.70054000
C	-2.08970100	-4.22823200	-0.92311400
H	-1.84132500	-3.89649700	0.09065000
H	-3.18120100	-4.22041000	-1.02744100
H	-1.74209100	-5.25952100	-1.04009500
C	-1.90068100	-1.33175800	-1.94828700
H	-1.86580700	-1.08380800	-0.88396700
H	-1.26045700	-0.62183100	-2.48055100
H	-2.93048700	-1.21260000	-2.30490000

(xiii) Fb

Sum of electronic and zero-point Energies= -1176.803788

Sum of electronic and thermal Energies= -1176.780119

Sum of electronic and thermal Enthalpies= -1176.779175

Sum of electronic and thermal Free Energies= -1176.857381

H	2.90178100	-0.25726800	-1.77411200
C	2.85893700	0.65541500	-1.18783500
C	2.73994400	2.87957500	0.42874000
C	1.87622900	0.69902000	-0.16695500
C	3.76348500	1.66764600	-1.39151100
C	3.71666500	2.78464700	-0.52521800
C	1.70054100	1.90032800	0.60589300
H	4.52220900	1.59327100	-2.16477500
H	4.44981400	3.58262600	-0.62386700
H	2.69280000	3.76836600	1.05459300
C	1.25537700	-0.60598700	0.08761000
O	1.26832800	-1.53782100	-0.72084800
C	-1.54608400	1.09379200	0.91991800
C	0.64728200	2.29682700	1.47414500
H	0.91802200	3.16680200	2.07137400
C	-0.72446700	1.98848300	1.56567400
H	-1.22847900	2.63579300	2.28690600
H	-1.12784700	0.39832400	0.19952700
C	-2.96989100	0.97091800	1.12692000
C	-5.77010700	0.55222300	1.50982400
C	-3.68596800	-0.03153100	0.42740300
C	-3.73246300	1.76496300	2.01921800
C	-5.09147400	1.55335900	2.20567300
C	-5.04517400	-0.23498900	0.61326100
H	-3.13545300	-0.66488100	-0.26445000
H	-3.24848600	2.56498300	2.57216600
H	-5.63274500	2.18589100	2.90615400

H	-5.54616600	-1.02273700	0.05495000
H	-6.83334300	0.39148000	1.66054700
C	0.69449100	-0.85502700	1.41026700
C	0.12260800	-1.07857600	2.46033900
Si	-0.90365200	-1.13639500	3.97687200
C	-1.19250100	0.60919500	4.61354400
H	-0.30416400	1.22848600	4.45672300
H	-2.02039300	1.06540300	4.06100300
H	-1.44448600	0.60478200	5.68031400
C	-2.55022800	-1.95705700	3.59312900
H	-3.17358700	-2.01052200	4.49330600
H	-3.09188700	-1.37769100	2.83692300
H	-2.41042800	-2.97312000	3.21095300
C	0.01291800	-2.14053200	5.28909000
H	-0.57979300	-2.20875200	6.20859300
H	0.21443900	-3.15742600	4.93787400
H	0.97166000	-1.67434400	5.53741800

(xiv) TS(Fb→G)

Sum of electronic and zero-point Energies= -1176.785903

Sum of electronic and thermal Energies= -1176.763379

Sum of electronic and thermal Enthalpies= -1176.762434

Sum of electronic and thermal Free Energies= -1176.837074

H	4.61732700	-3.12124500	2.77970000
C	4.56869700	-2.10237100	2.40909900
C	4.33410400	0.45538500	1.45853500
C	3.28119300	-1.62631300	2.08181400

C	5.70276500	-1.32626400	2.30640900
C	5.57226500	-0.01098900	1.83563000
C	3.13986800	-0.32390800	1.51607600
H	6.67226100	-1.71760700	2.60273900
H	6.44217400	0.63660000	1.75422000
H	4.25369700	1.46490400	1.06129900
C	2.19963300	-2.62104600	2.38942900
O	2.45413300	-3.83799000	2.37508000
C	0.95216100	-2.08527300	2.78097300
C	-0.12713200	-1.54784700	2.41158800
C	0.09560700	-1.25119400	0.34364500
C	1.98752600	0.36033500	0.94620700
H	2.19127000	1.42464000	0.83153400
C	0.76959900	-0.00014700	0.42519200
H	0.19848400	0.85957100	0.07116500
H	0.70136900	-2.14949800	0.34145100
C	-1.16429800	-1.41440300	-0.38278700
C	-3.68307500	-1.89717500	-1.61638100
C	-1.66876900	-2.72117600	-0.56381000
C	-1.96790600	-0.35997000	-0.86347800
C	-3.20081300	-0.59913600	-1.46196200
C	-2.89525500	-2.95848100	-1.16447600
H	-1.07623700	-3.55360000	-0.19118600
H	-1.62319100	0.66589000	-0.77599900
H	-3.79064300	0.24330600	-1.81612200
H	-3.24732400	-3.98131300	-1.27502100
H	-4.64790900	-2.07929600	-2.08037600
Si	-1.66093000	-0.73880200	3.02736300

C	-1.62079900	-0.80804700	4.91570600
H	-0.73235700	-0.29419600	5.29556100
H	-2.50917900	-0.33811300	5.35417500
H	-1.57308700	-1.84599500	5.25940500
C	-3.22924000	-1.60457700	2.43586300
H	-4.10169400	-1.21207700	2.97288900
H	-3.39139600	-1.45920900	1.36297000
H	-3.17130500	-2.68177800	2.62310200
C	-1.72197900	1.07118900	2.49670000
H	-2.48564500	1.61399700	3.06642600
H	-0.75216800	1.55083300	2.66134400
H	-1.96752100	1.15653800	1.43396200

(xv) G

Sum of electronic and zero-point Energies= -1176.802406

Sum of electronic and thermal Energies= -1176.779752

Sum of electronic and thermal Enthalpies= -1176.778808

Sum of electronic and thermal Free Energies= -1176.854802

H	2.88875100	-3.22723700	-0.70873400
C	2.38803600	-3.94494400	-0.06610200
C	1.16941800	-5.68106900	1.66725000
C	1.73412500	-3.41409900	1.05667500
C	2.40665600	-5.29902400	-0.34982200
C	1.77929900	-6.18162000	0.53187800
C	1.12954800	-4.30134000	1.98321900
H	2.90189200	-5.66938300	-1.24403100
H	1.77620300	-7.25164800	0.34016600

H	0.70715700	-6.37838100	2.36294700
C	1.84206500	-1.89590800	1.16734700
O	2.92566400	-1.37907000	0.82265800
C	0.63542400	-1.25470400	1.46986000
C	0.05975500	-1.01625000	2.64656400
C	0.80488400	-1.55906000	3.91890900
C	0.47944500	-4.02702300	3.28240000
H	0.02262000	-4.93551900	3.67628400
C	0.35531000	-2.97189500	4.11203500
H	-0.20574800	-3.19196200	5.01940900
H	1.86602400	-1.54859300	3.64498700
C	0.64352000	-0.63565600	5.10898900
C	0.34000000	1.30902700	7.13419900
C	1.33572600	0.58387400	5.05985600
C	-0.20302400	-0.85436500	6.19865000
C	-0.35493200	0.10717100	7.19943800
C	1.19236300	1.54191000	6.05376900
H	1.97629900	0.77409500	4.20143400
H	-0.76650200	-1.77849500	6.27863900
H	-1.02476300	-0.08949100	8.03269300
H	1.73977500	2.47812900	5.98400800
H	0.21889700	2.05788800	7.91190900
Si	-1.65918300	-0.31513600	2.73287400
C	-1.77495600	1.35950600	3.62206000
H	-2.78392200	1.78086600	3.52778200
H	-1.54148900	1.26850200	4.68892700
H	-1.06756900	2.07513400	3.18862800
C	-2.30906500	-0.07398900	0.97855300

H	-1.63543700	0.57977500	0.41686500
H	-2.33511500	-1.03227400	0.45156800
H	-3.31517500	0.36255900	0.97513400
C	-2.85028500	-1.48638000	3.64042400
H	-3.88181500	-1.11552100	3.59783900
H	-2.82197300	-2.48432600	3.19017600
H	-2.57628600	-1.58847800	4.69646400

(xvi) *anti*-C

Sum of electronic and zero-point Energies= -1176.747968

Sum of electronic and thermal Energies= -1176.724056

Sum of electronic and thermal Enthalpies= -1176.723112

Sum of electronic and thermal Free Energies= -1176.802090

H	4.83430600	-3.57357400	-3.61336400
C	4.97351600	-2.61007700	-3.12800200
C	5.28550300	-0.27831500	-1.68030600
C	4.23863400	-2.44931400	-1.95430400
C	5.82724400	-1.61937900	-3.59628000
C	5.98880400	-0.44107000	-2.86524400
C	4.39680900	-1.26316500	-1.21610300
H	6.38038600	-1.76307500	-4.52212400
H	6.66542700	0.33764600	-3.20834400
H	5.41954200	0.62812400	-1.09142500
C	3.73131500	-0.98344100	0.07077600
H	4.16261500	-0.14460200	0.62448400
C	2.72388500	-1.57804400	0.66603700
C	1.70108600	-2.03707400	1.35052700

H	1.85278900	-2.86450000	2.04350900
C	0.33579200	-1.49178400	1.26556800
C	-2.23887400	-0.36288700	1.25218700
C	-0.62297800	-1.87584000	2.21022900
C	-0.02701200	-0.55191700	0.28956800
C	-1.29703000	0.01184100	0.29296500
C	-1.89842400	-1.31785100	2.20582700
H	-0.35606700	-2.61623600	2.96056600
H	0.70103800	-0.28373800	-0.47147400
H	-1.56114900	0.74336400	-0.46577000
H	-2.62653500	-1.63101600	2.94909100
H	-3.23236000	0.07573800	1.24492000
C	3.36460000	-3.68518400	-1.55769300
O	3.66498100	-4.78511500	-2.20535100
H	3.45114900	-3.72194400	-0.43059100
C	1.92073300	-3.24473600	-1.79534900
C	0.74776200	-3.02871400	-2.06861300
Si	-0.98306200	-2.89700100	-2.57472000
C	-2.14843200	-3.30266700	-1.14765400
H	-1.94804500	-2.67303200	-0.27577600
H	-3.19584300	-3.16305000	-1.44157000
H	-2.01672300	-4.34705700	-0.84534800
C	-1.37355800	-1.17073700	-3.24057400
H	-0.77605700	-0.96795200	-4.13541700
H	-2.43294000	-1.08192600	-3.50912800
H	-1.14217300	-0.39892500	-2.50153700
C	-1.34553200	-4.11753300	-3.97188000
H	-1.14063400	-5.14264800	-3.64834400

H	-2.39333800	-4.05846300	-4.29056600
H	-0.70974600	-3.91237300	-4.83874900

(xvii) TS(*anti*-C→Fc)

Sum of electronic and zero-point Energies= -1176.741096

Sum of electronic and thermal Energies= -1176.717904

Sum of electronic and thermal Enthalpies= -1176.716960

Sum of electronic and thermal Free Energies= -1176.793596

H	3.75095900	-1.33122400	-0.67277700
C	3.55977400	-0.36123900	-1.12358100
C	2.95159200	2.15006900	-2.14093700
C	2.23940600	0.08966800	-1.08331600
C	4.55463400	0.40037100	-1.71810800
C	4.24546200	1.66574700	-2.23452000
C	1.92882900	1.37417600	-1.56357100
H	5.57187100	0.02177200	-1.78387600
H	5.02161400	2.27538900	-2.69053700
H	2.71392000	3.14954700	-2.50155200
C	0.60460100	1.94363900	-1.36506300
H	0.47777400	2.99563700	-1.62912600
C	-0.41891200	1.34197200	-0.75084600
C	-1.73385000	1.16782000	-0.57444600
C	1.20351600	-0.80139500	-0.40092500
O	1.62542100	-1.61644900	0.49826800
H	0.43572100	0.10269500	-0.01596000
C	0.29630900	-1.38245800	-1.45783500
C	-0.48227300	-1.88954400	-2.24938200
Si	-1.63054400	-2.67628700	-3.41504100

C	-1.26620300	-4.52849200	-3.51311500
H	-0.24004600	-4.70611800	-3.84992100
H	-1.37945100	-4.99749200	-2.53077000
H	-1.94798900	-5.02751900	-4.21198300
C	-1.41115600	-1.95572600	-5.14557400
H	-2.10276700	-2.42317900	-5.85631000
H	-1.60456800	-0.87925600	-5.13794100
H	-0.38939000	-2.11466200	-5.50472100
C	-3.41677300	-2.45282300	-2.86507000
H	-3.57353500	-2.90705800	-1.88127100
H	-3.67478300	-1.39186000	-2.79128900
H	-4.10547700	-2.92696200	-3.57471300
C	-2.70784300	1.12362100	-1.66269500
C	-4.08015100	1.05016900	-1.36669000
C	-2.33719500	1.16585700	-3.02028300
C	-5.04019600	1.04186900	-2.37271100
H	-4.38772700	1.00117400	-0.32461700
C	-3.29862600	1.15936000	-4.02209400
H	-1.27936900	1.18824400	-3.26919500
C	-4.65885600	1.10099700	-3.71110500
H	-6.09324100	0.98411500	-2.10858500
H	-2.98399300	1.19192900	-5.06245100
H	-5.40536600	1.09221500	-4.49970900
H	-2.12228600	0.99084200	0.42690100

(xviii) Fc

Sum of electronic and zero-point Energies= -1176.811513

Sum of electronic and thermal Energies= -1176.787775

Sum of electronic and thermal Enthalpies= -1176.786830

Sum of electronic and thermal Free Energies= -1176.866202

H	3.72862700	-3.74942300	3.17691300
C	3.29760900	-2.75505200	3.10436900
C	2.15597700	-0.25143100	2.77729500
C	1.97316700	-2.67020100	2.61540600
C	4.04079000	-1.63680800	3.40104700
C	3.45637200	-0.36385400	3.19098100
C	1.31367400	-1.39377500	2.56371100
H	5.06765100	-1.72392100	3.74316900
H	4.04130800	0.53560500	3.37287300
H	1.70548000	0.73230000	2.66109800
C	-0.06709100	-1.19424400	2.37422200
H	-0.38229800	-0.17711900	2.14956900
C	-1.06191500	-2.15241400	2.64533400
C	-2.40985600	-2.13637600	2.37406700
H	-3.02340000	-2.89180400	2.86165500
C	-3.12890000	-1.25129800	1.48542900
C	-4.65050700	0.36477100	-0.30368500
C	-4.54240400	-1.20745600	1.53016000
C	-2.51040900	-0.46216700	0.48622800
C	-3.25820100	0.32281000	-0.38304700
C	-5.28360800	-0.41880900	0.66216700
H	-5.05326200	-1.81748000	2.27246900
H	-1.43199200	-0.51159500	0.37550100
H	-2.74251400	0.90487900	-1.14423100
H	-6.36883600	-0.41508200	0.73808000

H	-5.22818800	0.98503800	-0.98263100
C	1.45214800	-3.92088100	2.05539800
O	1.87556100	-5.03568200	2.36759500
H	-0.71108100	-3.02714800	3.20304100
C	0.49386600	-3.85424500	0.94173000
C	-0.26863000	-3.90721200	-0.00634400
Si	-1.45156400	-3.99510400	-1.40124500
C	-1.28316300	-2.47498200	-2.49764700
H	-1.91365300	-2.57456800	-3.38886900
H	-1.60281400	-1.58118500	-1.95374400
H	-0.24814200	-2.33201200	-2.82307600
C	-3.21347400	-4.13510000	-0.76644700
H	-3.51807300	-3.20920000	-0.26880100
H	-3.90375300	-4.32722100	-1.59654200
H	-3.30507700	-4.95318200	-0.04534700
C	-1.03946700	-5.52732900	-2.42695800
H	-1.11954800	-6.43498000	-1.82069200
H	-1.72506400	-5.62269200	-3.27696600
H	-0.01802400	-5.47508400	-2.81674600

5. References

[S1] H. E. Gottlieb, V. Kotlyar, A. Nudelman, *J. Org. Chem.*, **1997**, *62*, 7512-7515.

[S2] T. S. N. Zhao, K. Szabó, *Org. Lett.* **2012**, *14*, 3966-3969.

[S3] Y. Matsuya, A. Koiwa, Azusa, D. Minato, Daishiro, K. Sugimoto, N. Toyooka, *Tetrahedron. Lett.*, **2012**, *53*, 5955-5957.

[S4] M. S. H. Siah, M. Kaur, N. Iqbal, A. Fiksdahl, *Eur. J. Org. Chem.* **2014**, 1727-1740.

[S5] F. C. Pigge, F. Ghasedi, Z. Zheng, N. P. Rath, G. Nichols, J. S. Chickos, *J. Chem. Soc., Perkin trans.*, **2000**,

2, 2458-2464.

[S6] R. U. Braun, M. Ansorge, T. J. J. Müller, *Chem. Eur. J.* **2006**, *12*, 9081-9094.

[S6a] B. W. Nash, D. A. Thomas, W. K. Warburton, T. D. Williams, *J. Chem. Soc.,* **1965**, 2983-2988.

[S7] K. C. Weerasiri, A. E. V. Gorden, *Eur. J. Org. Chem,* **2013**, 1546-1550.

[S8] Y. Maeda, N. Kakiuchi, S. Matsumura, T. Nishimura, T. Kawamura, S. Uemura, *J. Org. Chem.* **2002**, *67*, 6718-6724.

[S9] W. Lin, M. H. Hu, X. Feng, L. Fu, C. P. Cao, Z. B. Huang, D. E. Shi, *Tett. Lett.* **2014**, *55*, 2238-2242.

[S10] Y. Motoyama, K. Kamo, H. Nagashima, *Org. Lett.* **2009**, *11*, 1345-1348.

[S11] T. C. Gilchrist, P. D. Kemmitt, *Tetrahedron*, **1997**, *53*, 4447-4456.

[S12] C. B. De Koning, J. P. Michael, A. L. Rousseau, *J. Chem. Soc., Perkin Trans.,* **2000**, *1*, 1705-1713.

[S13] M. J. Frisch, G. W. Trucks, H. B. Schlegel, G. E. Scuseria, M. A. Robb, J. R. Cheeseman, G. Scalmani, V. Barone, B. Mennucci, G. A. Petersson, H. Nakatsuji, M. Caricato, X. Li, H. P. Hratchian, A. F. Izmaylov, J. Bloino, G. Zheng, J. L. Sonnenberg, M. Hada, M. Ehara, K. Toyota, R. Fukuda, J. Hasegawa, M. Ishida, T. Nakajima, Y. Honda, O. Kitao, H. Nakai, T. Vreven, J. A. Jr. Montgomery, J. E. Peralta, F. Ogliaro, M. Bearpark, J. J. Heyd, E. Brothers, K. N. Kudin, V. N. Staroverov, R. Kobayashi, J. Normand, K. Raghavachari, A. Rendell, J. C. Burant, S. S. Iyengar, J. Tomasi, M. Cossi, N. Rega, J. M. Millam, M. Klene, J. E. Knox, J. B. Cross, V. Bakken, C. Adamo, J. Jaramillo, R. Gomperts, R. E. Stratmann, O. Yazyev, A. J. Austin, R. Cammi, C. Pomelli, J. W. Ochterski, R. L. Martin, K. Morokuma, V. G. Zakrzewski, G. A. Voth, P. Salvador, J. J. Dannenberg, S. Dapprich, A. D. Daniels, Ö. Farkas, J. B. Foresman, J. V. Ortiz, J. Cioslowski, D. J. Fox, Gaussian 09, Gaussian, Inc., Wallingford, CT, **2009**.

[S14] C. Lee, W. Yang, R. G. Parr, *Phys. Rev. B* **1988**, *37*, 785-789; A. D. Becke, *J. Chem. Phys*. **1993**, *98*, 1372; A. D. Becke, *J. Chem. Phys.* **1993**, *98*, 5648-5652; P. J. Stephens, F. J. Devlin, C. F. Chabalowski, M. J. Frisch, *J. Phys. Chem*. **1994**, *98*, 11623-11627.

[S15] Y. Zhao, D. G. Truhlar, *Theor. Chem. Acc.* **2008**, *120*, 215-241.

[S16] J. A. Montgomery, M. J. Frisch, J. W. Ochterski, and G. A. Petersson, *J. Chem. Phys.*, **1999**, *110*, 2822-2827; J. A. Jr. Montgomery, M. J. Frisch, J. W. Ochterski, G. A. Petersson, *J. Chem. Phys.*, **2000**, *112* 6532-6542.

[S17] CYLview, 1.0b; C. Y. Legault, Université de Sherbrooke, **2009** (http://www.cylview.org)

6. Spectroscopic data

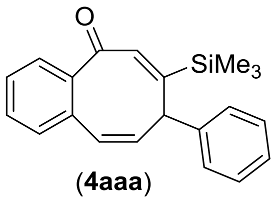

(**4aaa**)

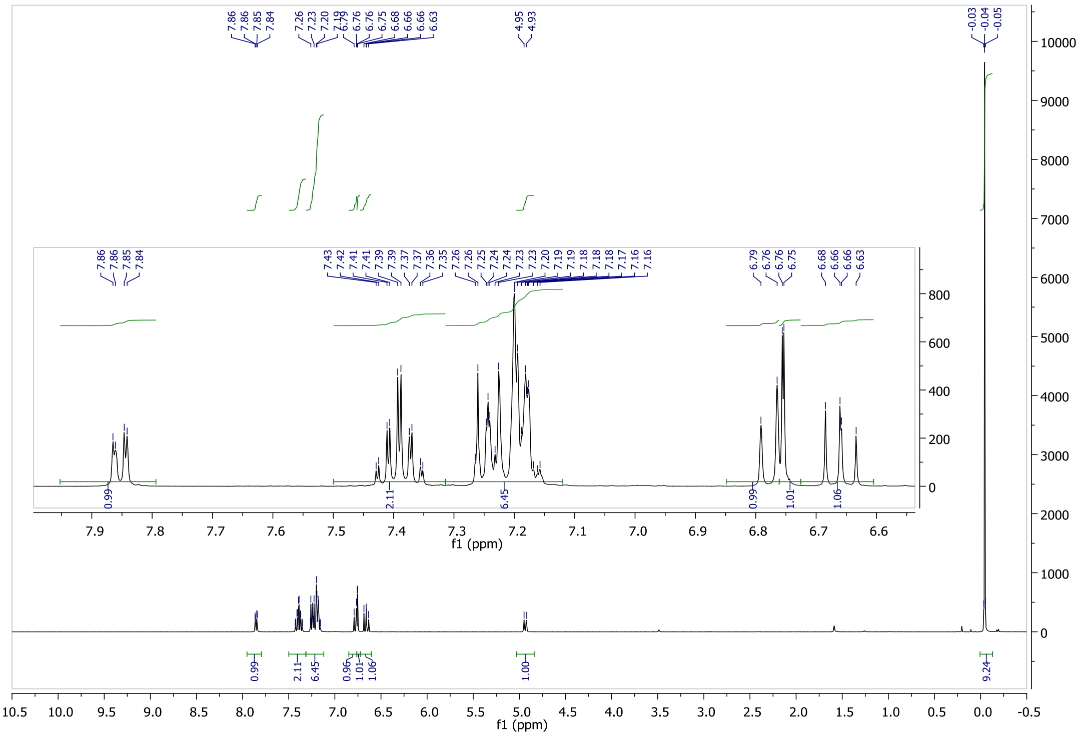

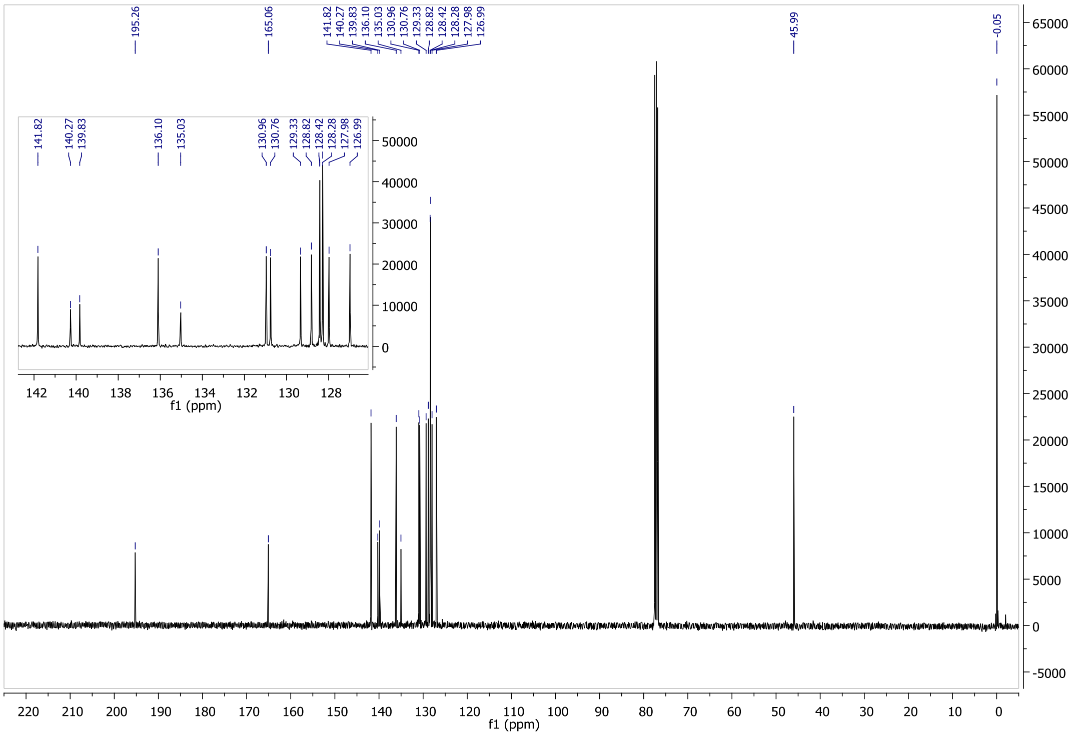

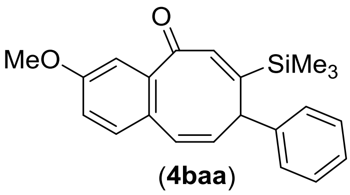

MeO
SiMe3

(**4baa**)

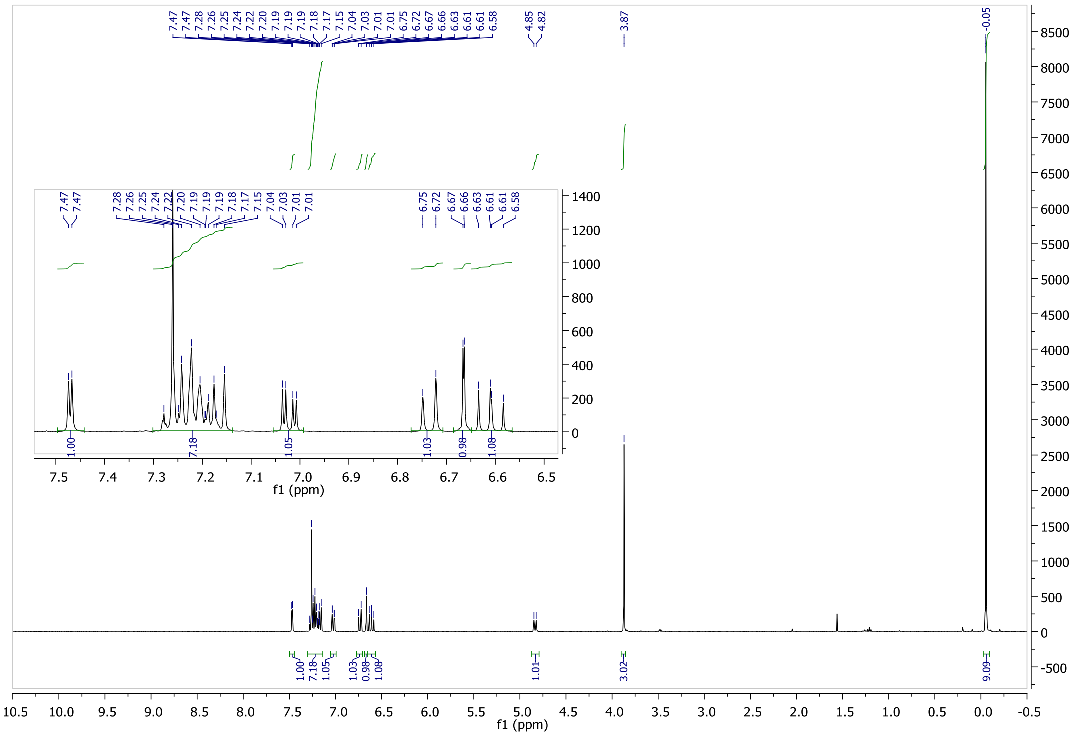

7.47
7.47
7.28
7.26
7.25
7.24
7.22
7.22
7.20
7.19
7.19
7.19
7.18
7.17
7.15
7.04
7.03
7.01
7.01
6.75
6.72
6.67
6.66
6.63
6.61
6.61
6.58
4.85
4.82
3.87
-0.05
1.00
7.18
1.05
1.03
0.98
1.08
1.01
3.02
9.09
f1 (ppm)

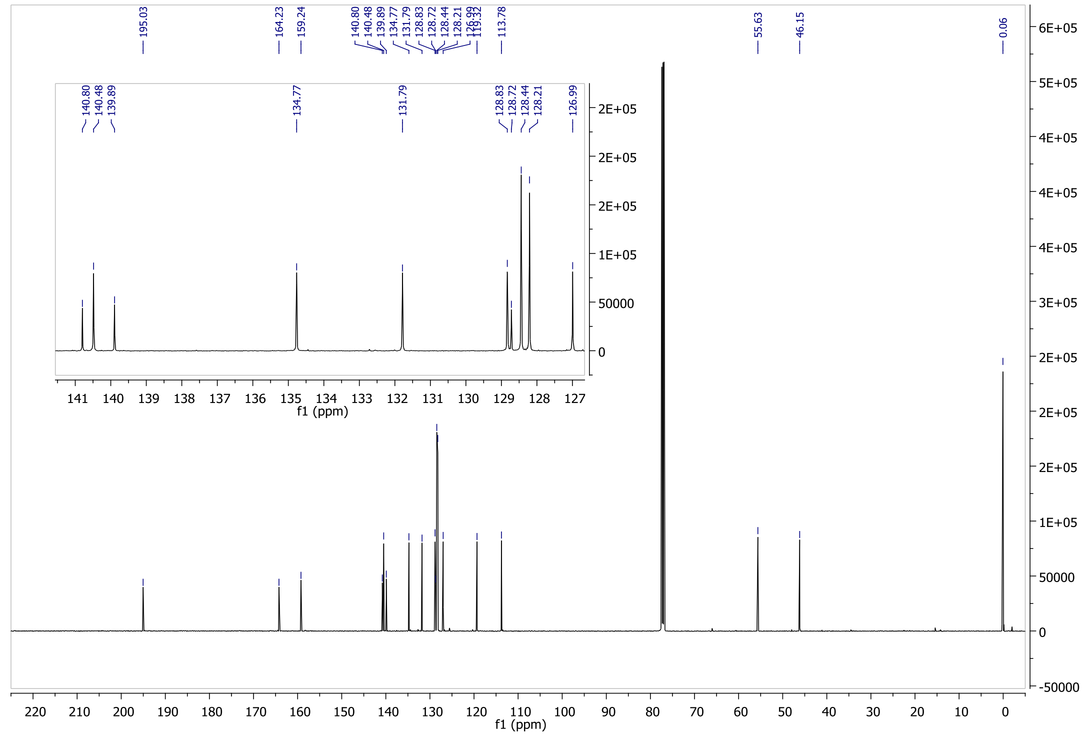

195.03
164.23
159.24
140.80
140.48
139.89
134.77
131.79
128.83
128.72
128.44
128.21
126.99
119.32
113.78
55.63
46.15
0.06
f1 (ppm)

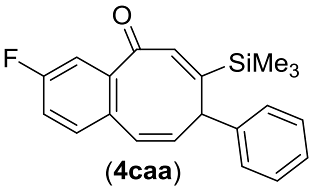
O
F
SiMe3

(**4caa**)

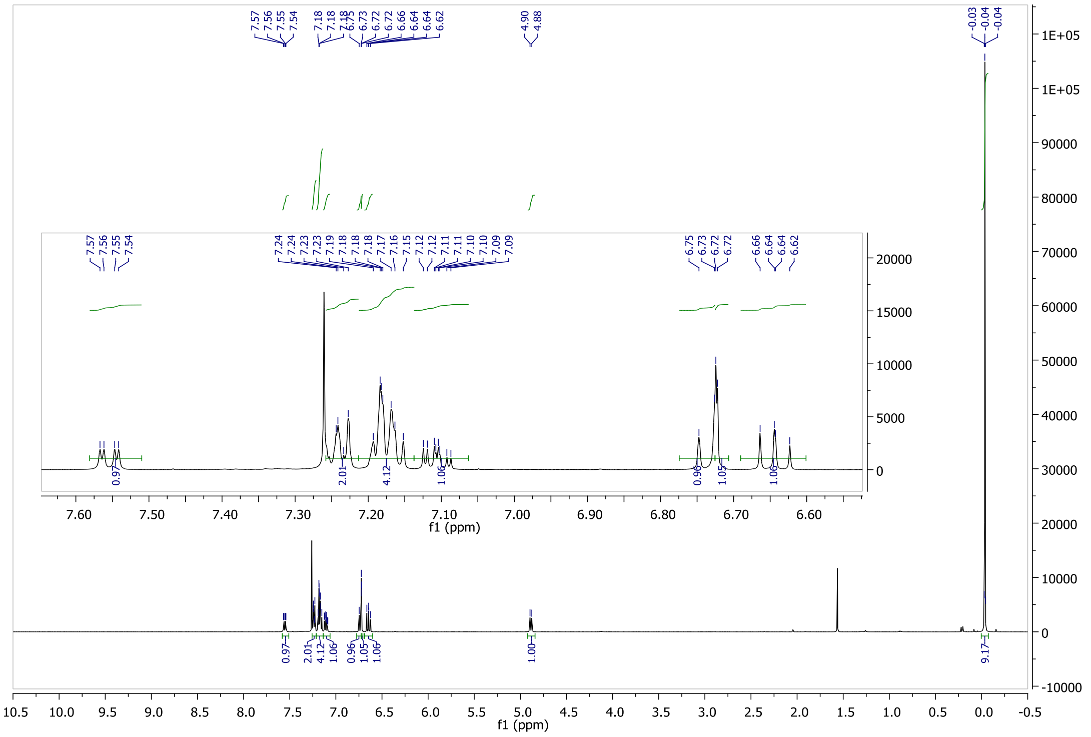
7.57
7.56
7.55
7.54
7.18
7.18
7.18
6.73
6.72
6.72
6.66
6.64
6.64
6.62
4.90
4.88
-0.03
-0.04
-0.04
0.97
2.01
4.12
1.06
0.96
1.05
1.06
1.00
9.17
f1 (ppm)

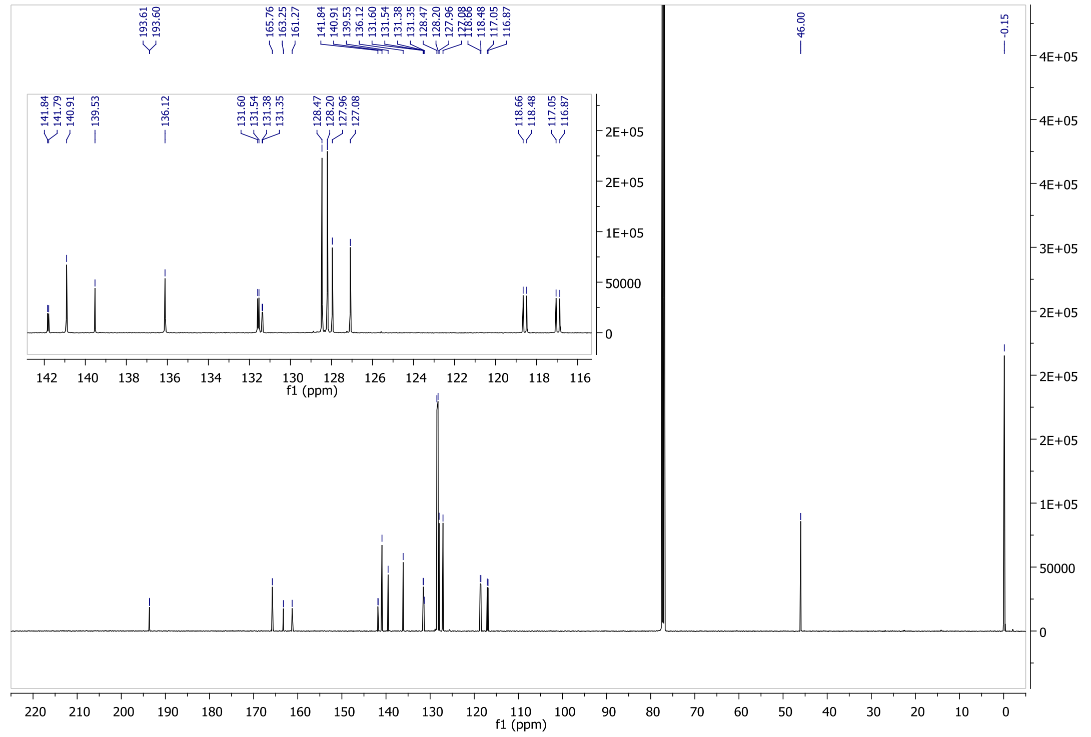
193.61
193.60
165.76
163.25
161.27
141.84
140.91
139.53
136.12
131.60
131.54
131.38
131.35
128.47
128.20
127.96
127.08
118.66
118.48
117.05
116.87
46.00
-0.15
f1 (ppm)

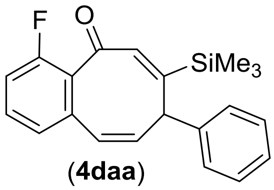

(**4daa**)

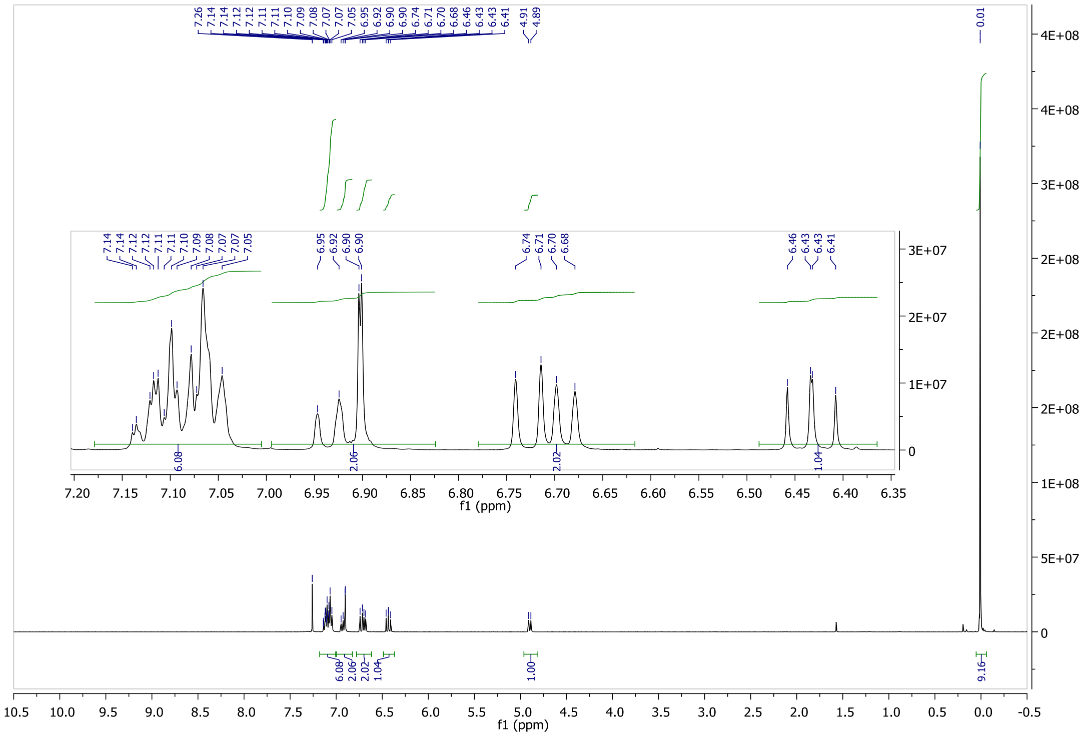

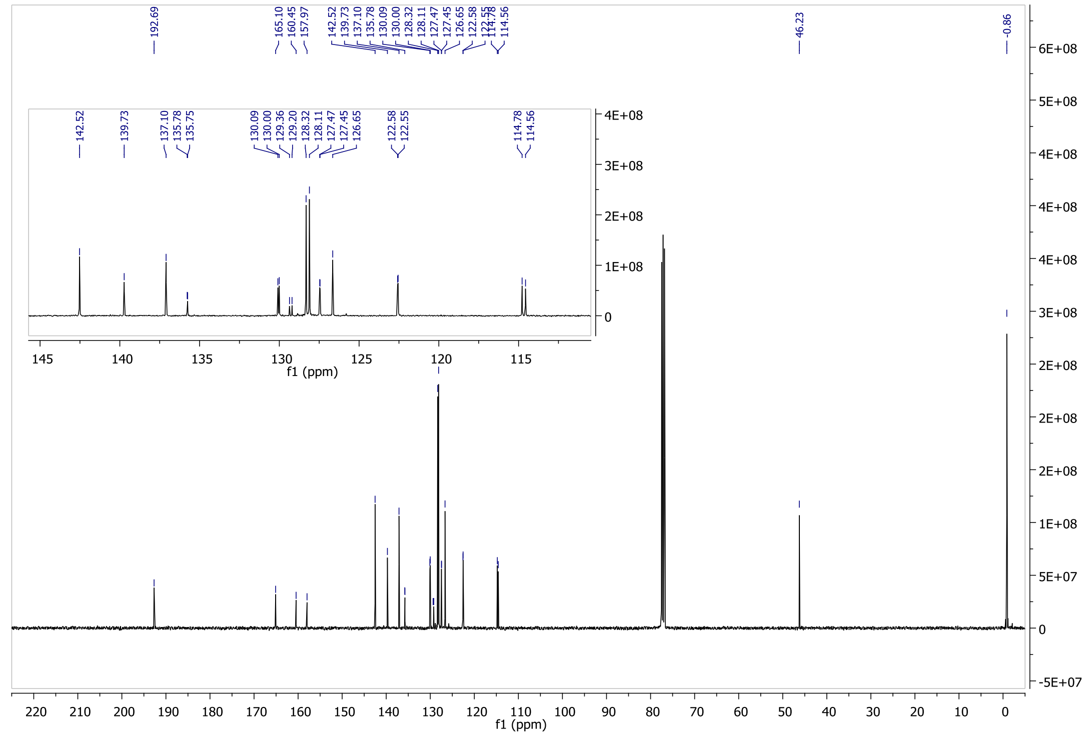

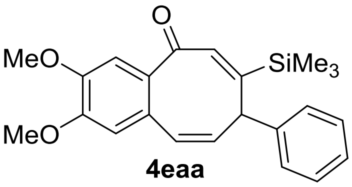

4eaa

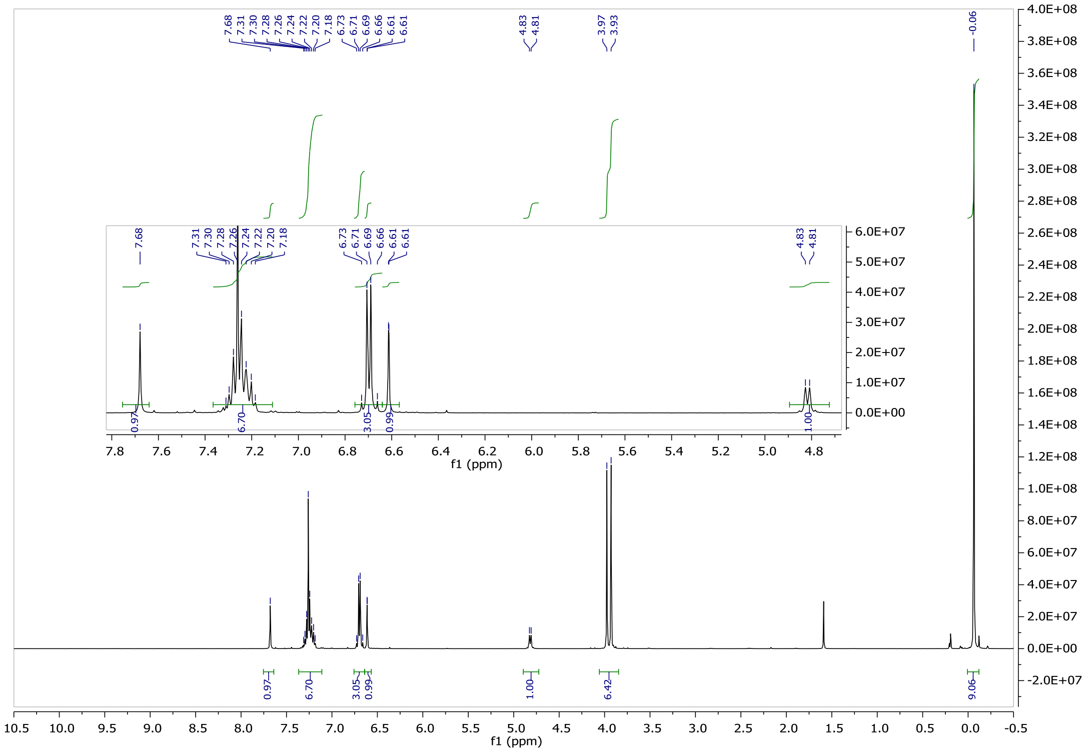

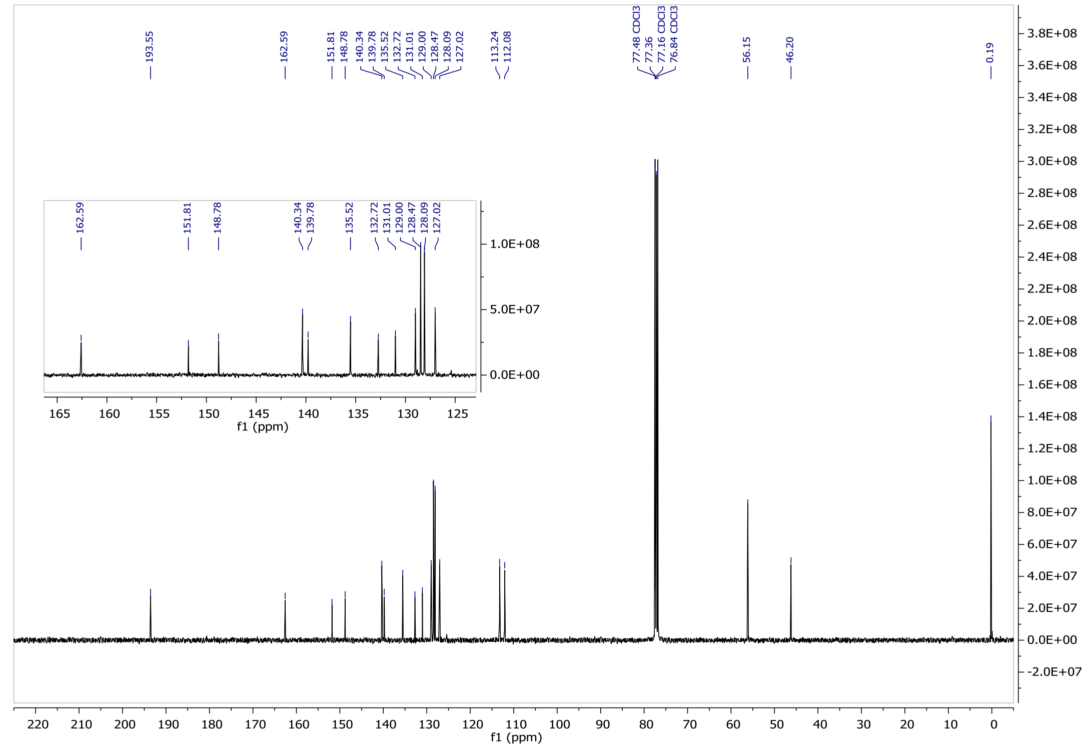

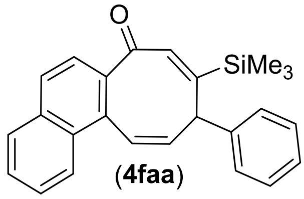
O
SiMe3
(4faa)

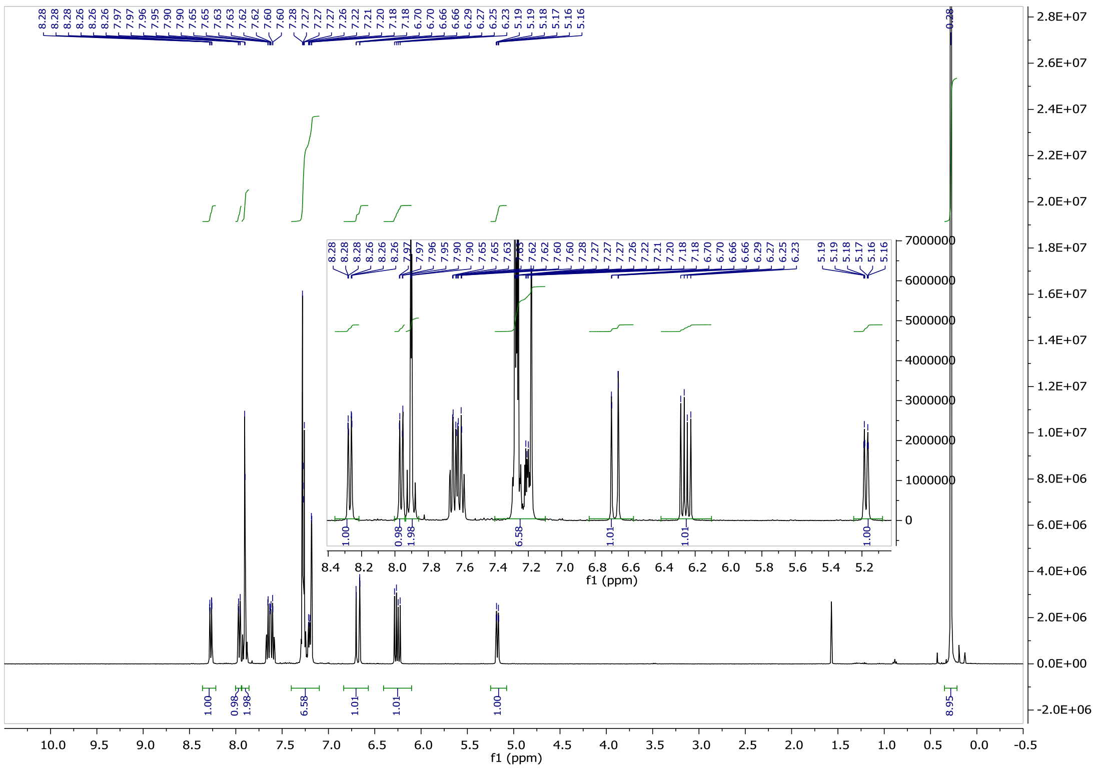

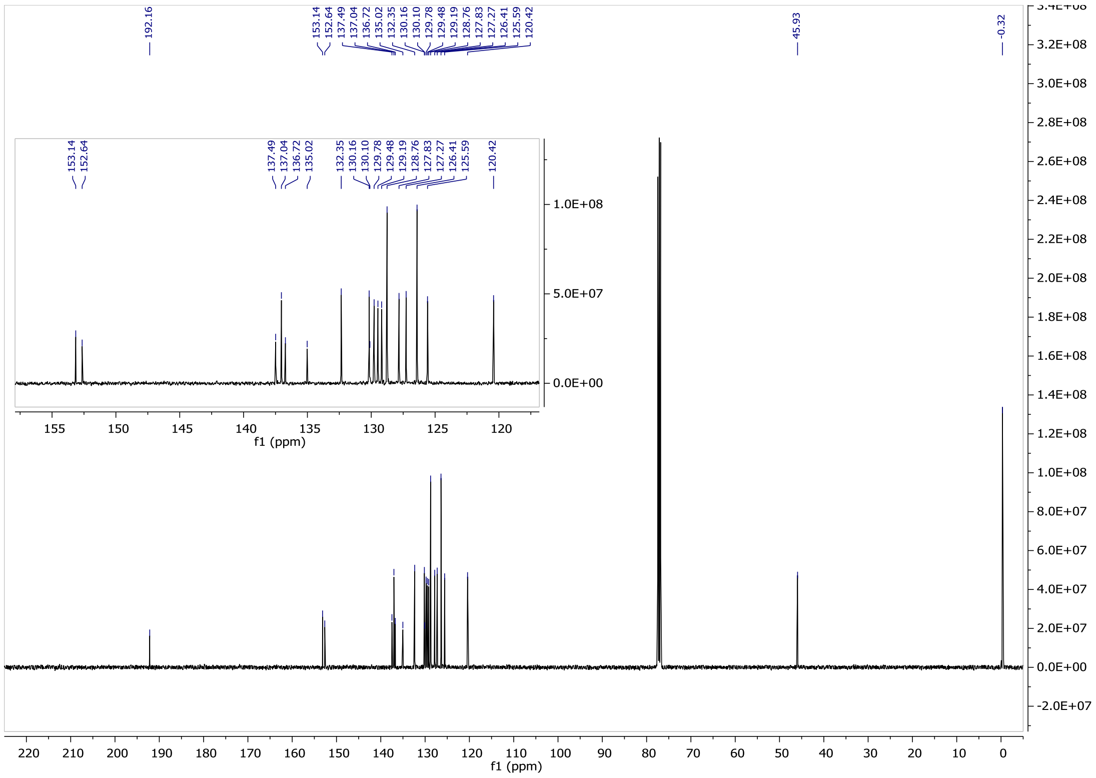

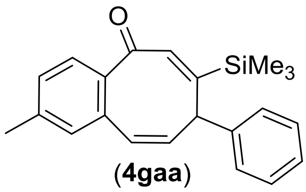
O
SiMe3

(**4gaa**)

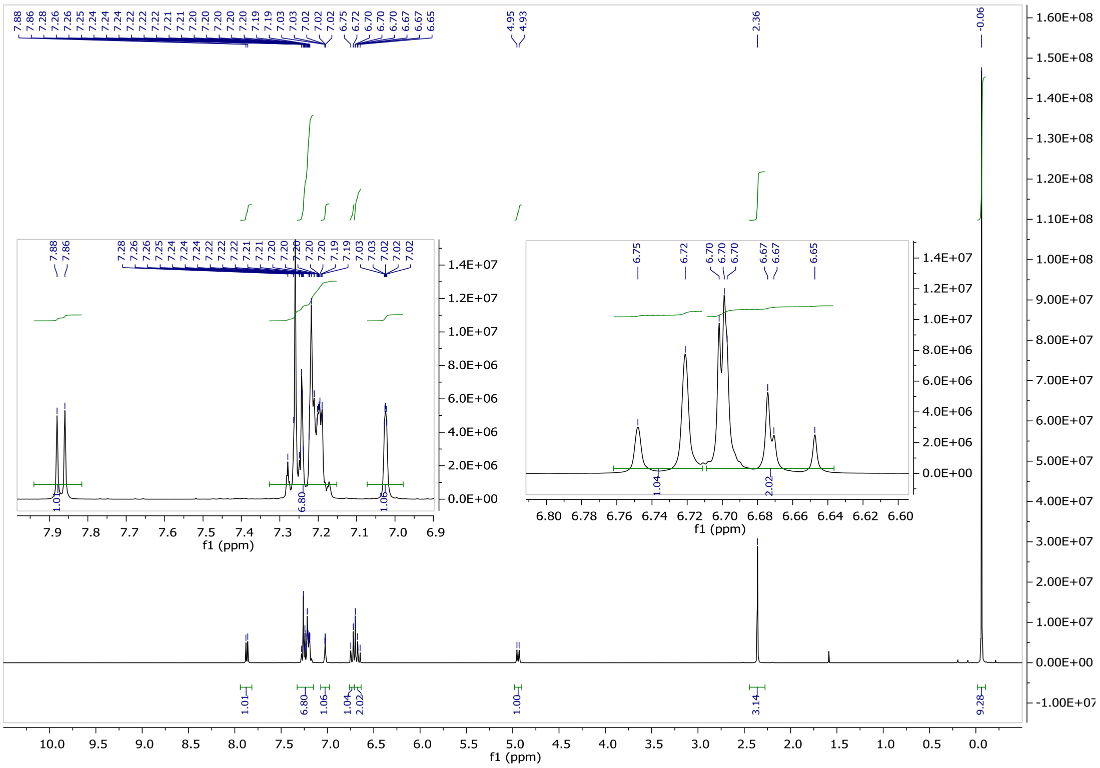
7.88 7.86 7.28 7.26 7.26 7.25 7.24 7.24 7.24 7.22 7.22 7.22 7.21 7.21 7.20 7.20 7.20 7.20 7.20 7.19 7.19 7.03 7.03 7.02 7.02 7.02 6.75 6.72 6.70 6.70 6.70 6.67 6.67 6.65
4.95 4.93
2.36
-0.06
1.01 6.80 1.06 1.04 2.02 1.00 3.14 9.28
f1 (ppm)

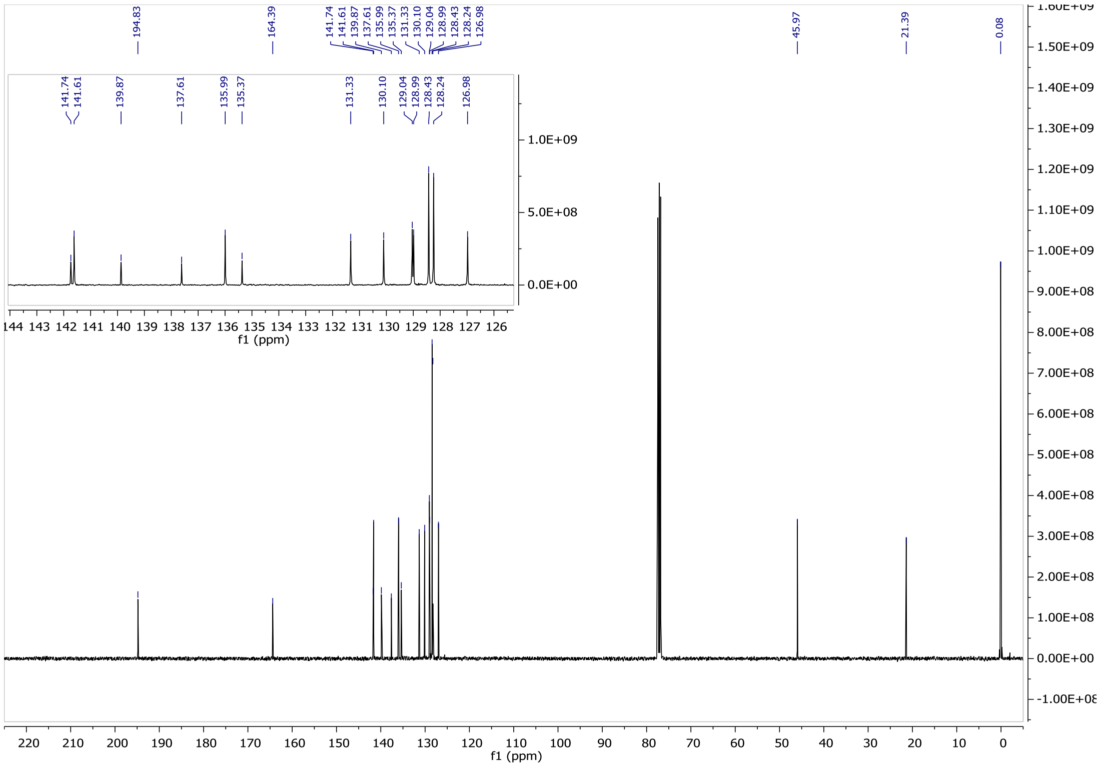
194.83
164.39
141.74 141.61 139.87 137.61 135.99 135.37 131.33 130.10 129.04 128.99 128.43 128.24 126.98
45.97
21.39
0.08
f1 (ppm)

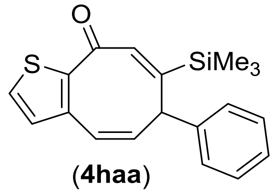
O
S
SiMe3

(**4haa**)

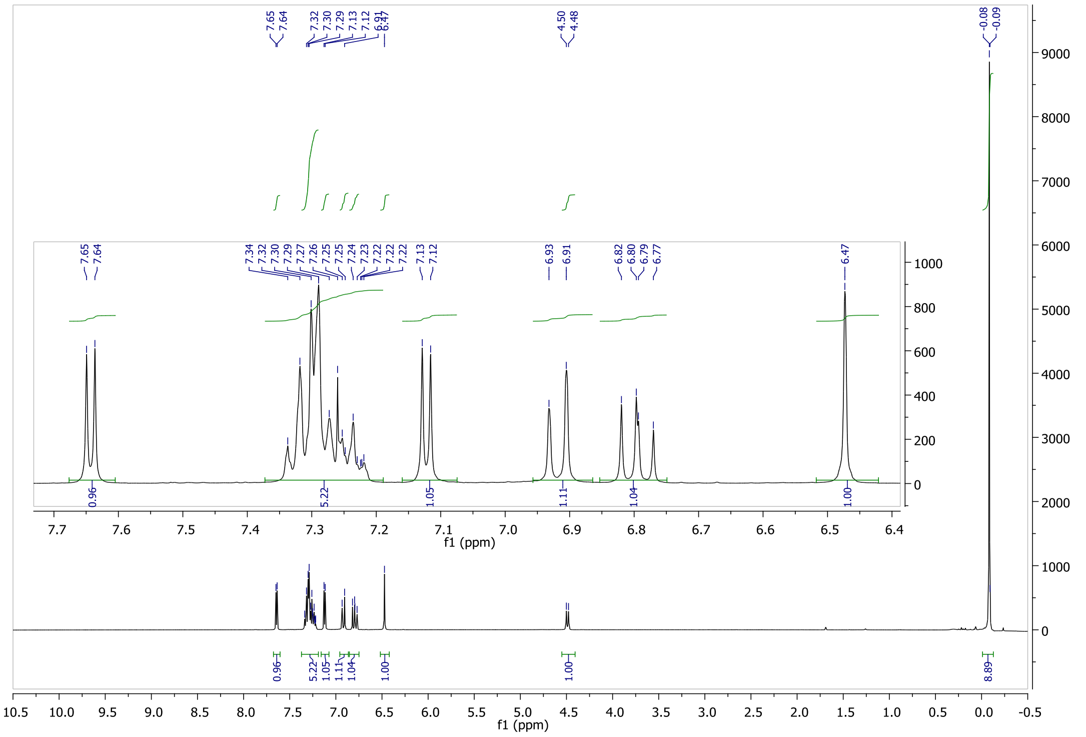
7.65
7.64
7.32
7.30
7.29
7.13
7.12
6.91
6.47
4.50
4.48
-0.08
-0.09
7.34
7.32
7.30
7.29
7.27
7.26
7.25
7.25
7.24
7.23
7.22
7.22
7.22
6.93
6.91
6.82
6.80
6.79
6.77
0.96
5.22
1.05
1.11
1.04
1.00
8.89
f1 (ppm)

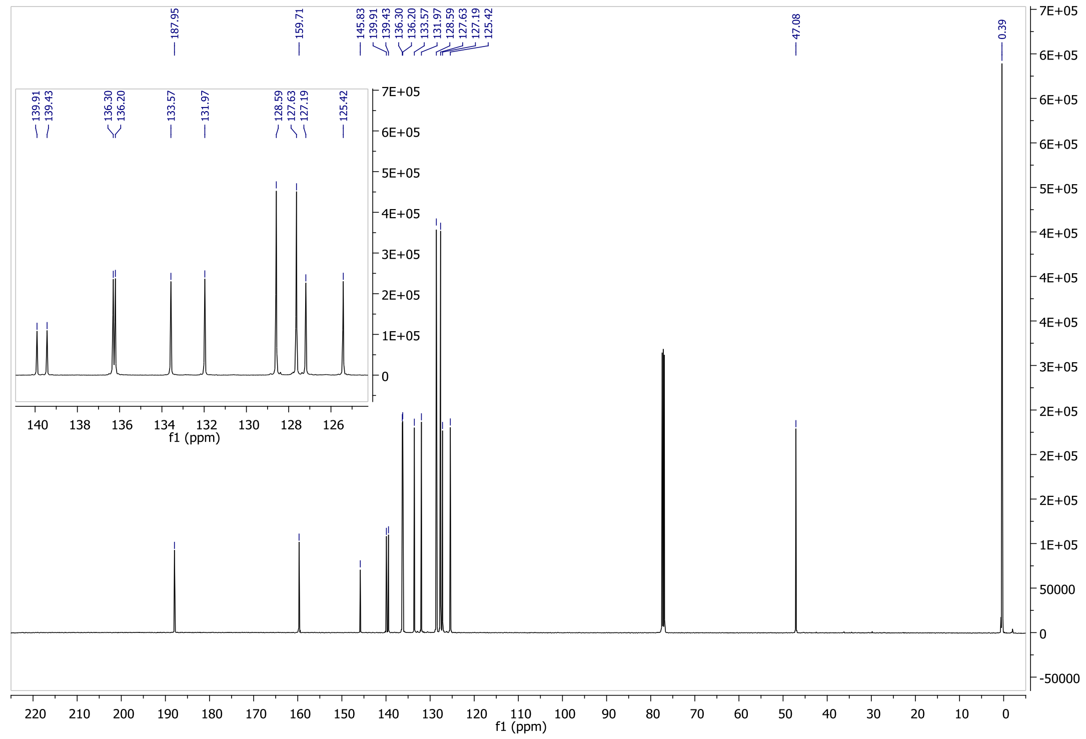
187.95
159.71
145.83
139.91
139.43
136.30
136.20
133.57
131.97
128.59
127.63
127.19
125.42
47.08
0.39
f1 (ppm)

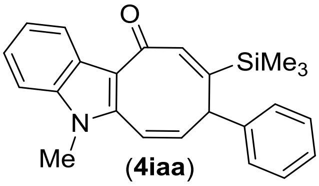
O
SiMe3
N
Me

(4iaa)

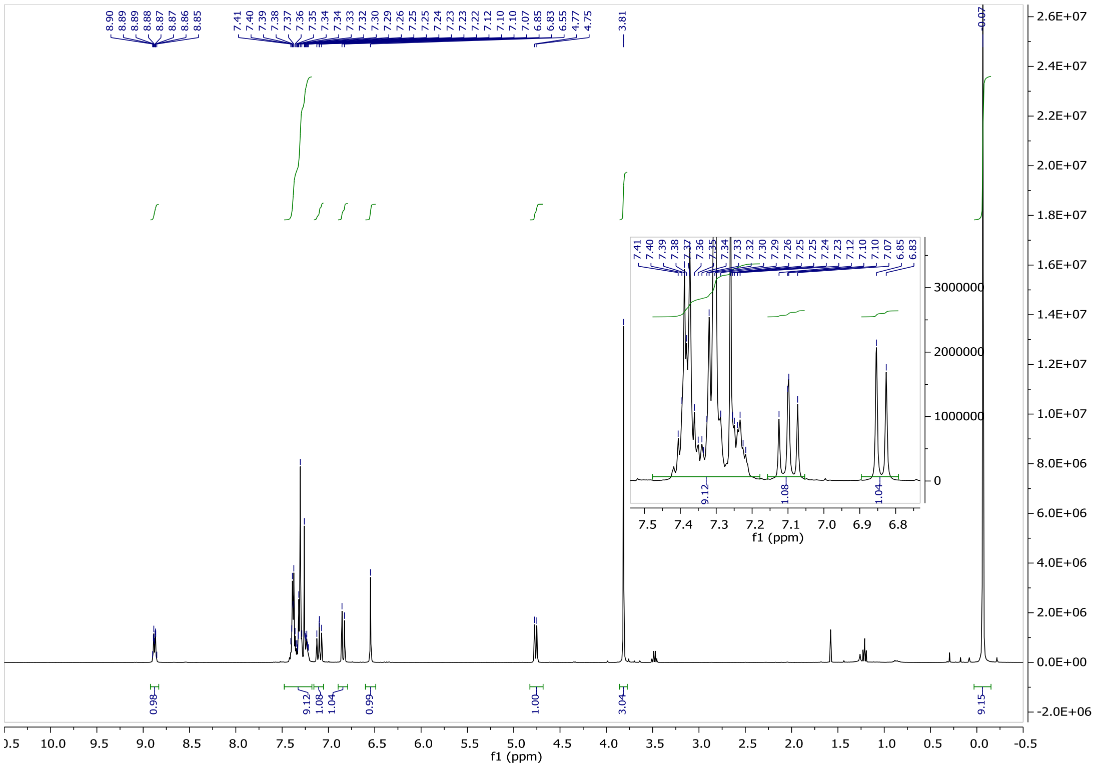
8.90 8.89 8.89 8.88 8.87 8.87 8.86 8.85
7.41 7.40 7.39 7.38 7.37 7.36 7.35 7.34 7.34 7.33 7.32 7.30 7.29 7.26 7.25 7.25 7.24 7.23 7.23 7.22 7.12 7.10 7.10 7.07 6.85 6.83 6.55 4.77 4.75
3.81
0.07
0.98 9.12 1.08 1.04 0.99 1.00 3.04 9.15
f1 (ppm)

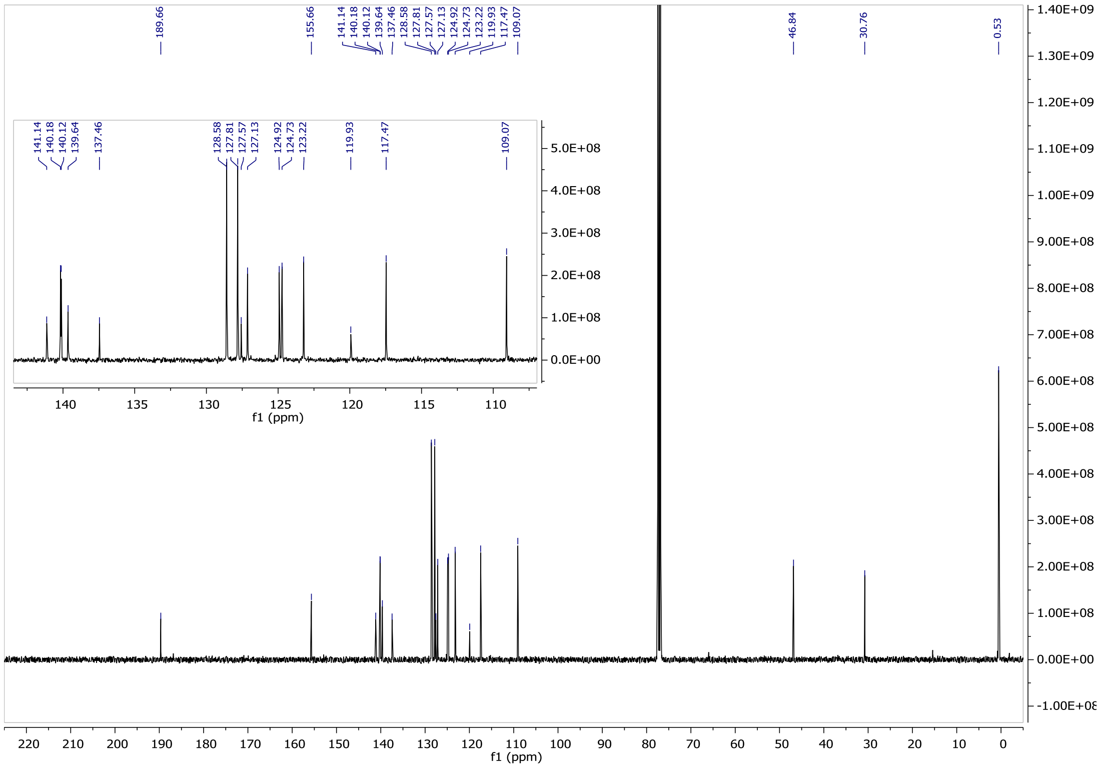
189.66
155.66
141.14 140.18 140.12 139.64 137.46 128.58 127.81 127.57 127.13 124.92 124.73 123.22 119.93 117.47 109.07
46.84
30.76
0.53
f1 (ppm)

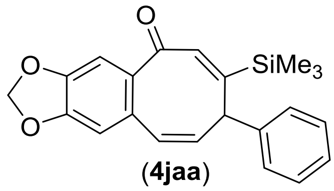

(**4jaa**)

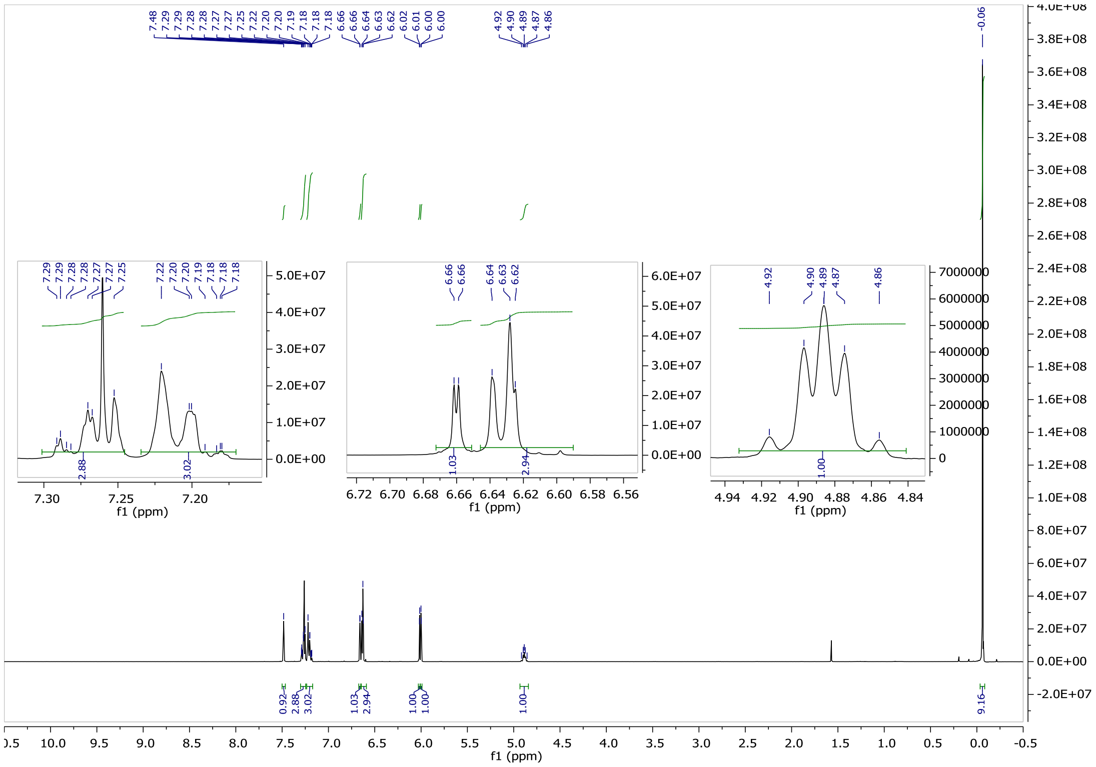

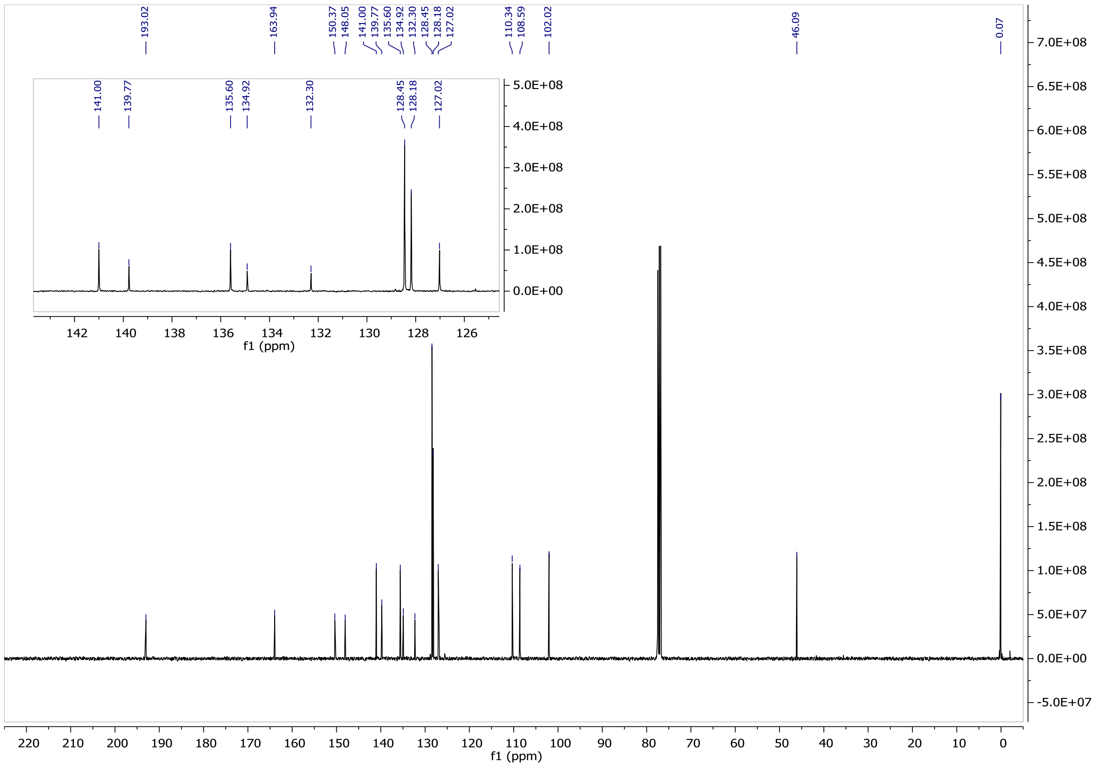

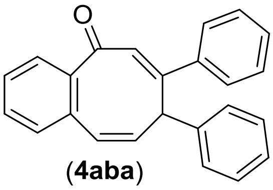

(**4aba**)

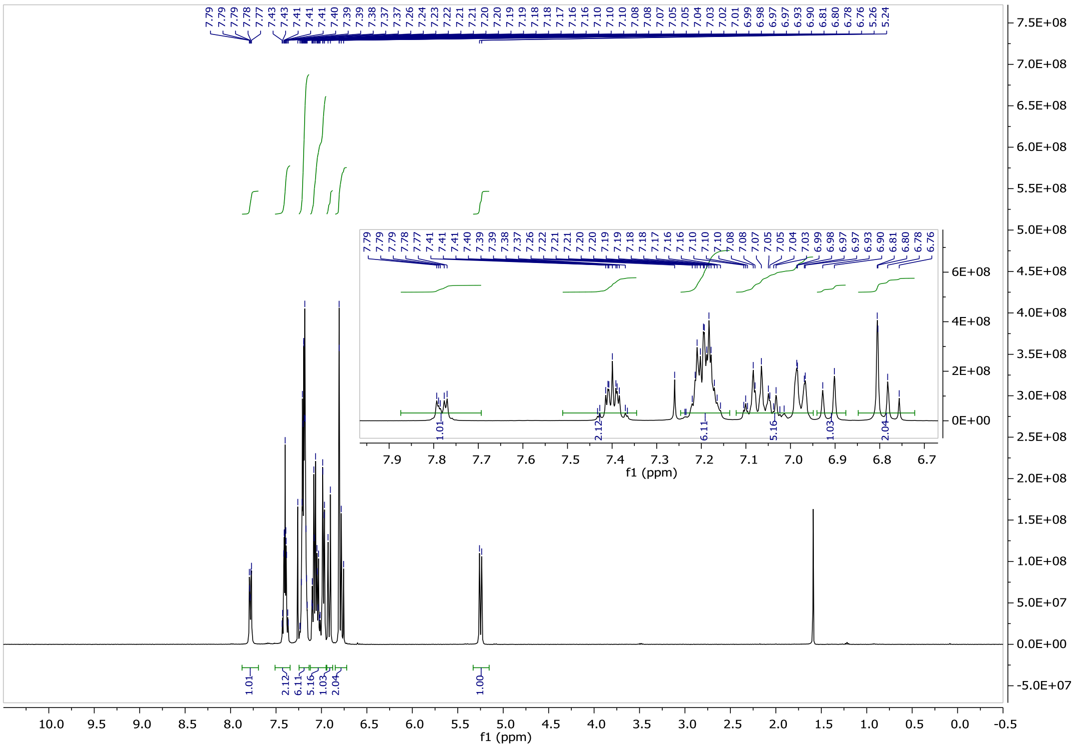
f1 (ppm)

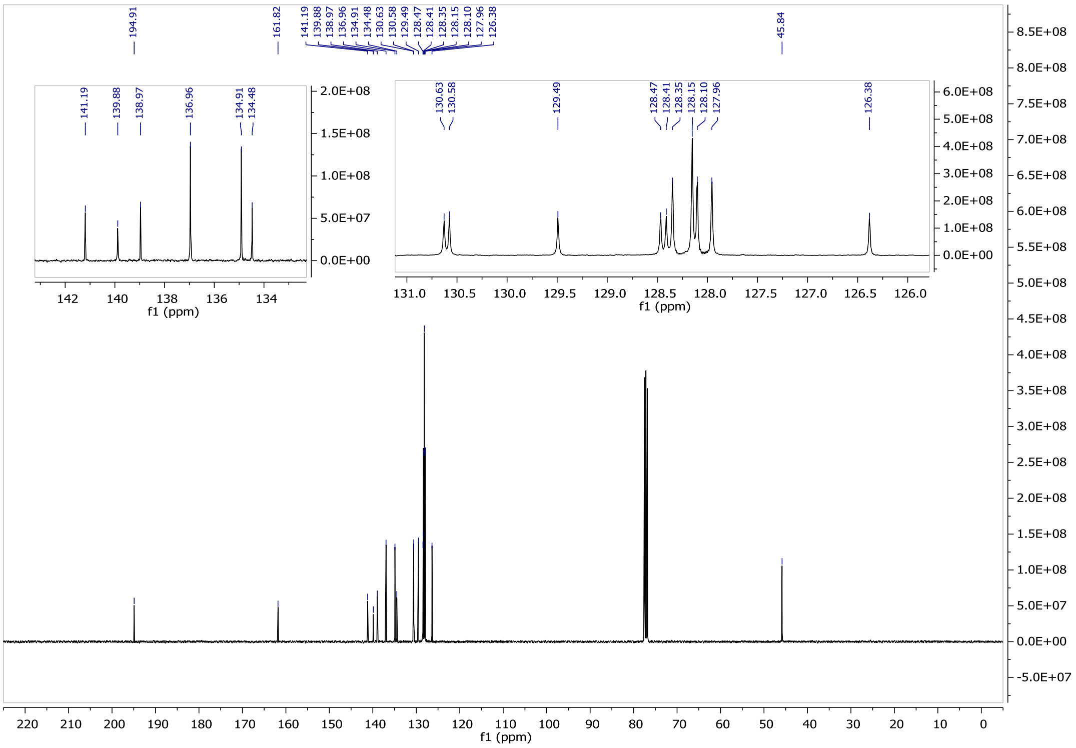
194.91
161.82
141.19
139.88
138.97
136.96
134.91
134.48
130.63
130.58
129.49
128.47
128.41
128.35
128.15
128.10
127.96
126.38
45.84
f1 (ppm)

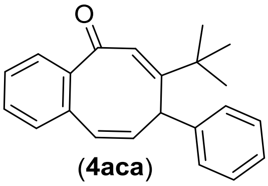

(**4aca**)

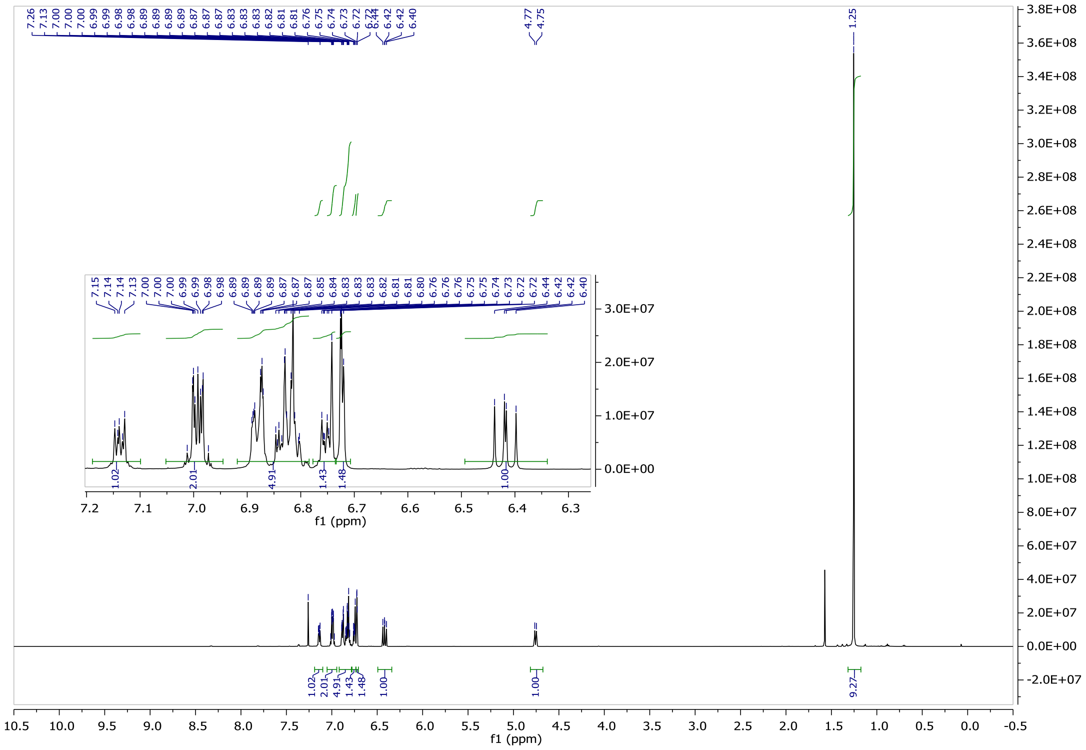

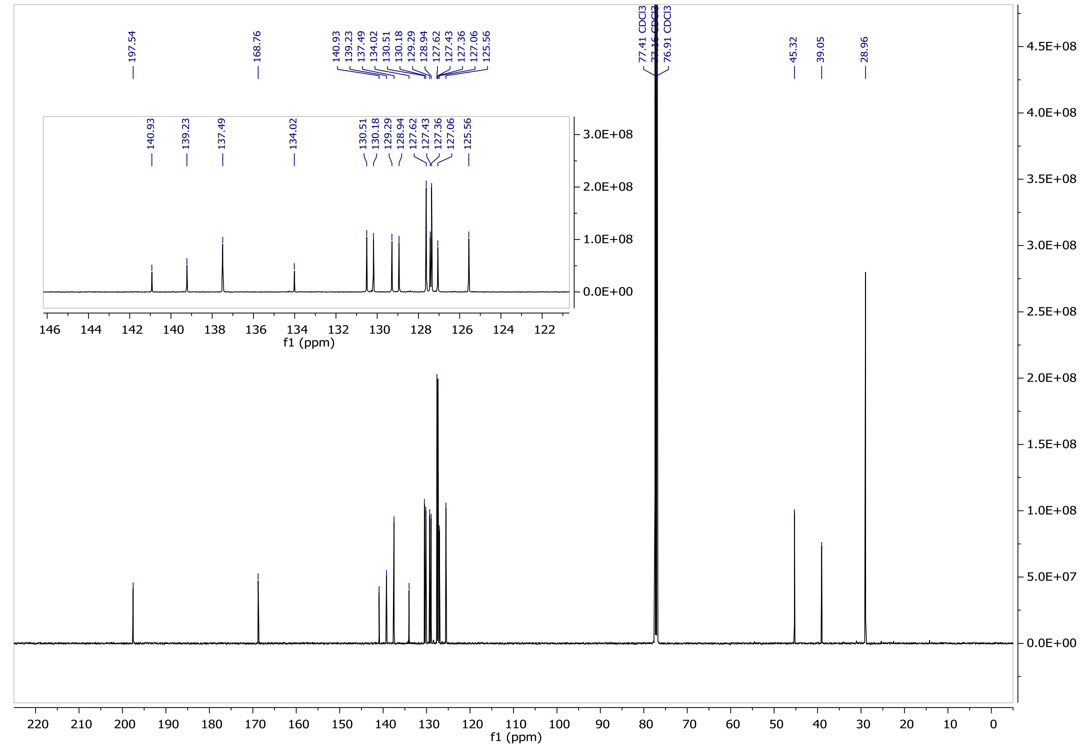

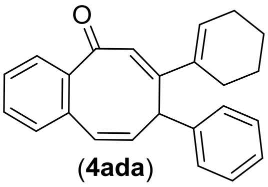

(**4ada**)

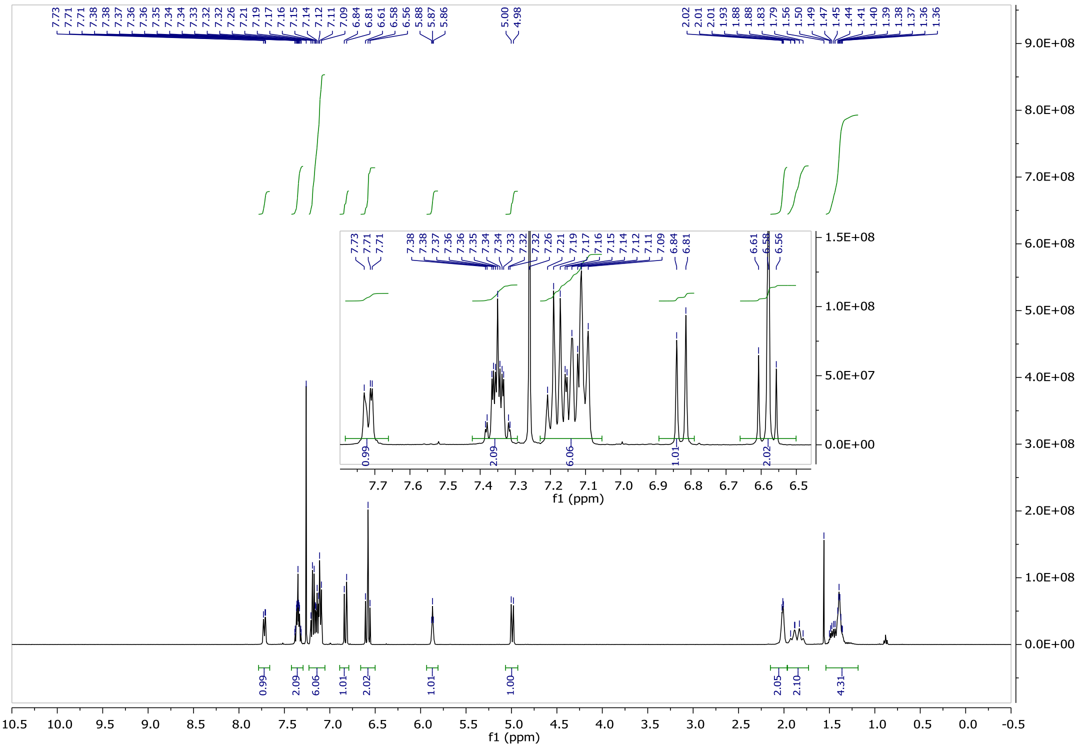
7.73 7.71 7.71 7.38 7.38 7.37 7.36 7.36 7.35 7.34 7.34 7.33 7.32 7.32 7.26 7.21 7.19 7.17 7.16 7.15 7.14 7.12 7.11 7.09 6.84 6.81 6.61 6.58 6.56 5.88 5.87 5.86
5.00 4.98
2.02 2.01 2.01 1.93 1.88 1.88 1.83 1.79 1.56 1.50 1.49 1.47 1.45 1.44 1.41 1.40 1.39 1.38 1.37 1.36 1.36
0.99 2.09 6.06 1.01 2.02 1.01 1.00 2.05 2.10 4.31
f1 (ppm)

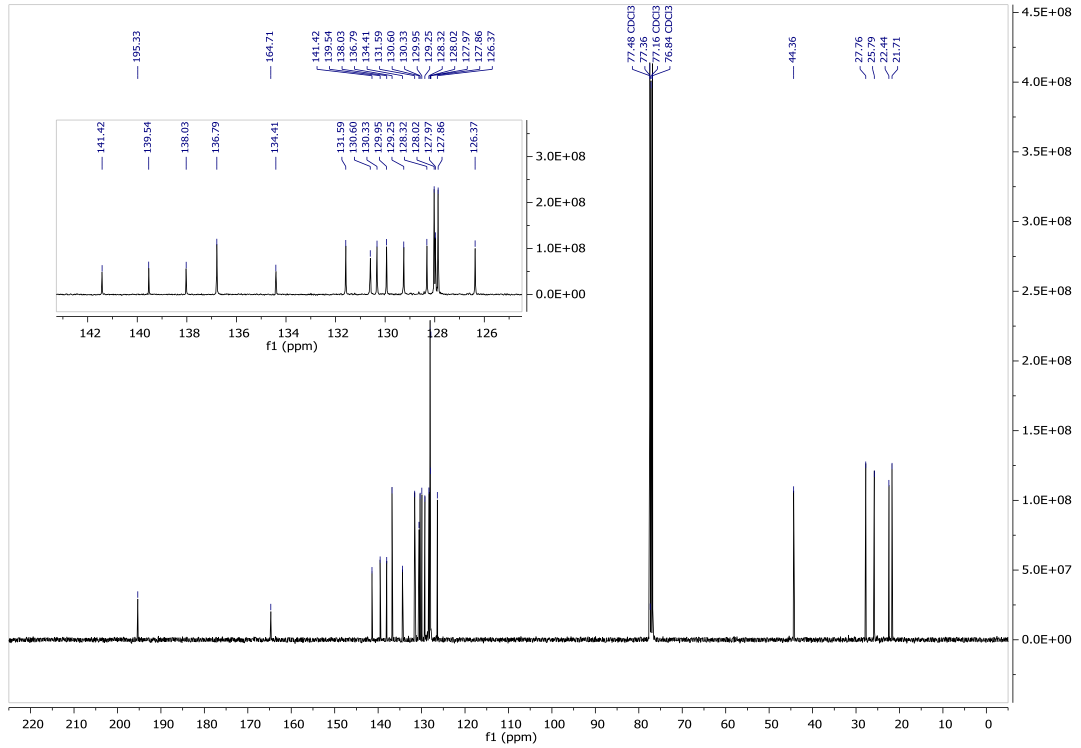
195.33
164.71
141.42 139.54 138.03 136.79 134.41 131.59 130.60 130.33 129.95 129.25 128.32 128.02 127.97 127.86 126.37
77.48 CDCl3 77.36 77.16 CDCl3 76.84 CDCl3
44.36
27.76 25.79 22.44 21.71
f1 (ppm)

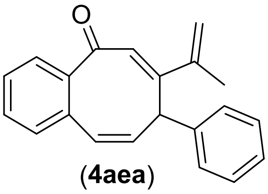

(**4aea**)

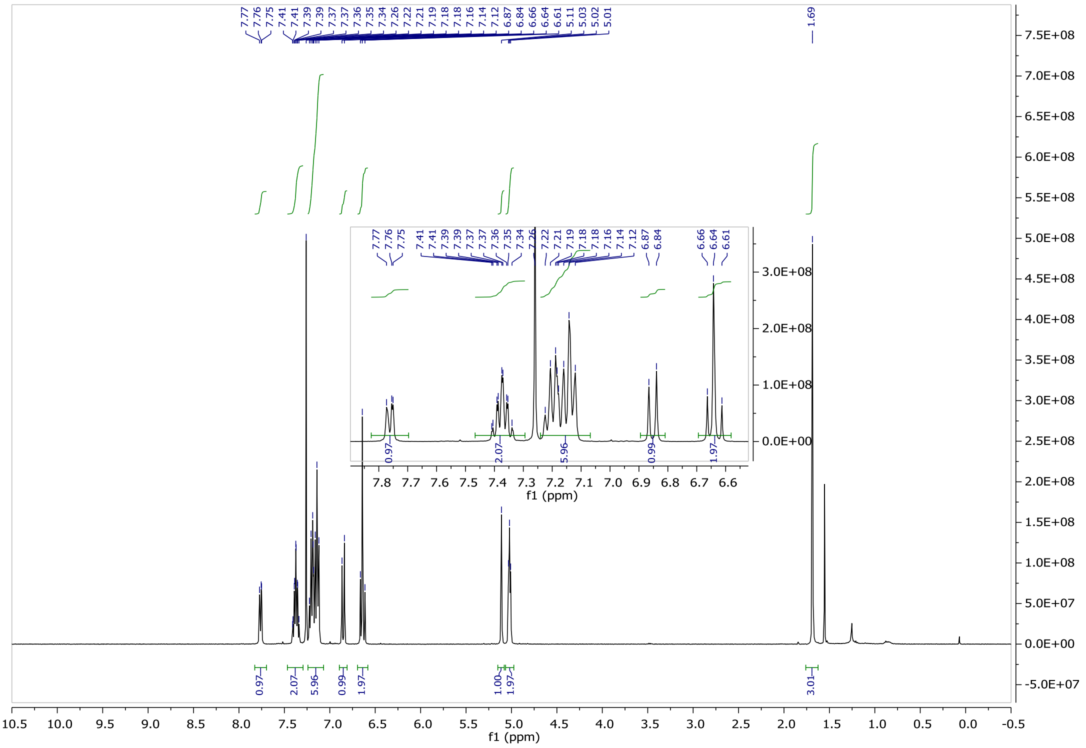
7.77 7.76 7.75 7.41 7.41 7.39 7.39 7.37 7.37 7.36 7.35 7.34 7.26 7.22 7.21 7.19 7.18 7.18 7.16 7.14 7.12 6.87 6.84 6.66 6.64 6.61 5.11 5.03 5.02 5.01
1.69
0.97 2.07 5.96 0.99 1.97 1.00 1.97 3.01
f1 (ppm)

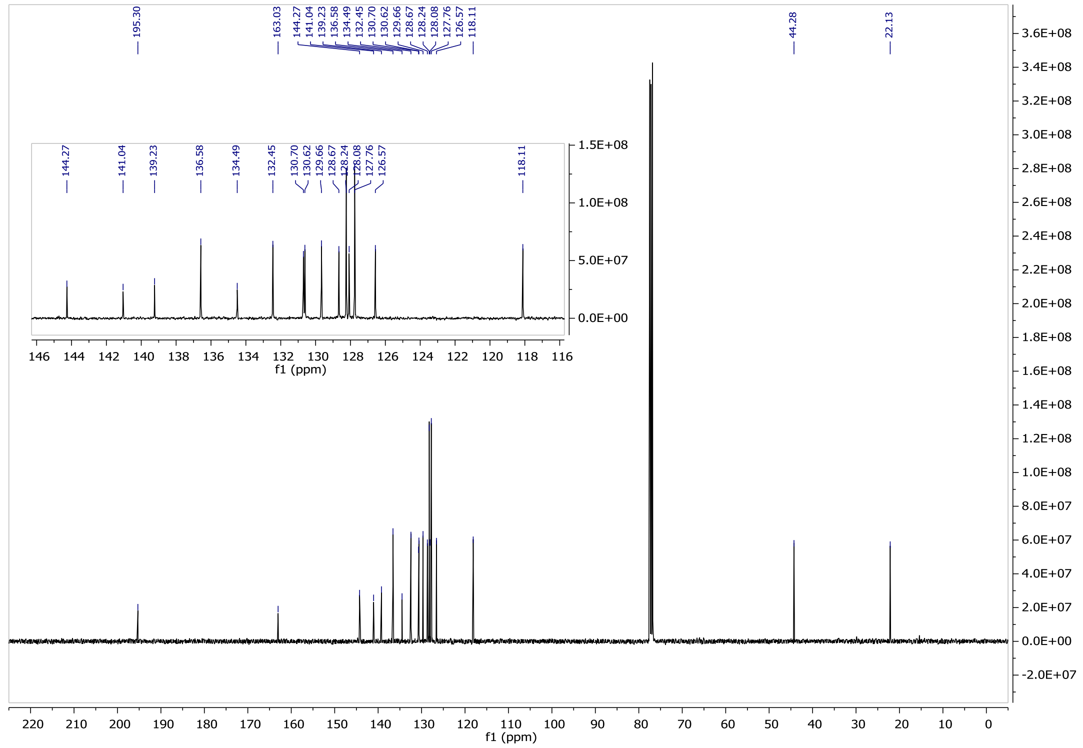
195.30
163.03
144.27 141.04 139.23 136.58 134.49 132.45 130.70 130.62 129.66 128.67 128.24 128.08 127.76 126.57 118.11
44.28
22.13
f1 (ppm)

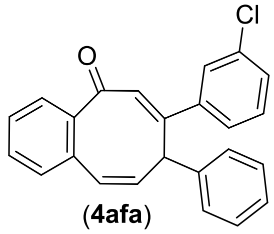

O
Cl

(**4afa**)

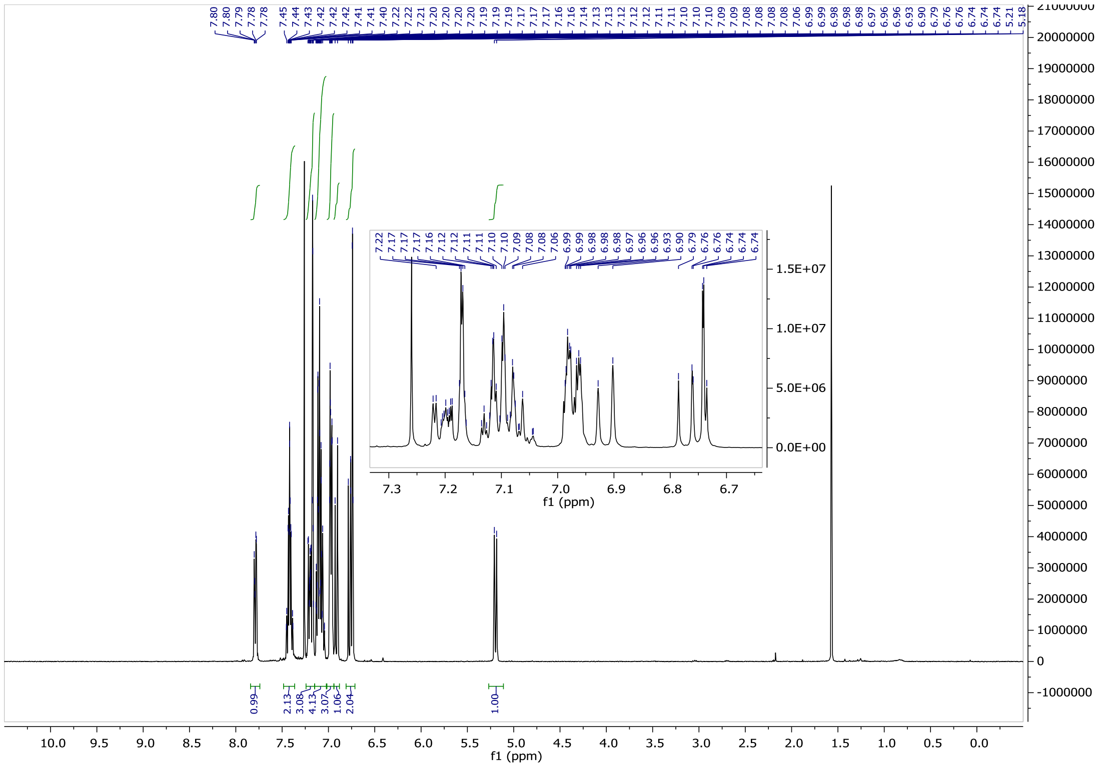

7.80
7.80
7.79
7.78
7.78
7.45
7.44
7.43
7.42
7.42
7.42
7.41
7.40
7.22
7.22
7.21
7.20
7.20
7.20
7.20
7.19
7.19
7.17
7.17
7.16
7.14
7.13
7.13
7.12
7.12
7.12
7.11
7.11
7.10
7.10
7.09
7.09
7.08
7.08
7.08
7.06
6.99
6.99
6.98
6.98
6.98
6.97
6.96
6.96
6.93
6.90
6.79
6.76
6.76
6.74
6.74
6.74
5.21
5.18
0.99
2.13
3.08
4.13
3.07
1.06
2.04
1.00
f1 (ppm)

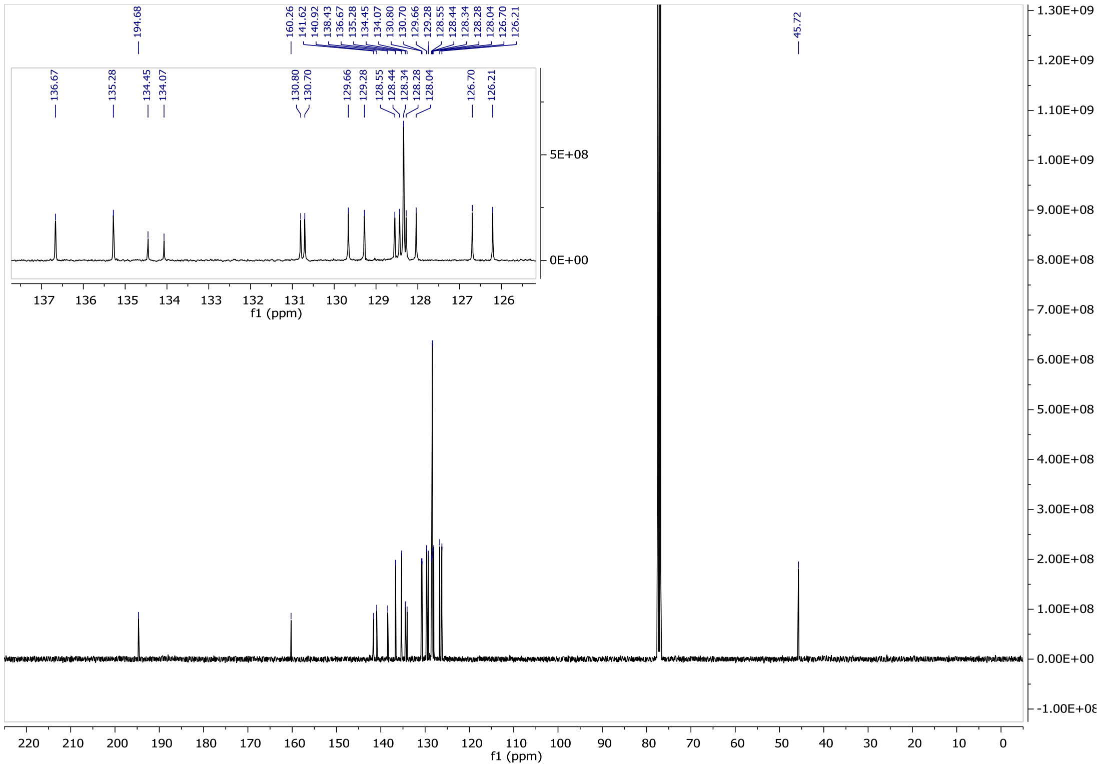

194.68
160.26
141.62
140.92
138.43
136.67
135.28
134.45
134.07
130.80
130.70
129.66
129.28
128.55
128.44
128.34
128.28
128.04
126.70
126.21
45.72
f1 (ppm)

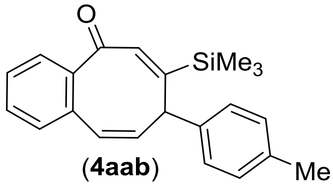
SiMe3
Me

(**4aab**)

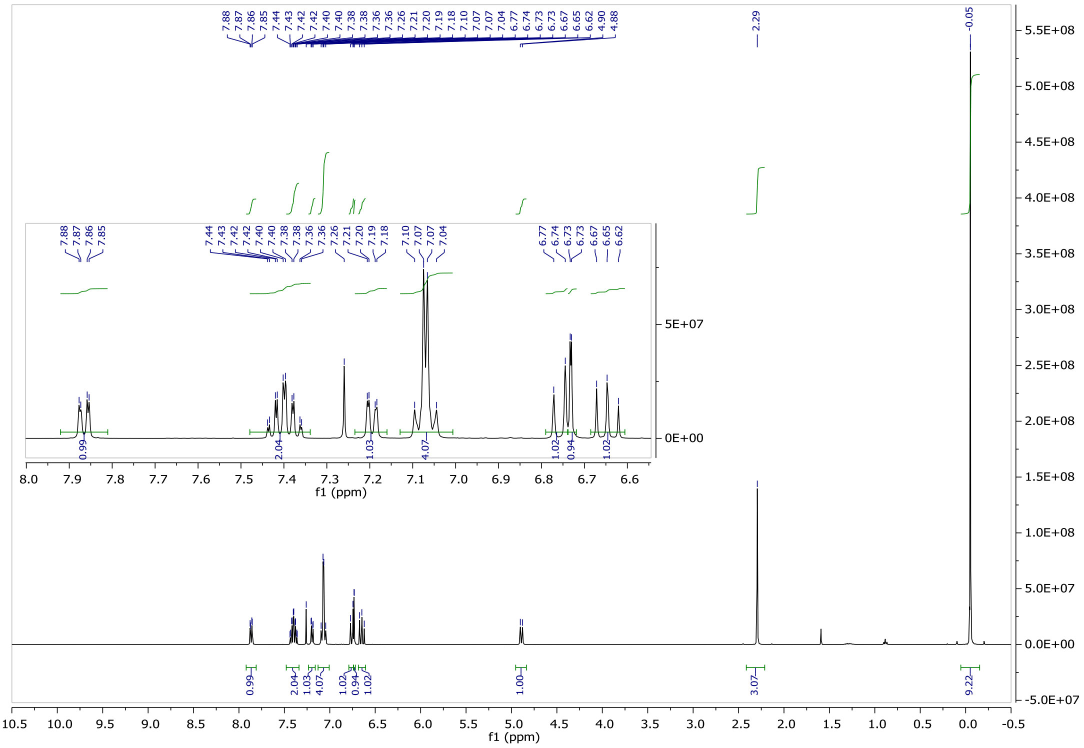
7.88 7.87 7.86 7.85 7.44 7.43 7.42 7.42 7.40 7.40 7.38 7.38 7.36 7.36 7.26 7.21 7.20 7.19 7.18 7.10 7.07 7.07 7.04 6.77 6.74 6.73 6.73 6.67 6.65 6.62 4.90 4.88
2.29
-0.05
0.99 2.04 1.03 4.07 1.02 0.94 1.02 1.00 3.07 9.22
f1 (ppm)

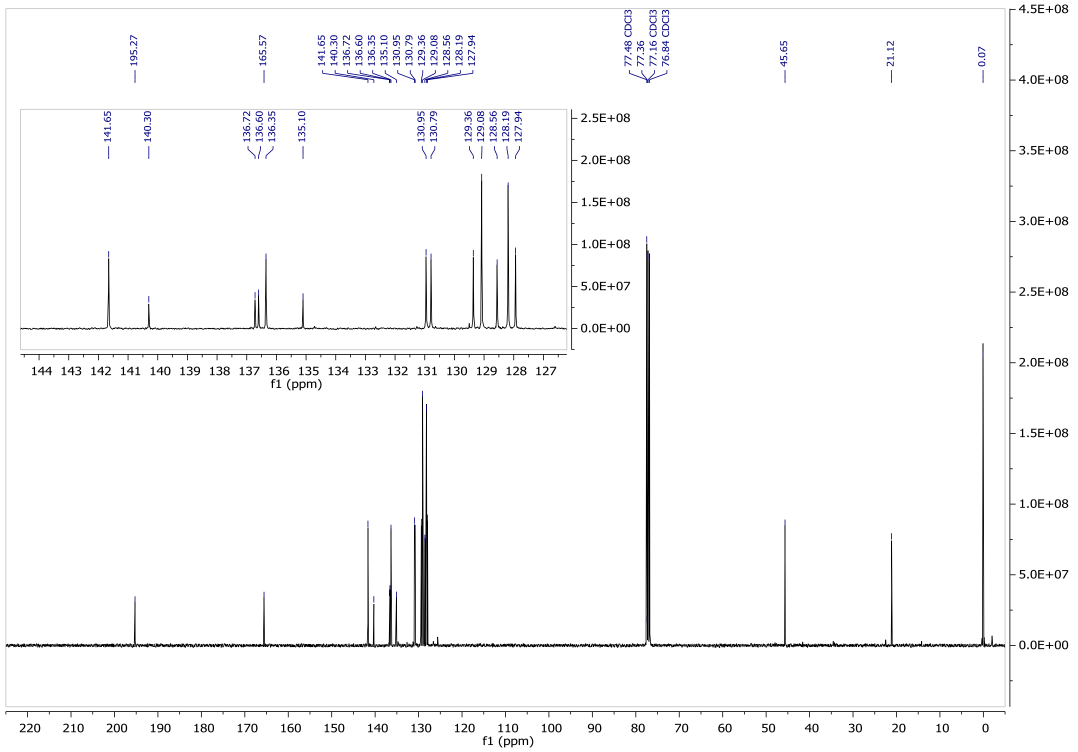
195.27
165.57
141.65 140.30 136.72 136.60 136.35 135.10 130.95 130.79 129.36 129.08 128.56 128.19 127.94
77.48 CDCl3 77.36 77.16 CDCl3 76.84 CDCl3
45.65
21.12
0.07
f1 (ppm)

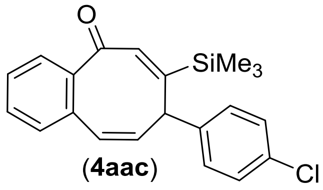
O
SiMe3
Cl

(**4aac**)

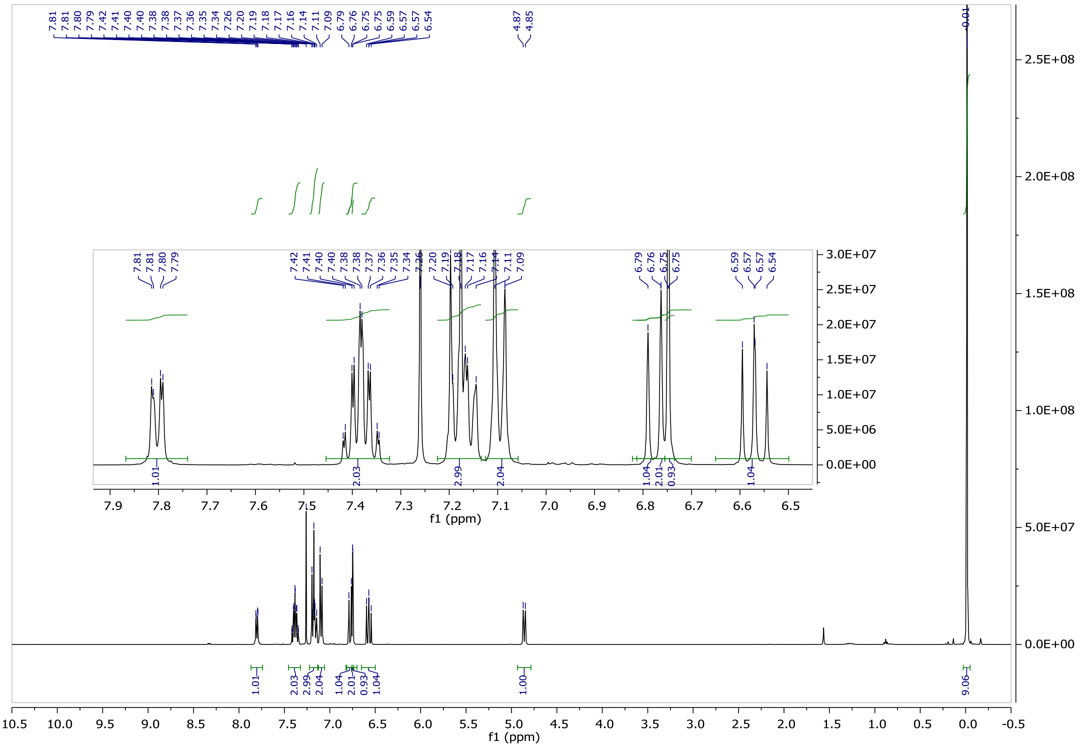
7.81 7.81 7.80 7.79 7.42 7.41 7.40 7.40 7.38 7.38 7.37 7.36 7.35 7.34 7.26 7.20 7.19 7.18 7.17 7.16 7.14 7.11 7.09 6.79 6.76 6.75 6.75 6.59 6.57 6.57 6.54
4.87 4.85
0.01
1.01 2.03 2.99 2.04 1.04 2.01 0.93 1.04 1.00 9.06
f1 (ppm)

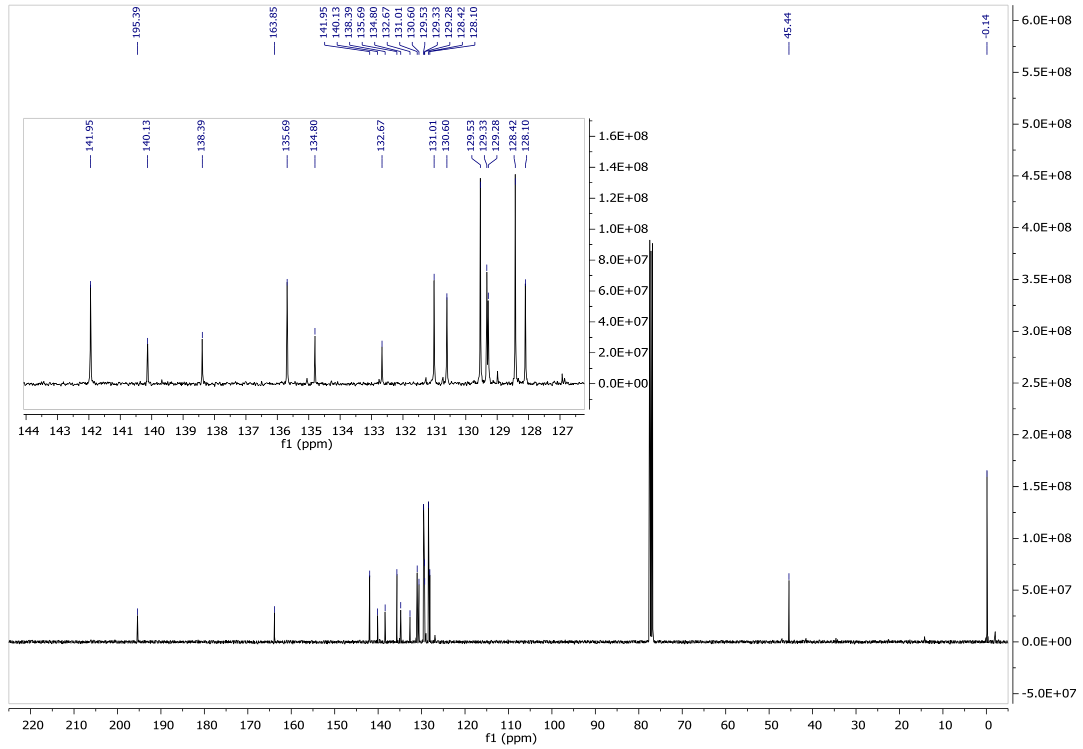
195.39
163.85
141.95 140.13 138.39 135.69 134.80 132.67 131.01 130.60 129.53 129.33 129.28 128.42 128.10
45.44
-0.14
f1 (ppm)

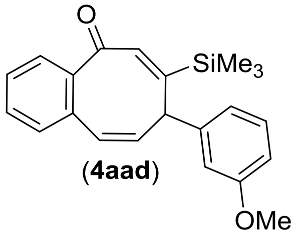

(**4aad**)

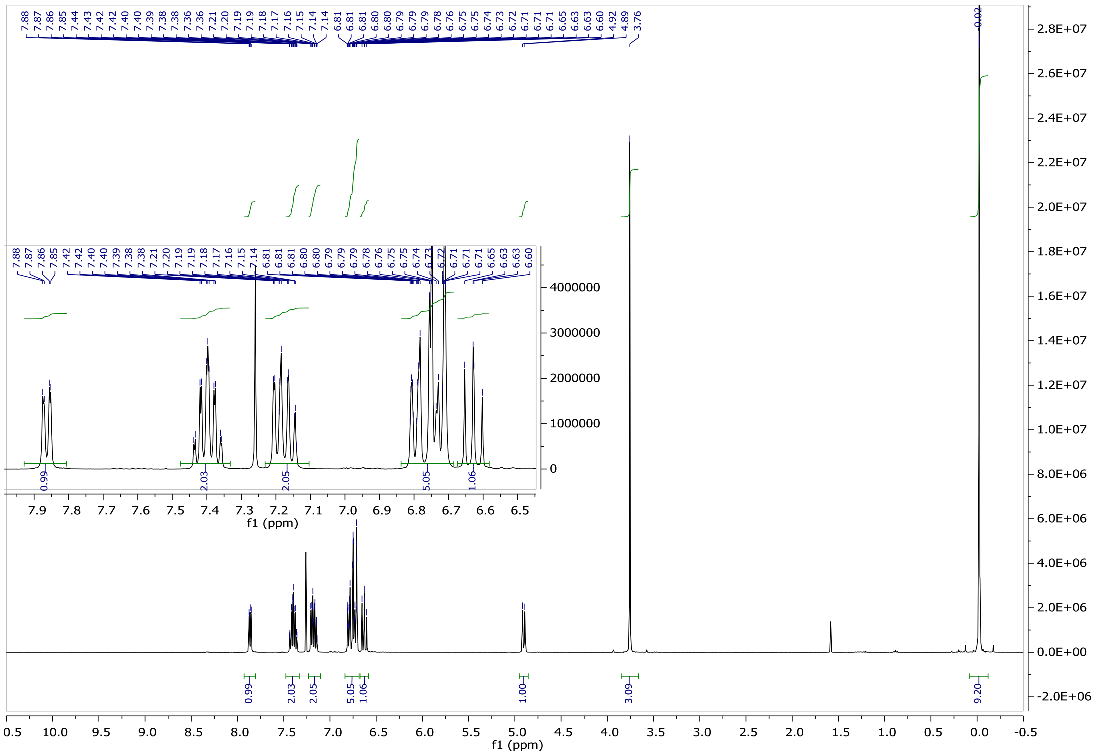

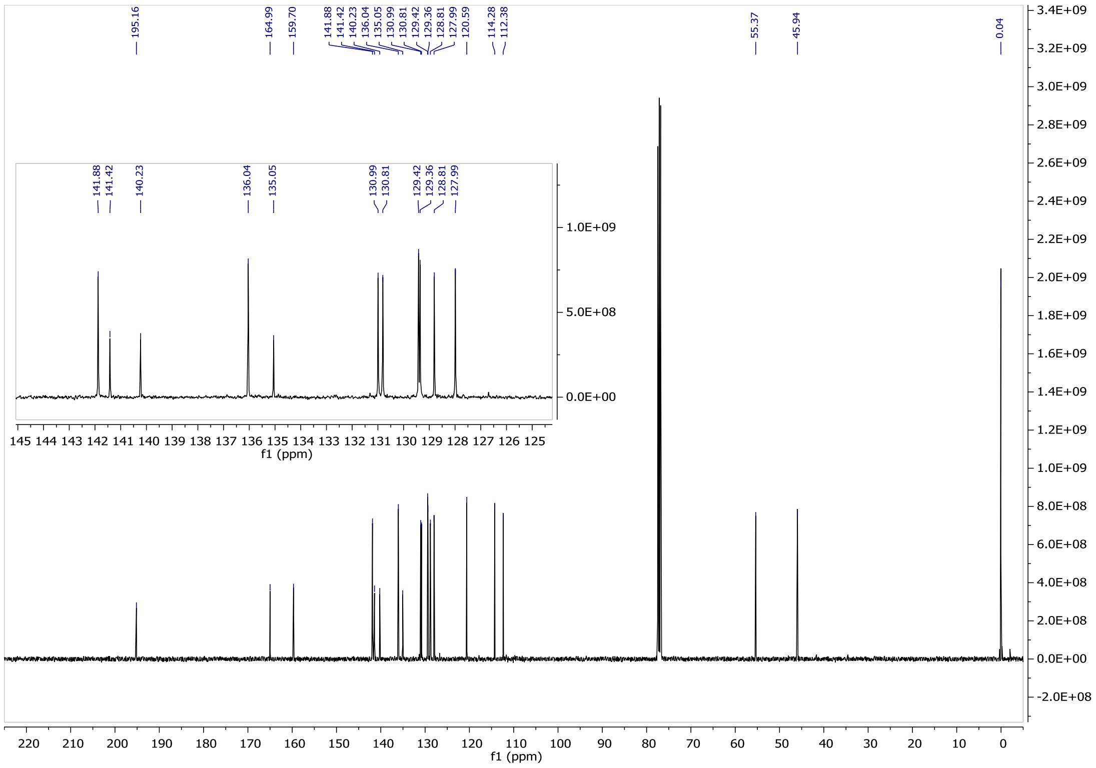

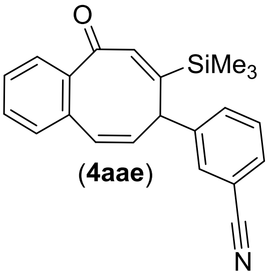
O
SiMe3
N

(**4aae**)

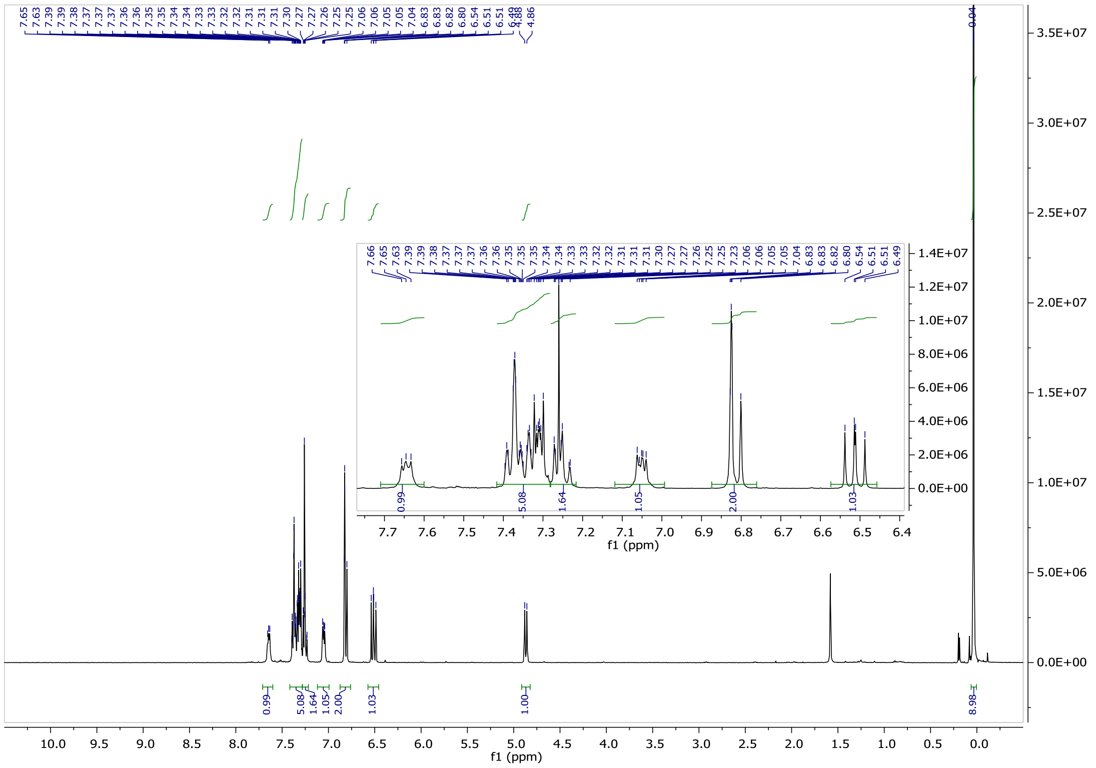
f1 (ppm)

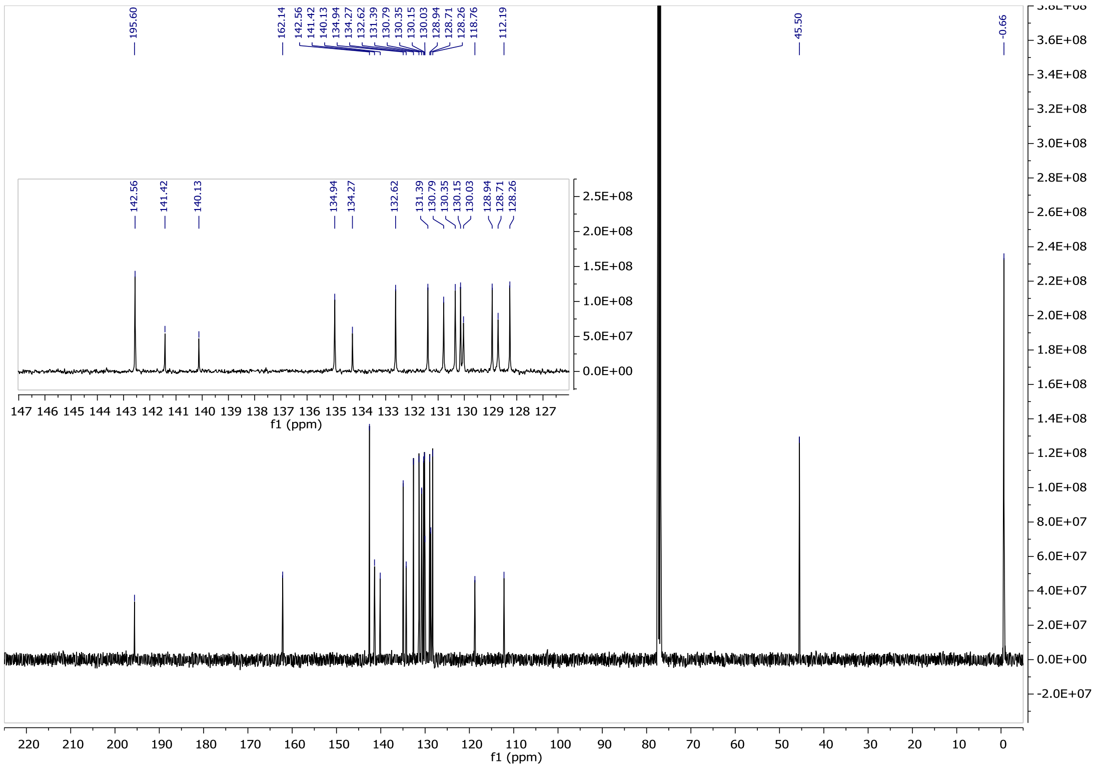
195.60
162.14
142.56
141.42
140.13
134.94
134.27
132.62
131.39
130.79
130.35
130.15
130.03
128.94
128.71
128.26
118.76
112.19
45.50
-0.66
f1 (ppm)

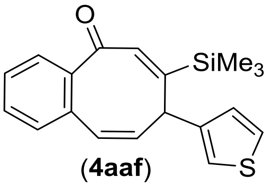

(**4aaf**)

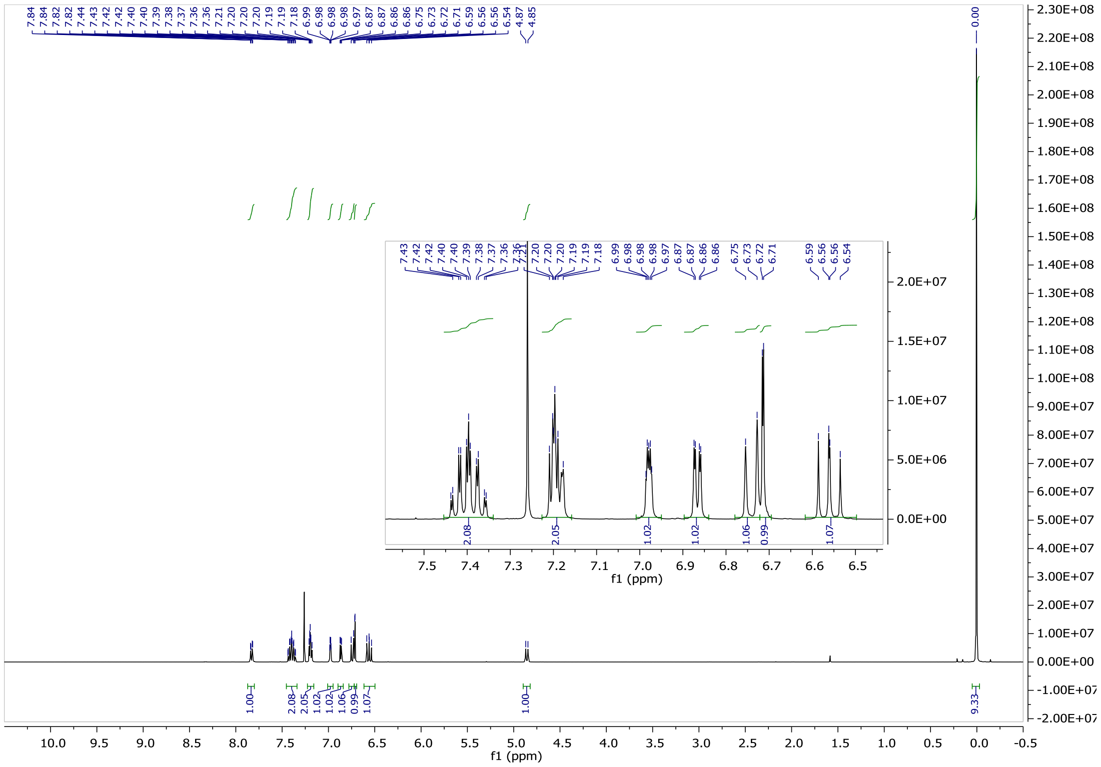

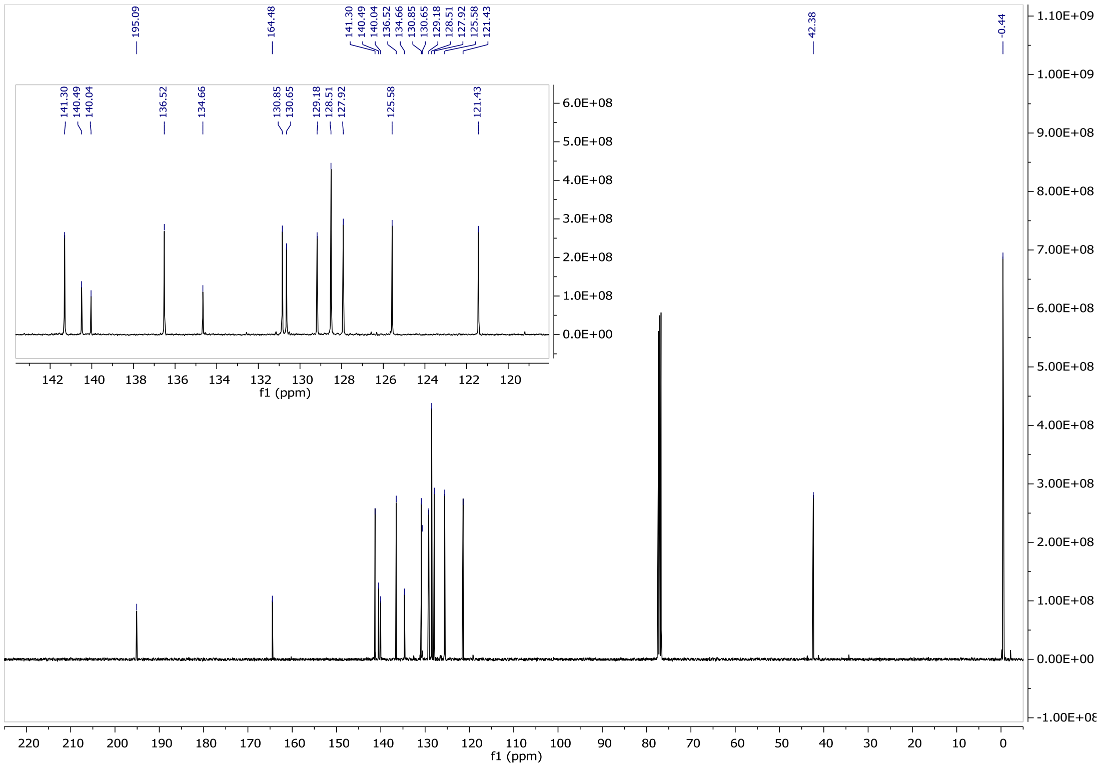

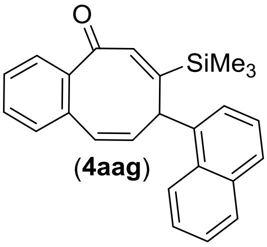

(**4aag**)

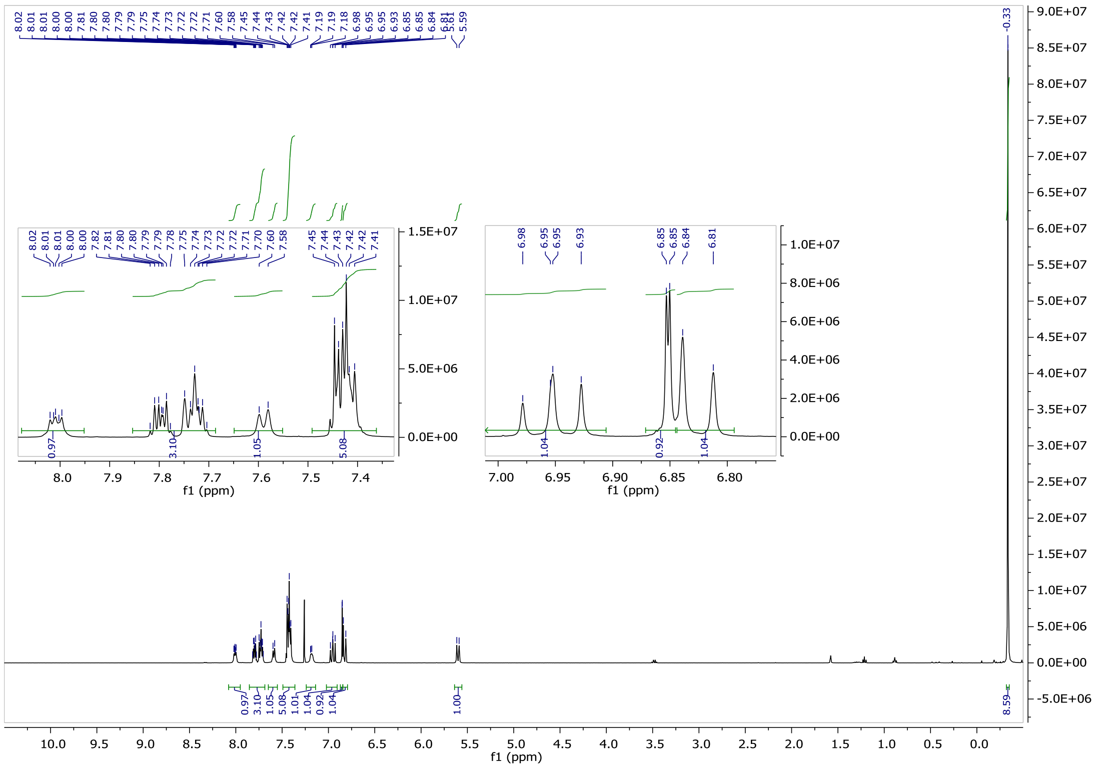

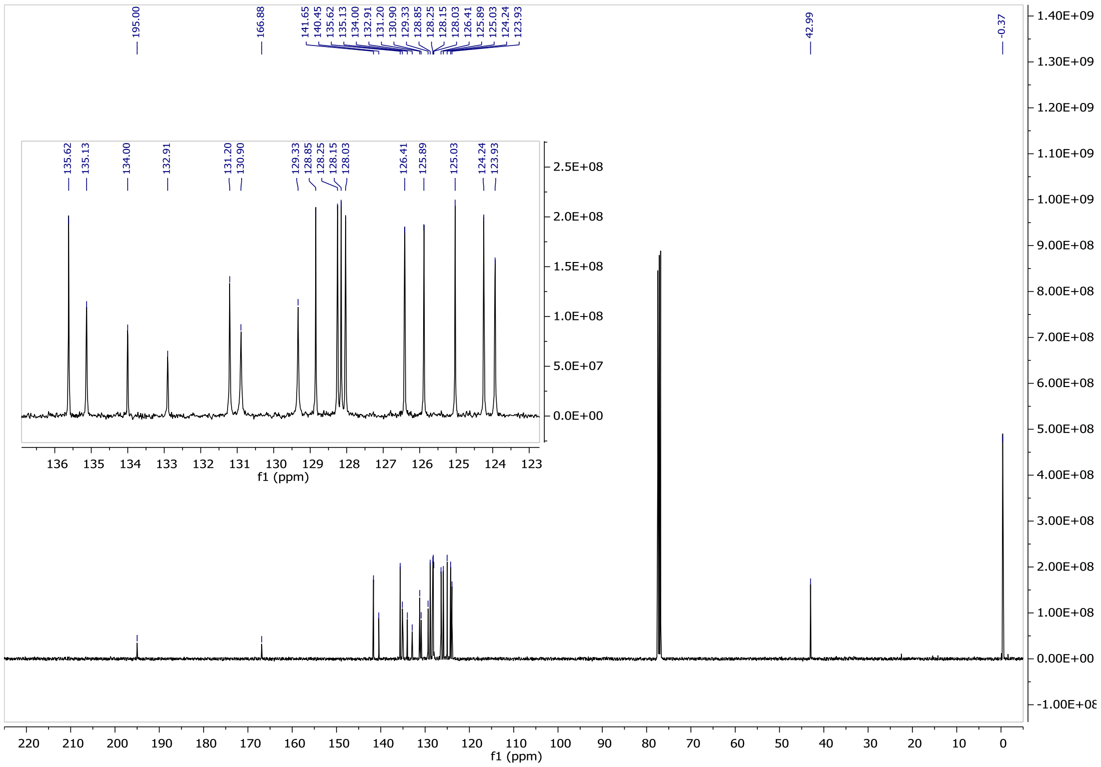

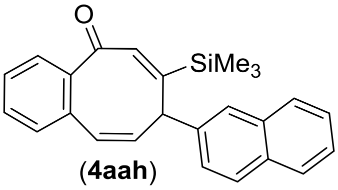

(**4aah**)

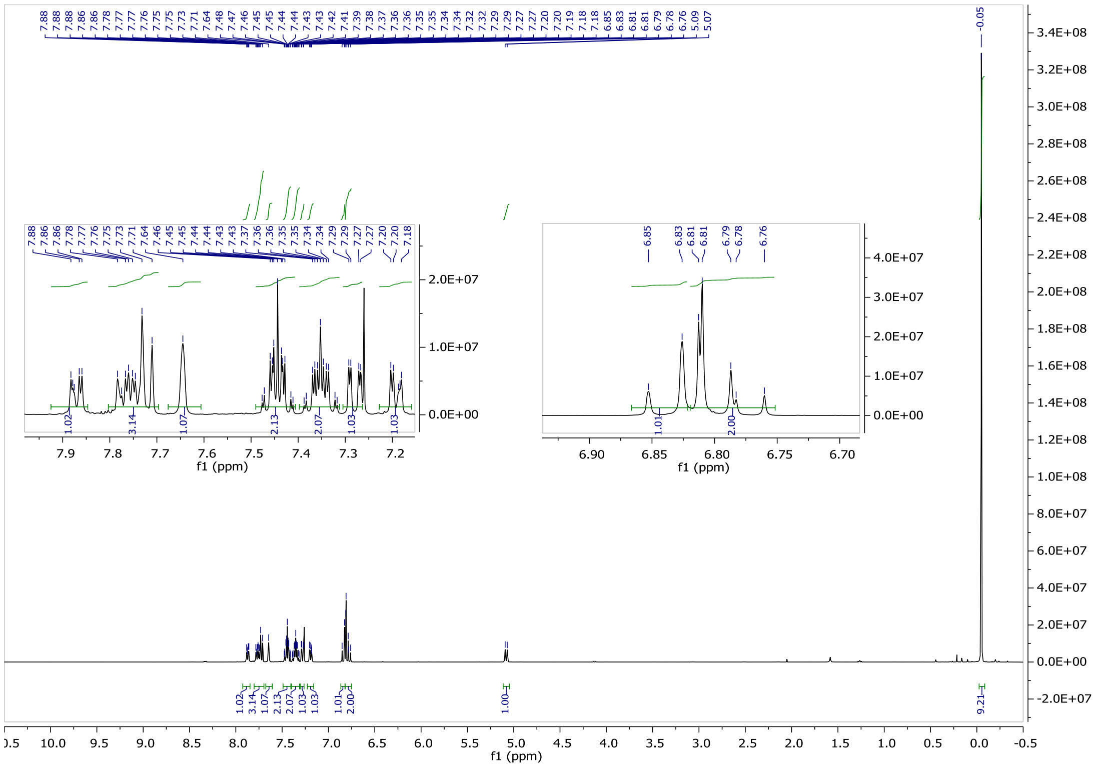

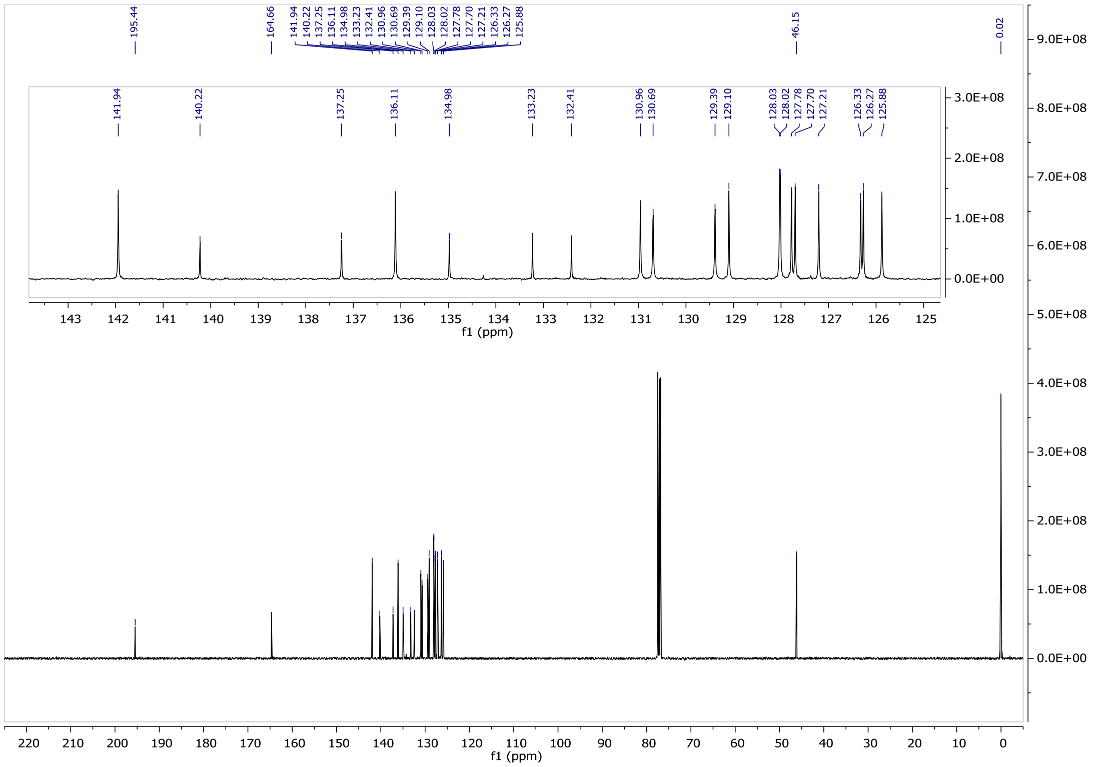

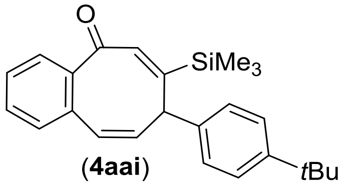
SiMe$_3$
tBu

(**4aai**)

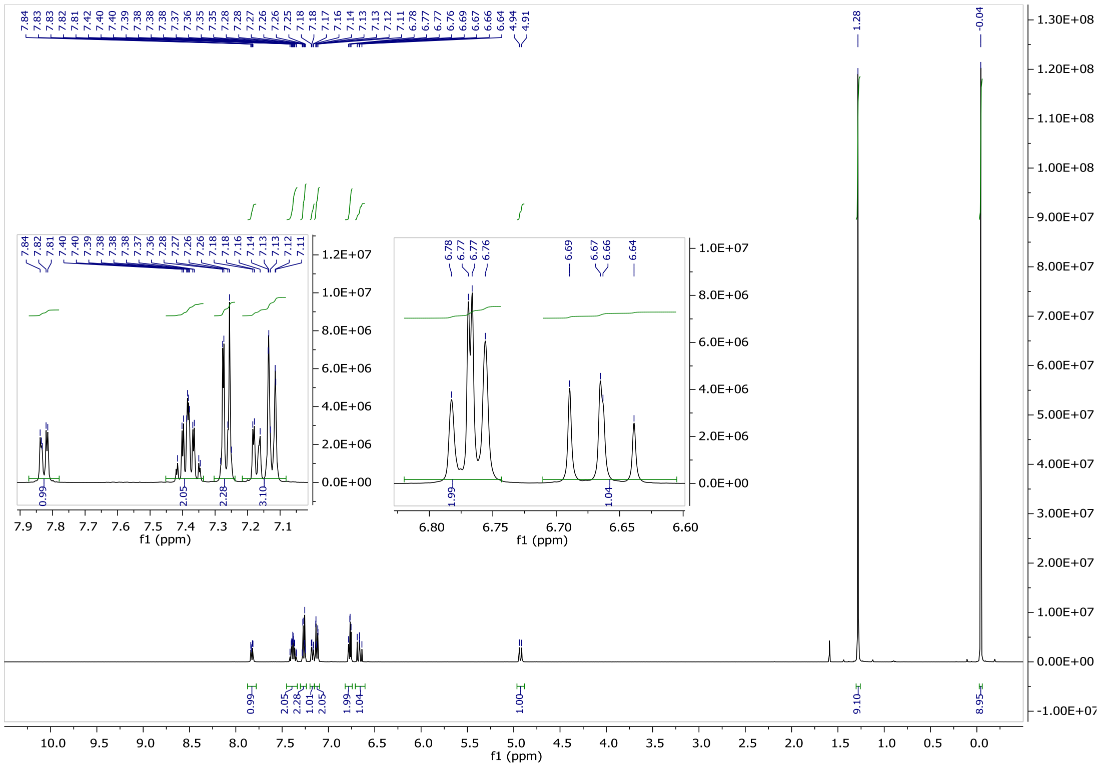
7.84
7.83
7.83
7.82
7.81
7.42
7.40
7.40
7.39
7.38
7.38
7.38
7.37
7.36
7.35
7.35
7.28
7.28
7.27
7.26
7.26
7.25
7.18
7.18
7.17
7.16
7.14
7.13
7.13
7.12
7.11
6.78
6.77
6.77
6.76
6.69
6.67
6.66
6.64
4.94
4.91
1.28
-0.04
0.99
2.05
2.28
1.01
2.05
1.99
1.04
1.00
9.10
8.95
f1 (ppm)

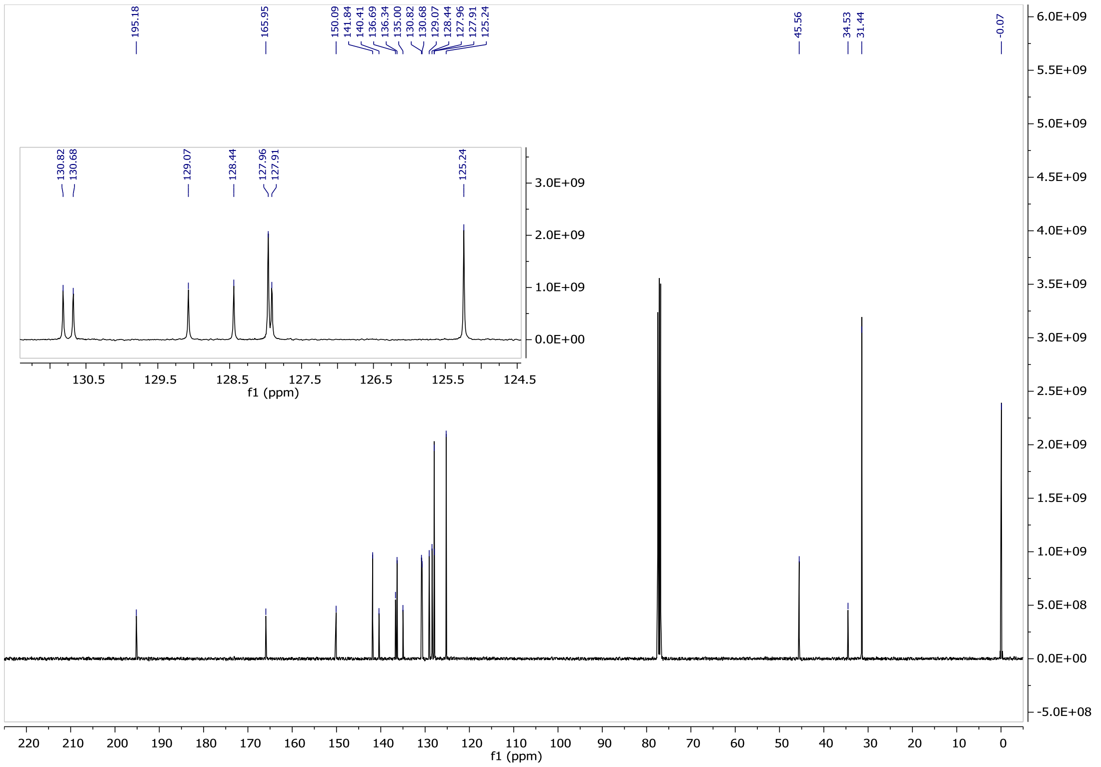
195.18
165.95
150.09
141.84
140.41
136.69
136.34
135.00
130.82
130.68
129.07
128.44
127.96
127.91
125.24
45.56
34.53
31.44
-0.07
f1 (ppm)

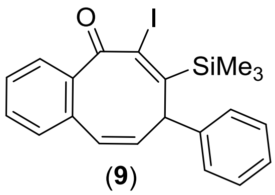

(**9**)

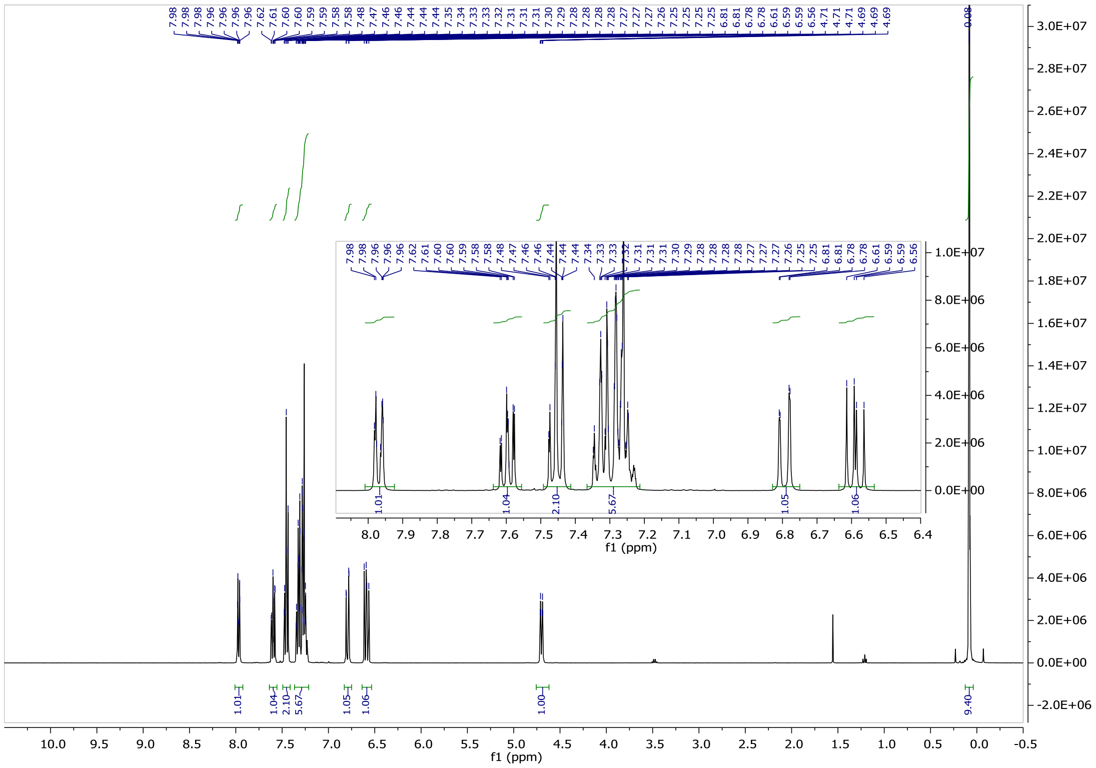

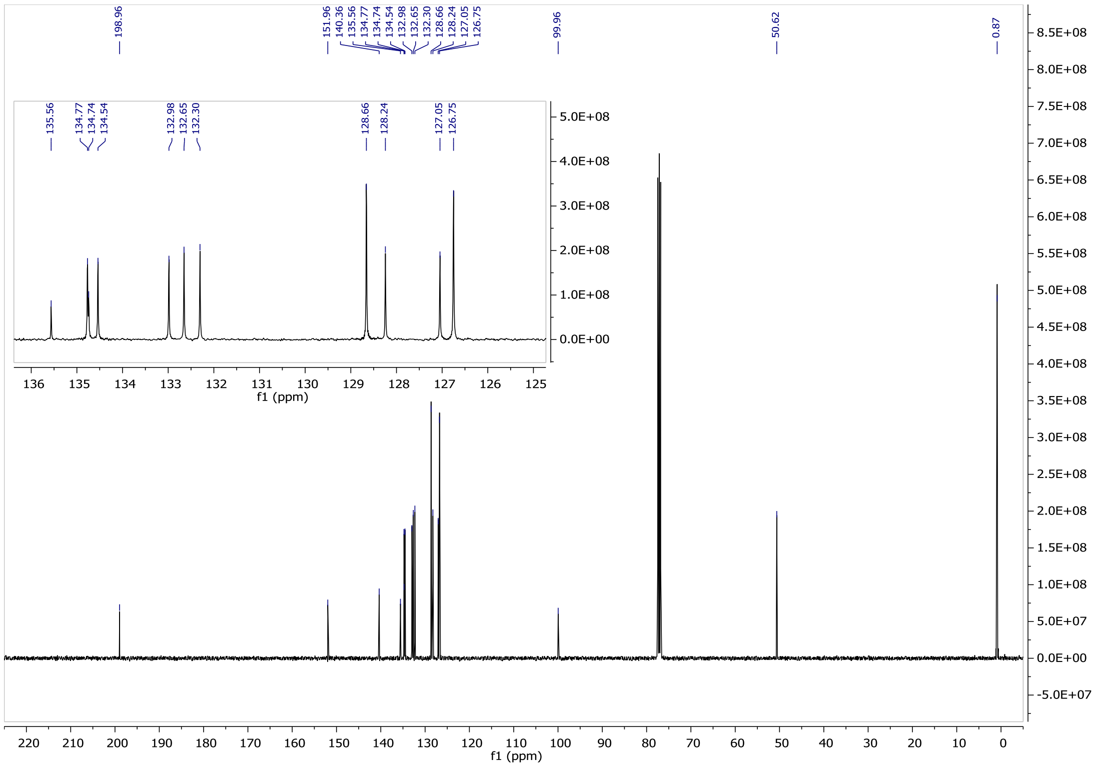

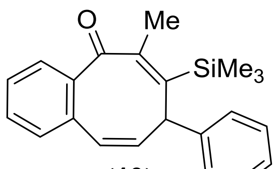
O
Me
SiMe3

(**10**)

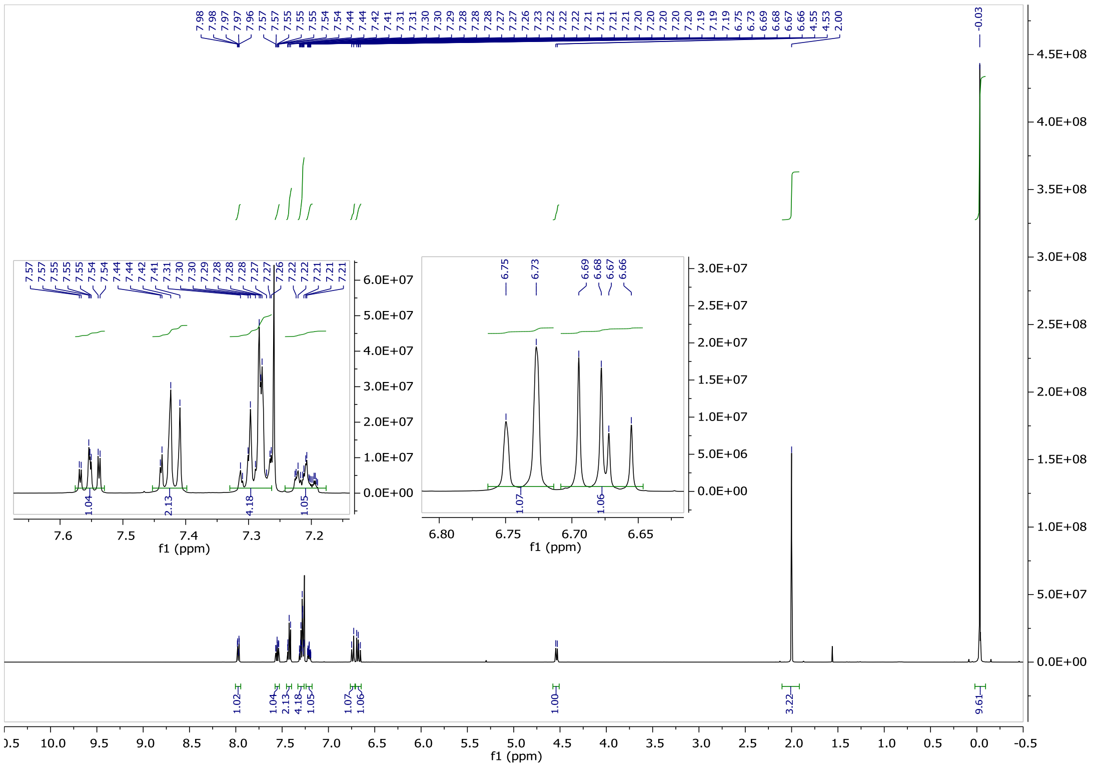
f1 (ppm)
1.02
1.04
2.13
4.18
1.05
1.07
1.06
1.00
3.22
9.61
-0.03

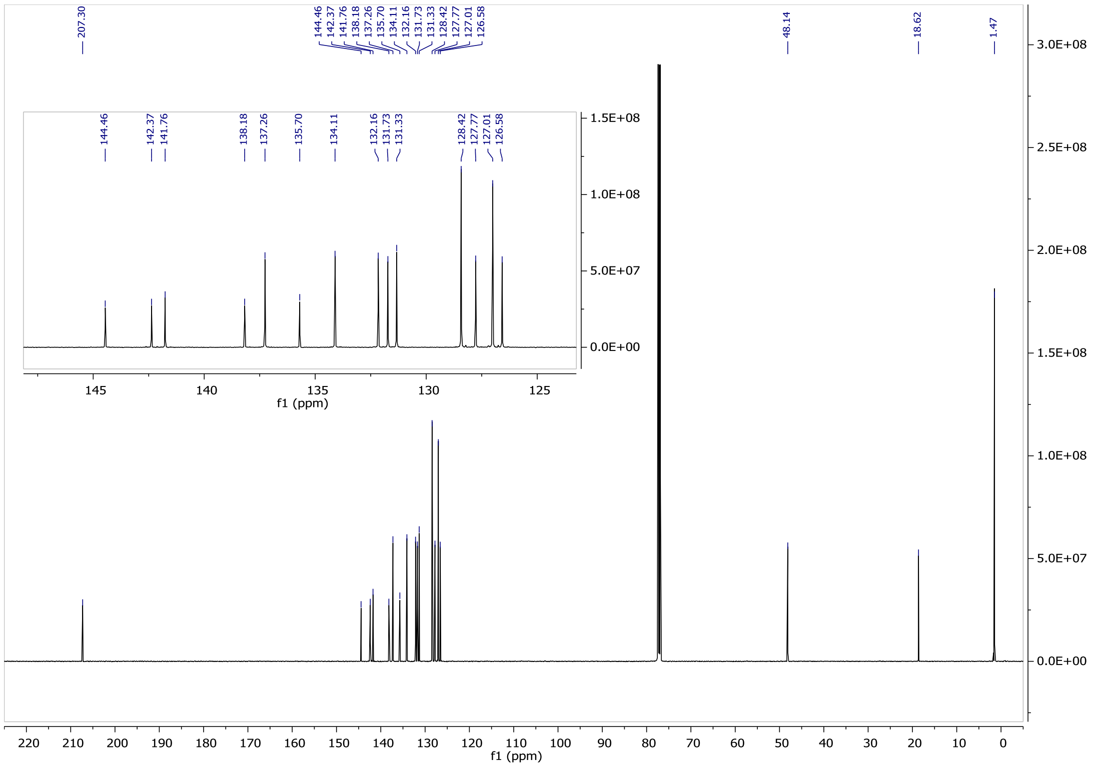
207.30
144.46
142.37
141.76
138.18
137.26
135.70
134.11
132.16
131.73
131.33
128.42
127.77
127.01
126.58
48.14
18.62
1.47
f1 (ppm)

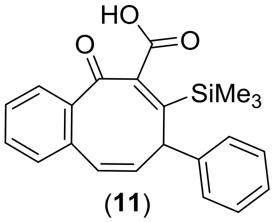
HO
O
O
SiMe3

(**11**)

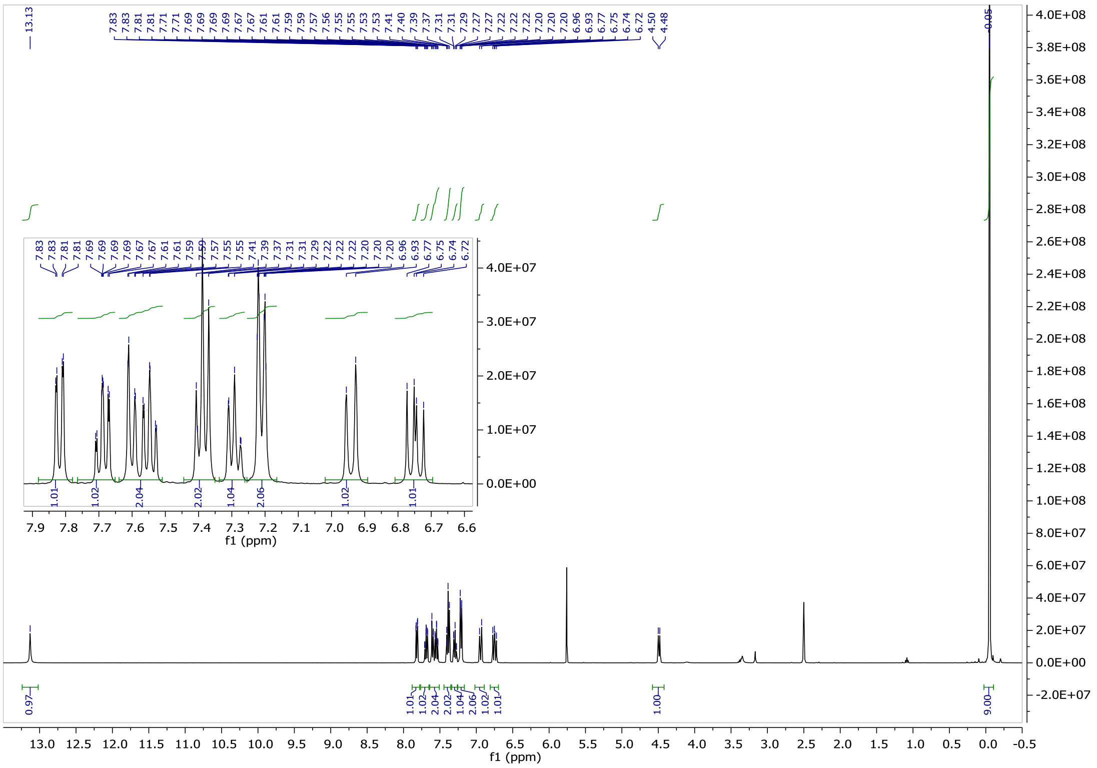
13.13
7.83 7.83 7.81 7.81 7.71 7.69 7.69 7.69 7.69 7.67 7.67 7.61 7.61 7.59 7.59 7.57 7.56 7.55 7.55 7.53 7.53 7.41 7.40 7.39 7.37 7.31 7.31 7.29 7.27 7.27 7.22 7.22 7.22 7.20 7.20 7.20 6.96 6.93 6.77 6.75 6.74 6.72
4.50
4.48
0.05
0.97
1.01 1.02 2.04 2.02 1.04 2.06 1.02 1.01
1.00
9.00
f1 (ppm)

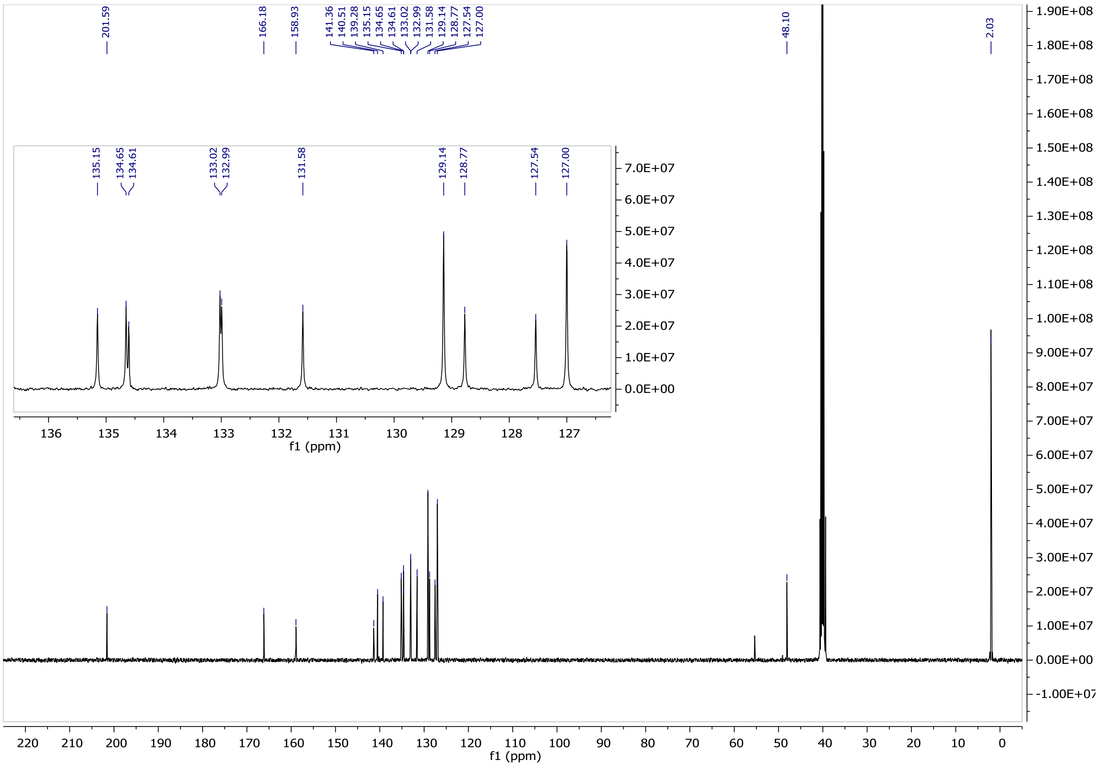
201.59
166.18
158.93
141.36 140.51 139.28 135.15 134.65 134.61 133.02 132.99 131.58 129.14 128.77 127.54 127.00
48.10
2.03
f1 (ppm)

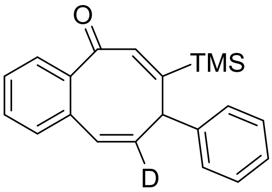
O
TMS
D

(d_1-4aaa)

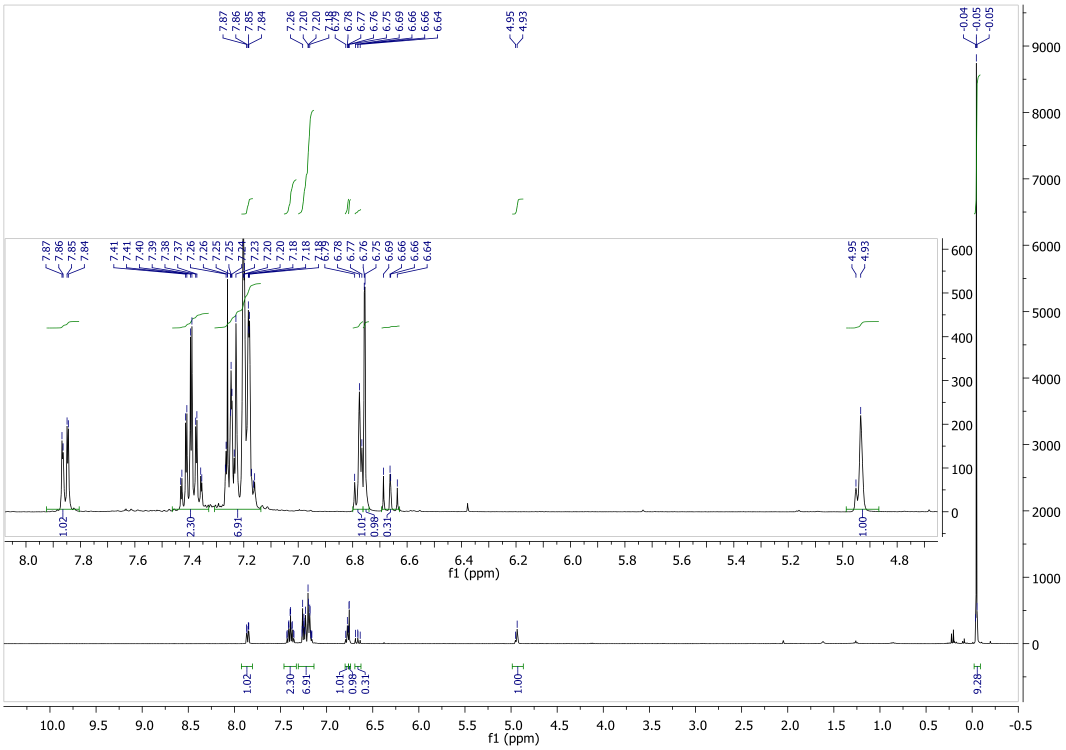
7.87
7.86
7.85
7.84
7.26
7.20
7.20
7.18
6.79
6.78
6.77
6.76
6.75
6.69
6.66
6.66
6.64
4.95
4.93
-0.04
-0.05
-0.05
1.02
2.30
6.91
1.01
0.98
0.31
1.00
9.28
f1 (ppm)

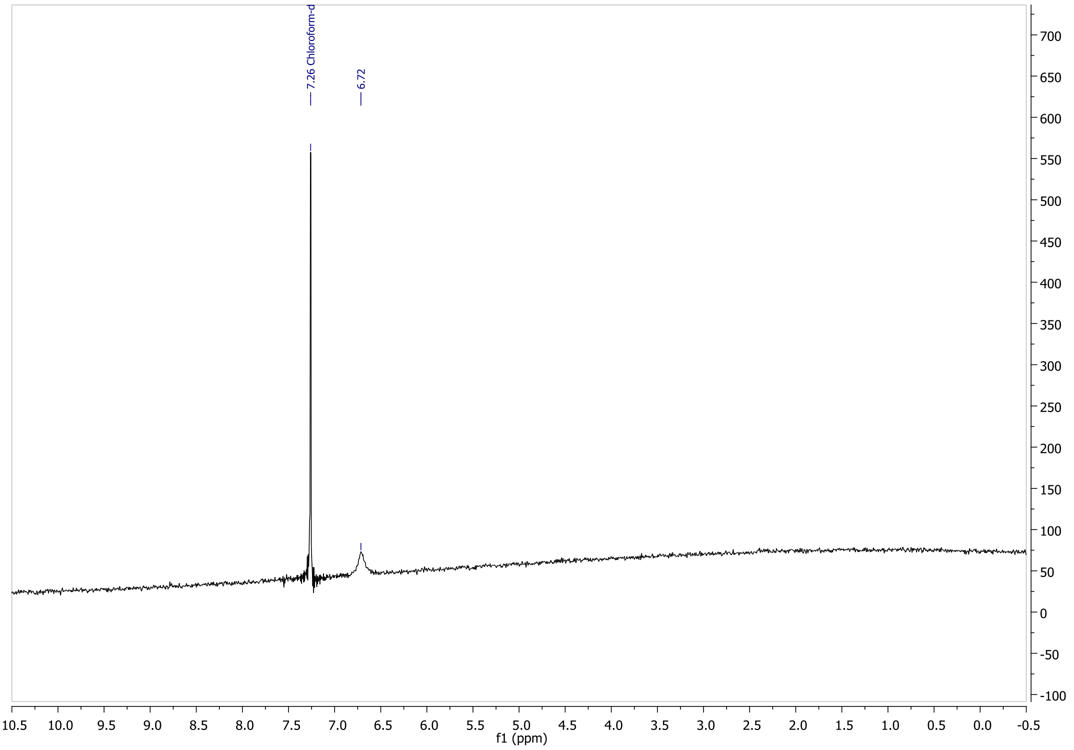
7.26 Chloroform-d
6.72
f1 (ppm)

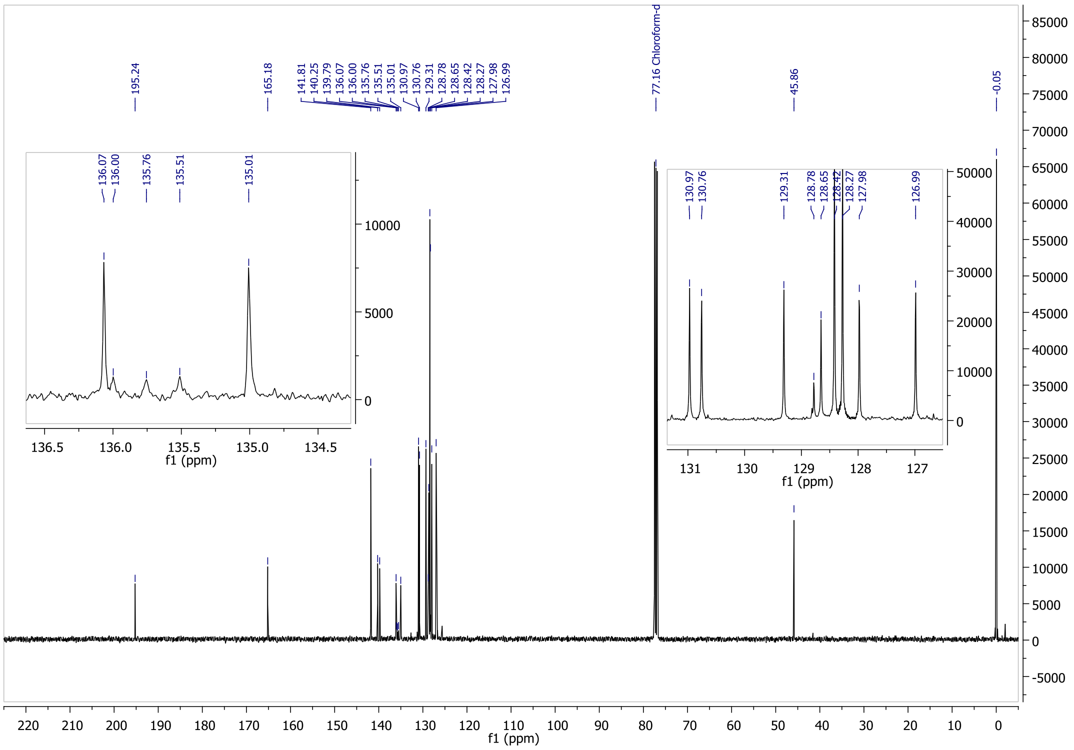

7. Selected Crystallographic Data

Figure S3. Molecular structure of **4aaa** confirming γ-addition and net hydride transfer.

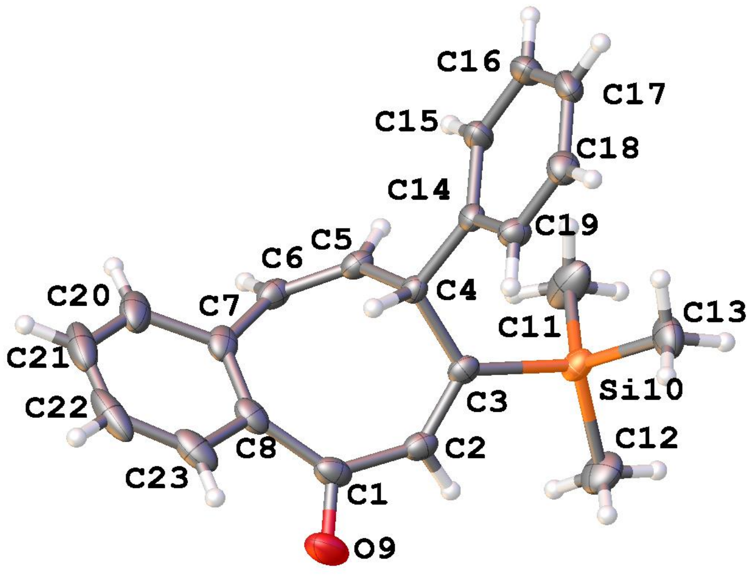

Figure S4. Molecular structure of **4haa** confirming γ-addition and net hydride transfer.

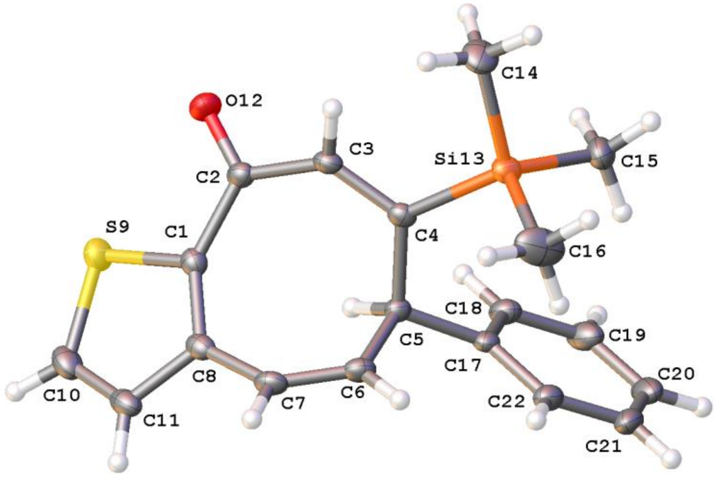

Figure S5. Molecular structure of **4jaa** confirming γ-addition and net hydride transfer.

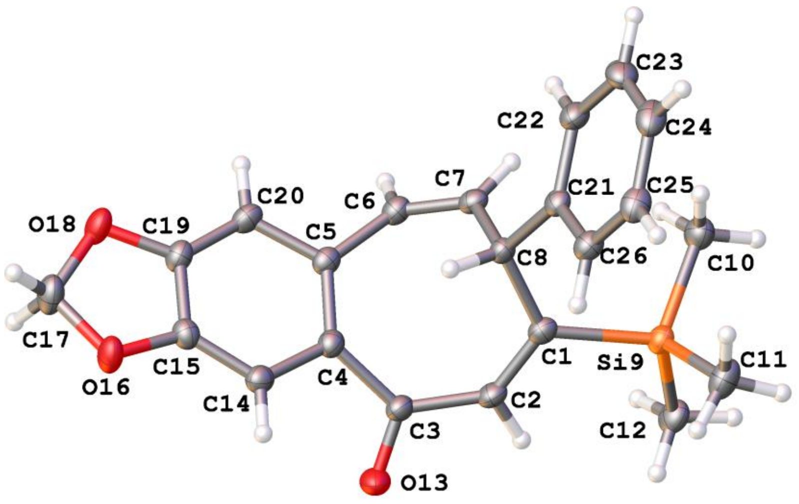

Figure S6. Molecular structure of **7** confirming capture of allene intermediate by 2-thienyl.

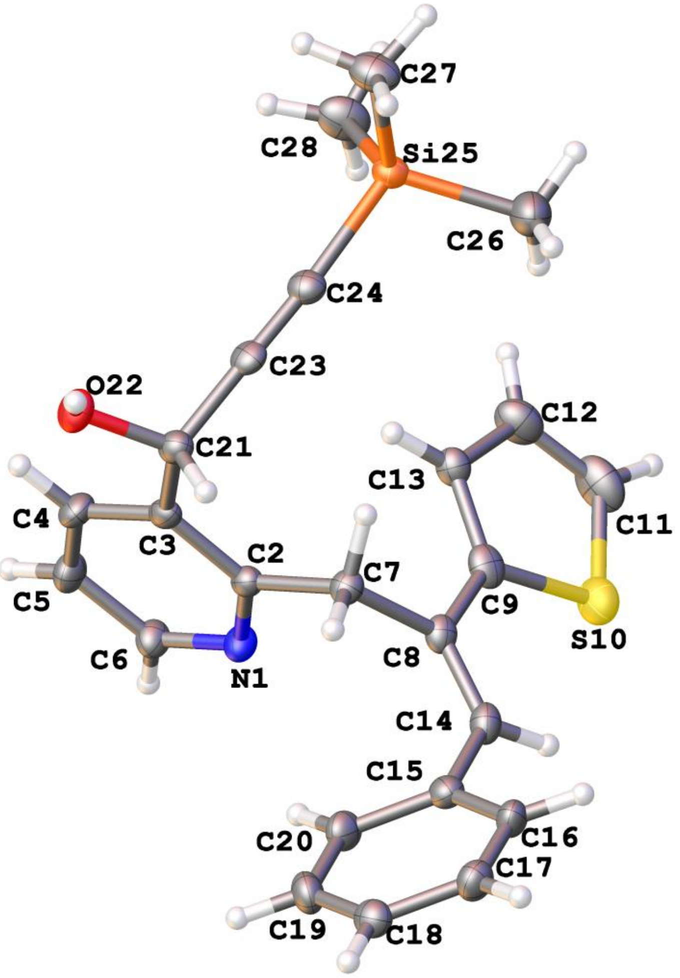

Supplement: Supplementary file 1 — miscellaneous_information [file anie0054-10648-sd1.pdf]
